# Limits of deep-learning-based RNA prediction methods

**DOI:** 10.1093/nar/gkag813

**Published:** 2026-08-24

**Authors:** Marko Ludaic, Arne Elofsson

**Affiliations:** Science for Life Laboratory and Department of Biochemistry and Biophysics, Stockholm University, Solna 171 21, Sweden; Science for Life Laboratory and Department of Biochemistry and Biophysics, Stockholm University, Solna 171 21, Sweden

## Abstract

In recent years, tremendous advances have been made in predicting protein structures and protein–protein interactions, but progress on RNA structure, alone or in complex with other macromolecules, has lagged behind despite recent methodological developments. It remains unclear whether reported gains in accuracy generalize to novel RNA structures. Here, we benchmark 10 deep learning methods on an independent, non-redundant dataset of monomeric RNAs and RNA complexes. For monomeric RNA, AlphaFold3 and Boltz-1 achieve the best overall performance, but accuracy depends greatly on similarity to the training set: predictions are reliable mainly for RNAs with well-defined or recurring folds, such as L-shaped and simple loop structures, while structurally divergent RNAs remain poorly modelled. Extending the evaluation to RNA complexes, AlphaFold3 and Boltz-1 substantially outperform HelixFold3 and RosettaFoldNA, with no statistically significant difference between the two leading methods on most measures. Together, these results show that current methods are constrained by limited, structurally biased training data and recognize recurring motifs rather than generalize to novel RNA folds.

## Graphical Abstract

**Graphical Abstract:**
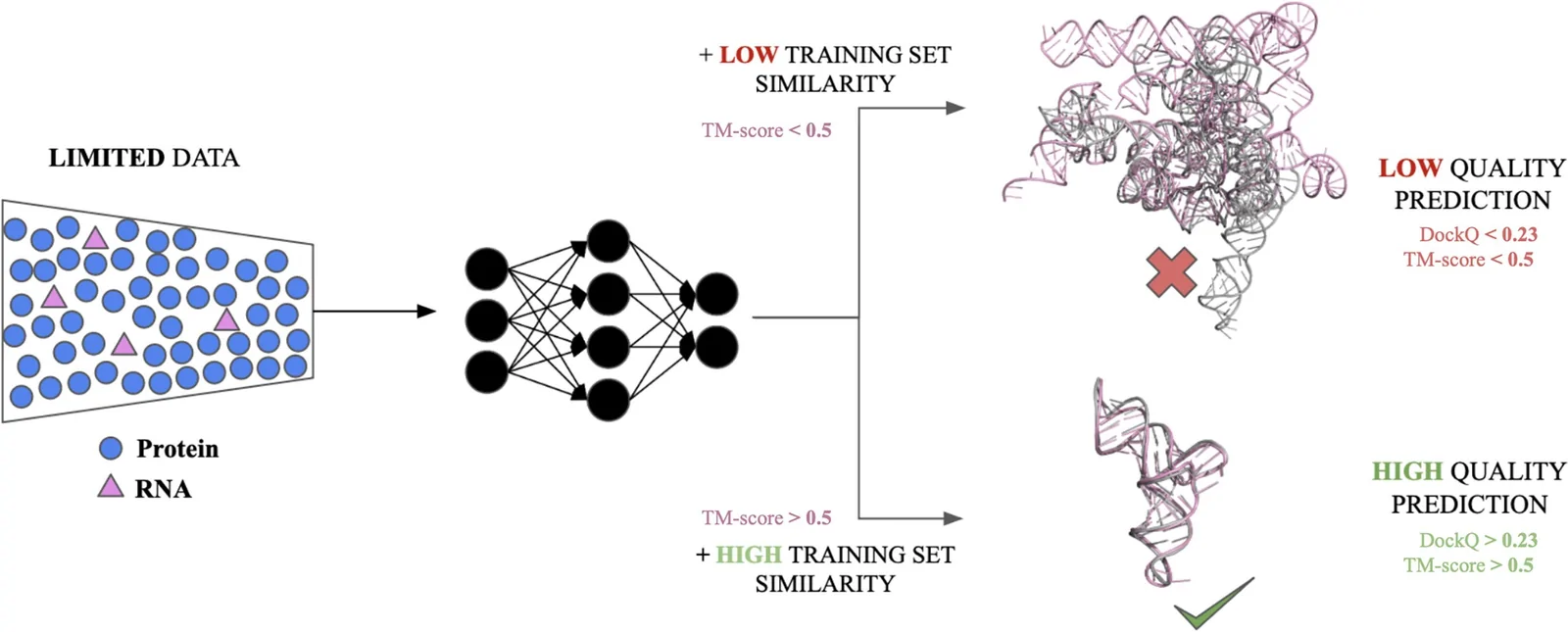

## Introduction

RNA molecules are highly dynamic macromolecules that adopt intricate three-dimensional (3D) structures essential to many cellular and viral processes, as well as to emergent RNA-based medicines and biotechnologies. Different types of RNA, such as antisense oligonucleotides, small interfering RNAs, and microRNAs, have shown promising therapeutic potential [1]. Even though RNAs were often viewed as mere intermediaries in the transfer of genetic information from DNA to protein synthesis, they are also recognized for their functional versatility, extending far beyond their linear nucleotide sequences.

RNA structures are critical for elucidating structure–function relationships; therefore, modelling RNA structure from its nucleotide sequences remains an important and largely unsolved task. There are two main reasons for this limited success. First, the limited availability of experimentally determined RNA structures, which is at least an order of magnitude lower than for proteins, poses a significant challenge for training artificial intelligence (AI)-based models. Secondly, RNA’s conformational dynamicity complicates the application of deep learning models. Many RNAs undergo drastic conformational changes to obtain a shape required for binding to a molecular partner [2]. This is also one of the reasons why experimental methods such as X-ray crystallography, nuclear magnetic resonance (NMR), and cryo-electron microscopy (EM) have primarily resolved regulatory and enzymatic RNAs, including tRNA, rRNA, and ribozymes [3]. The structure of other RNA types, such as long non-coding RNAs (lncRNAs), remains less well studied, possibly because they are indeed more disordered.

Before the release of RoseTTAFold2NA (RF2NA) in early 2024 [4] and AlphaFold3 (AF3) later in 2024 [5], deep learning approaches were limited to predicting the structures of monomeric RNAs and could not handle RNA in a complex with other molecules. Shortly thereafter, several deep learning approaches based on the AF3 architecture, including Boltz-1 [6], Chai-1 [7], and HelixFold3 (HF3) [8], were released. These methods can handle proteins, nucleic acids, small molecules, and ions, greatly expanding the scope and applicability of structure prediction. Furthermore, some methods for predicting monomeric RNA, such as RhoFold+ [9], utilize large language models (LLMs) to predict atom coordinates, while others are adaptations of AlphaFold. Table 1 provides an overview of the latest methods for predicting RNA structure.

**Table 1.** A brief description of the most critical characteristics of benchmarked methods for monomeric RNA and RNA–RNA/protein prediction

Method (short name)	Description	RNA	Training set Date cut-off
AlphaFold3 (AF3)	deep learning method consisting of a pairformer for residue interaction modelling and a diffusion module for denoising raw atomic coordinates, removing global rotational/translation constraints to enhance prediction flexibility and accuracy.	Monomeric, complex	30 Sep 2021
Boltz-1	AlphaFold3-inspired architecture with modified order of operations in the MSAModule and transformer layer.	Monomeric, complex	30 Sep 2021
Chai-1	AlphaFold3-inspired architecture incorporating residue-level embeddings from a large language model for proteins, also introducing experimental constraint features such as pocket, contact, and docking constraints. Trained without RNA multiple sequence alignments (MSAs).	Monomeric, complex	20 Jan 2021
DeepFoldRNA	RNA structure prediction method based on deep self-attention neural networks with gradient-based folding simulations	Monomeric	28 March 2022
DRfold	*Ab initio* RNA structure prediction method that integrates end-to-end learning with deep geometrical potentials.	Monomeric	30 Dec 2020
HelixFold3 (HF3)	AlphaFold3-inspired architecture using the PaddlePaddle library allowing for faster model training.	Monomeric, complex	30 Sep 2021
NuFold	A deep learning method built upon AlphaFold architecture designed to predict RNA structures by incorporating nucleic acid sequences, secondary structure inputs, and RNA-specific inter-base angles, distances, and atom positions.	Monomeric	28 Feb 2022
RhoFold+	A deep learning model for RNA structure prediction that utilizes an RNA language model, alongside several deep learning modules, including an invariant point attention (IPA) module for modelling 3D positions.	Monomeric	13 April 2022
RosettaFoldNA (RF2NA)	A three-track architectured model that simultaneously updates sequence (1D), residue-pair (2D), and structural (3D) representations to predict macromolecular structures.	Monomeric, complex	May 2020
trRosettaRNA	A deep learning model consisting of MSA and pair representations fed into a transformer network (RNAformer) predicting 1D and 2D geometries, refined in the final 3D structure folding step.	Monomeric	1 Jan 2017

If the date of the test-and-training data split was not mentioned in the paper, the paper’s release date was used instead. Methods are shown in alphabetical order.

Until recently, systematic benchmark studies directly comparing state-of-the-art methods have been scarce. Nevertheless, community-wide assessments such as RNA-Puzzles Round V [10] and the CASP16 (Critical Assessment of Structure Prediction) RNA assessment [11] have been conducted. These efforts are organized as competitions in which participating groups submit predictions for blind targets, with a focus on evaluating participants' performance rather than on a systematic comparison of available methods under identical input conditions. To our knowledge, one study [12] compared RNA structure prediction methods for monomeric RNA. However, it did not include many of the most recent approaches (e.g. Boltz-1, HF3, Chai, RF2NA, and NuFold). Additionally, a recent study benchmarked RNA–RNA interaction methods limited to secondary structure prediction [13], focusing on base pair prediction accuracy rather than 3D structure modelling. The most recent benchmark also included RNA–protein complexes, but compared only two methods, AF3 and RF2NA [14].

We conducted a comprehensive benchmark study to evaluate the capabilities and limitations of these deep learning methods for both monomeric RNA and RNA complexes. The analysis compared the performance of ten methods for predicting monomeric RNA structures and four methods for predicting RNA complexes (Table 1). By systematically assessing these models, we aim to provide valuable insights into their accuracy, strengths, and areas for further improvement.

## Materials and methods

### Benchmark dataset

RNA-containing entries released after 30 September 2021 (for RNA complexes) and 13 April 2022 (for monomeric RNAs), which correspond to the latest training set date cut-off of the benchmarked methods, were downloaded from the Protein Data Bank (PDB) along with their corresponding sequences in January 2026. For each entry, the first biological unit was used. In addition, we used RNA-containing CASP16 targets to supplement the benchmark dataset.

All sequences were filtered using CD-HIT-EST [15] at 80% sequence identity to ensure diversity in the datasets. The clustering was performed both within the benchmark dataset and against the remaining RNA sequences in the PDB deposited before the training date cut-off. Sequence identity was calculated using the longer sequence as the reference, which was critical because many RNA sequences in the PDB are synthesized from short RNAs. Applying this criterion avoids falsely reporting 100% sequence identity between a short and a long RNA sequence. Sequences containing ambiguous nucleotides, such as ‘X’ or ‘N’, were removed from the dataset. Furthermore, sequences shorter than 10 nt that consisted predominantly of a single nucleotide type (>80% homogeneity) were excluded, as they are likely to be linear and lack a modular structure, making them unsuitable for the benchmark dataset. Both experimentally determined and synthetic RNA structures from the PDB were preserved, as they were both a part of the training set for the benchmarked deep-learning-based methods of this study. These filtering criteria were applied to both monomeric RNAs and RNA sequences from RNA complexes.

**Figure 1.**
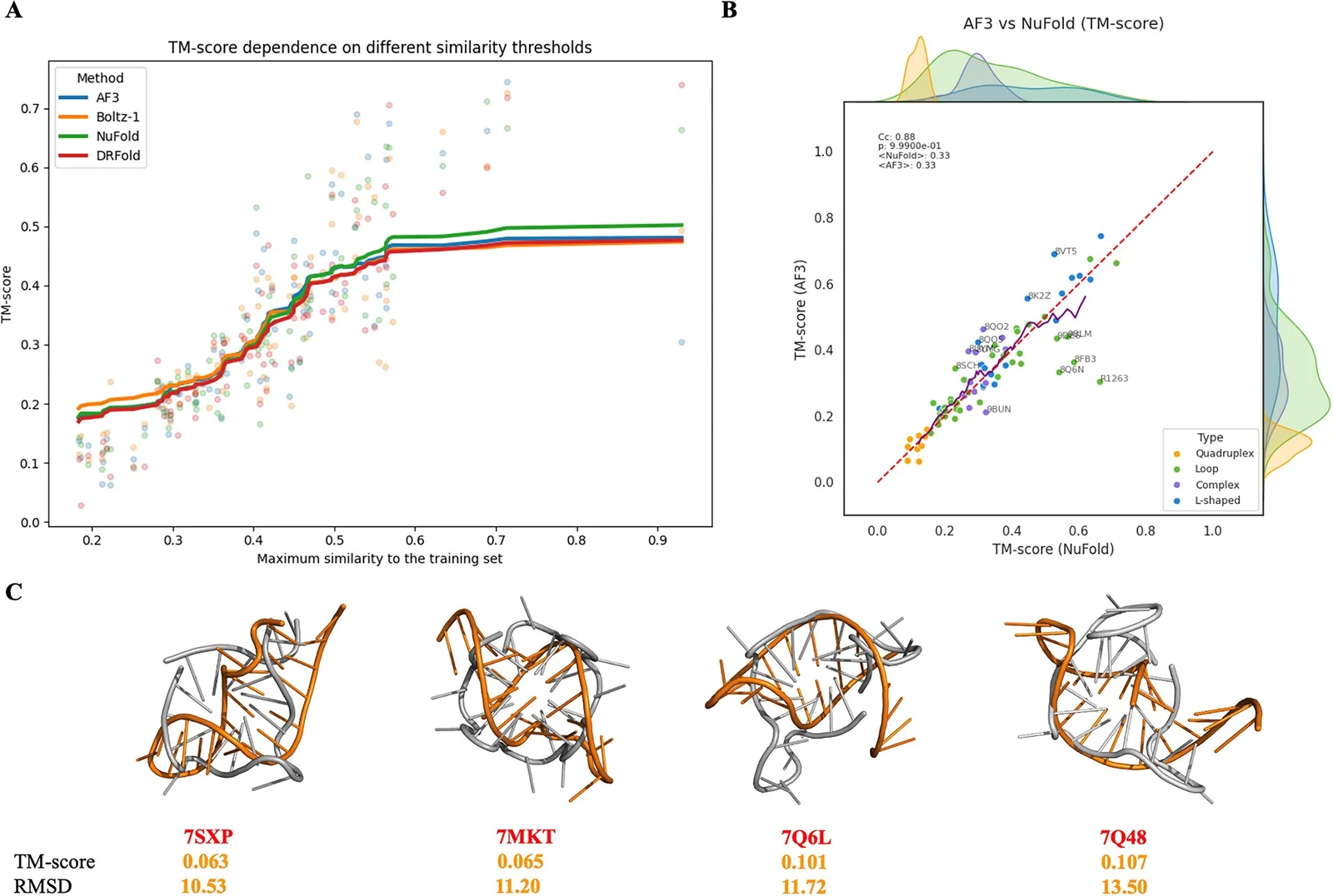
(**A**) TM-score dependence on maximum similarity to the training set for the top benchmarked methods. For each top-performing method, the running average is plotted in a different colour. (**B**) Pairplot showing the correlation between NuFold (*x*-axis) and AlphaFold3 (*y*-axis) TM-scores for monomeric RNA. The labelled points show PDB IDs of predicted models for which the TM-score difference between the two methods exceeds 10%. Paired *t*-test reported no significant difference between the two methods (Cc = 0.85, *P* = 9.97×10^–1^). (**C**) Visualization of the four least accurate AF3 predictions. Ground-truth structures are coloured in grey, while the predicted structures are shown in orange.

To evaluate structural redundancy, RNA-align TM-score (TM-scoreRNA) [16], a length-dependent variant of TM-score optimized for RNA molecules, was used. Structures of RNA complexes were divided into individual chains, and the RNA chains were extracted and processed together with monomeric RNA structures. Since there is no RNA-specific tool for structural similarity search equivalent to Foldseek [17] for proteins, the pairwise TM-score was calculated between all structures within the initial benchmark dataset, and those with a TM-score > 0.7 were considered structurally too similar and excluded from the benchmark dataset. Each case was also manually reviewed to confirm that TM-score values accurately reflect actual structural similarity. TM-scores were also calculated against the training set. This procedure finds structurally similar folds that models have seen during the training. We preserve those structures and discuss how dependent the model’s accuracy is on the training set (see ‘Performance of structure prediction methods on monomeric RNAs’). In addition, we show that retaining these models does not influence the final scoring and ranking of the methods (Fig. 1A). For RNA–protein complexes, protein structural similarity was not used as a filtering criterion, as it would reduce the dataset size. Instead, the filtering remained focused on RNA, consistent with the benchmark’s aims of evaluating RNA prediction accuracy, rather than protein structure prediction.

Finally, the dataset was further reduced because several sequences in the initial test set could not be modelled by specific methods. Modelling complexes with more than four protein and/or RNA subunits with Boltz-1 version 0.4.1 failed due to out-of-memory errors. Consequently, such complexes were excluded from the analysis, reducing the benchmark dataset for complexes to 158 structures. After filtering, our benchmark dataset for monomeric RNA structures comprised 79 models.

### Model generation

The benchmark evaluates the performance of 10 prediction methods, AF3 [5], Boltz-1 [6], Chai-1 [7], HF3 [8], NuFold [18], RhoFold+ [9], RF2NA [4], trRosettaRNA [19], DRfold [20] , and DeepFoldRNA [21] for monomeric RNA. Four of the methods: AF3, Boltz-1, HF3, and RF2NA, also predicted RNA–RNA and RNA–protein complexes. Chai-1, a multimodal foundation model for molecular structure prediction, was excluded from the RNA complex analysis due to its 2048-token inference limit. Furthermore, three of the longest monomeric RNAs from the initial benchmark dataset were successfully predicted by the other methods, but were not generated during DRfold and DeepFoldRNA inference under the software versions and parameters used in this study. To maintain a consistent set of targets across all methods for the main comparative analysis, three targets were excluded: 8BU8 (354 nt), 8TVZ (363 nt), and the CASP16 R1248 target (407 nt). All methods were run using default settings. Multiple sequence alignments (MSAs) were provided externally, as explained below, in case the method allowed for custom MSAs. All software versions are given in Supplementary Table S1.

For each method, the structures were generated on a single NVIDIA DGX-A100 Core GPU using the latest version (v1.0 for trRosettaRNA) as of 1 October 2025, with default settings. For all methods, the same set of MSAs was used. These were generated using rMSA [22] for RNA sequences and MMseqs2 [23] for protein sequences. An rMSA search was performed against the RNAcentral [24] and the NCBI Nucleotide (nt) databases using default settings. For proteins, MMseqs2 was run on the ColabFold databases as of 1 October 2025, which consist of UniRef30, BFD/Mgnify, and ColabFold DB [25]. MSAs were converted into alternative formats depending on each model’s specific requirements. The conversion scripts are available from the accompanying git repository. For each method, five models with the same seed were generated for each target.

The top-ranked model was used to analyse each method. Several methods, including AF3, Boltz-1, HF3, and Chai-1, report predicted template modelling (pTM) and interface predicted template modelling (ipTM) scores in their outputs and internally rank models. For example, for AF3, the top-ranked model was selected using the native AF3 ranking_score. Other methods, such as RF2NA or RhoFold+, report the predicted local distance difference test (pLDDT), which reflects confidence in the local structure [26], for each model. For these methods, we ranked models by their average pLDDT scores. For DRfold, which outputs only a single refined final model, we selected this model for the analysis. If a method did not report any confidence or ranking score, we selected the first structure. This approach aligns with the practical goal of the top model in the output, ideally representing the best prediction. We also noted that, in almost all cases, the quality difference between generated models is minimal (Supplementary Figs S1–S4). In addition, we calculated mean TM-scores per method by considering both the top-ranked and/or first-generated structures and the best-generated model in the decoy set. We show that the difference between these two approaches is minimal and non-significant (Supplementary Fig. S5). The largest difference in TM-score between the two approaches was 0.137 for 8VCI using AF3. In the case of Boltz-1, the highest difference was 0.238 for 8K30.

### Model evaluation

To evaluate the quality of predicted models, we used four different evaluation metrics that were also used in the latest CASP16 assessment of 3D RNA structure [11] . More specifically, for monomeric RNAs, we calculated the TM-score using RNA-align, Interaction Network Fidelity (INF), Local Distance Difference Test (lDDT), and a Global Distance Test (GDT-TS). To calculate INF, lDDT, and GDT-TS, we used RNAdvisor [27], a comprehensive benchmarking tool for measuring and predicting the quality of RNA structural models. To assess RNA complexes, in addition to the US-align TM-score, we also used the DockQ score [28], F1 score, fnat, iRMSD, and LRMSD, which are all reported during DockQ score calculation. DockQ provides an overall score, averaged across all interfaces, as well as individual scores, one for each interface. We also assessed the accuracy of RNA–RNA and RNA–protein interfaces within each complex, using the per-interface DockQ score. In PDB entries, the deposited sequence (provided in FASTA/SEQRES records) does not always fully correspond to the experimentally resolved structure, as flexible or disordered regions may be missing in the atomic coordinates. As a result, predicted models and reference structures may differ in length or residue composition. To ensure a consistent and fair comparison, predicted structures were trimmed to match the resolved residues present in the reference structure prior to evaluation.

We calculate the TM-score using the following command-line options:

RNAalign {model.pdb} {reference.pdb}

for monomeric RNA molecules,

USalign {model.pdb} {reference.pdb} -mm 1 -ter 0

for RNA complexes, and

USalign {model.pdb} {reference.pdb}

for monomeric protein molecules.

We have noticed that TM-score underestimates the accuracy of shorter RNAs in our benchmark dataset and have therefore conducted further analysis to investigate this behaviour. The analysis is reported in the Supplementary Material (section ‘Length-Dependent Limitations of TM-score in RNA Structure Evaluation’).

For RNA complexes, to assess whether and how accurately the binding sequence was predicted, we also calculated the F1 score, which combines precision and recall into a single metric. We defined interface residues as those with at least one atom within 5 Å of any atom of the interacting partner chain. Model residues were then extracted and mapped onto the corresponding reference residues by sequence alignment. We then quantified the recovery of the protein- and RNA-binding sites using the F1 score (protein-binding site F1; RNA-binding site F1).

To calculate the F1 score, true positives (TPs) were defined as residues identified as interface residues in both model and reference structures, false positives (FPs) as RNA residues predicted to be only at the interface of a model structure, and false negatives (FNs) as residues found only at the interface of a reference structure that were therefore missed during structure prediction. The calculation was performed after aligning a pair of sequences between the model and the reference structure.

Precision and recall were calculated as:


\begin{eqnarray*}
\textit{Precision}\, = \,\frac{{TP}}{{\left( {TP + FP} \right)}},\,\textit{Recall}\, = \,\frac{{TP}}{{\left( {TP + FN} \right)}}
\end{eqnarray*}

The F1 score was then calculated as the harmonic mean of precision and recall:


\begin{eqnarray*}
F1\, &=& \,2\, \times \frac{{\left( {\Pr \textit{ecision}\,x\,{\mathop{\mathrm{Re}}\nolimits} \textit{call}} \right)}}{{\left( {\Pr \textit{ecision}\, + \,{\mathop{\mathrm{Re}}\nolimits} \textit{call}} \right)}},\,\,\textit{equivalently}:\,\\&& F1\, = \,\frac{{2TP}}{{\left( {2TP + FP + FN} \right)}}
\end{eqnarray*}

## Results and discussion

### Performance of structure prediction methods on monomeric RNAs

We divided the test set into easy and hard sets based on the maximum similarity to any entry in the PDB training data, using a TM-score cut-off of 0.5. The performance of the different methods for predicting monomeric RNAs is summarized in Tables 2 (easy) and 3 (hard). Here, we defined success rates using thresholds (TM-score > 0.45, GDT-TS > 0.45, INF > 0.75, and lDDT > 0.75) for each metric in the same way as it was done in the CASP16 evaluation of nucleic acids [11] to detect successful predictions. Scores for all models are shown in Supplementary Tables S2–S5.

**Table 2.** Monomeric RNA structure evaluation for the easy dataset, sorted by TM-score

Easy targets	TM-score	GDT-TS	INF	lDDT
NuFold	**0.482**	**0.610**	0.745	0.599
AF3	**0.468**	**0.585**	**0.777**	**0.693**
Boltz-1	**0.461**	**0.598**	**0.731**	0.609
DRfold	**0.458**	**0.559**	0.736	*0.481*
DeepFoldRNA	0.428	**0.559**	0.736	*0.481*
RF2NA	*0.408*	0.538	0.698	*0.377*
Chai	*0.402*	*0.520*	0.721	0.643
HF3	*0.381*	*0.512*	0.758	0.610
RhoFold+	*0.377*	*0.539*	0.692	0.614
trRosettaRNA	*0.342*	*0.459*	*0.538*	0.581

The best method for each score is underlined. Methods not statistically different from the best method are shown in bold. Methods not statistically different from the worst method are shown in italic.

We compared the average scores using four different measures and assessed whether the observed differences between methods were statistically significant using a Wilcoxon signed-rank test (Supplementary Fig. S6). For the easy dataset (Table 2), NuFold achieved the highest mean performance according to TM-score (0.482) and GDT-TS (0.610), while AF3 was the best model based on INF (0.777) and lDDT (0.693). Here, no significant differences in TM-score and GDT-TS were observed among AF3, NuFold, Boltz-1, and DRfold, whereas AF3 is significantly better than all other methods in lDDT and INF (except for Boltz-1). On the hard dataset (Table 3), Boltz-1 achieved the highest TM-score (0.247), GDT-TS (0.437), and INF (0.673), while AF3 showed the best performance measured by lDDT (0.553). The performance of HF3 was statistically indistinguishable from that of Boltz-1 and AF3 across the measures. trRosettaRNA performed significantly worse than most other methods on both datasets, as measured by INF.

**Table 3.** Monomeric RNA structure evaluation for the hard dataset, sorted by TM-score

Hard targets	TM-score	GDT-TS	INF	lDDT
Boltz-1	**0.247**	**0.437**	**0.673**	0.500
AF3	**0.245**	**0.433**	**0.669**	**0.553**
DRfold	**0.237**	**0.436**	0.617	0.361
NuFold	**0.236**	0.417	0.628	0.456
RF2NA	**0.236**	0.412	0.668	*0.286*
HF3	**0.230**	**0.417**	**0.663**	0.478
Chai-1	0.226	0.406	0.650	0.515
DeepFoldRNA	0.226	**0.436**	0.617	0.361
RhoFold+	*0.223*	0.409	0.610	0.508
trRosettaRNA	*0.198*	*0.369*	*0.492*	0.474

The best method for each score is underlined. Methods not statistically different from the best method are shown in bold. Methods not statistically different from the worst method are shown in italic.

In summary, AF3 is not significantly worse than the best method on any measure or dataset, and is statistically better than all other methods on the lDDT measure across both datasets. For all other measures, Boltz-1 performed on a par with AF3, while NuFold showed performance on a par with the best methods on the easy dataset and HF3 on the hard dataset. In addition, the performance decreased from the easy to the hard dataset across all metrics and methods, indicating that performance depends on similarity to the training set. These observed differences in prediction accuracy between the two datasets suggest that deep-learning-based methods can exploit structural similarity to previously observed RNAs, which probably contributes to their strong performance on the easy dataset. However, their substantially lower accuracy on the hard dataset indicates that they do not generalize well to RNAs with limited similarity to known structures, suggesting that current methods have not fully captured the underlying physics of RNA folding.

To further examine whether the decline in performance from the easy to the hard dataset was driven by structural similarity to known training set examples, we conducted an additional sensitivity analysis based on the maximum structural similarity between each target and the training set. For each target, we plotted the achieved TM-score against the maximum similarity to the training set (Fig. 1A). A gradual increase in prediction accuracy with increasing training set similarity can be observed, indicating that targets more similar to the training set are generally easier to predict. This highlights the limitation of the current methods in predicting unseen, structurally divergent RNAs. Moreover, the relative behaviour of the top-performing methods remained broadly consistent across the range of similarity.

Furthermore, examining success rates using previously defined thresholds, we observed that the percentage of models deemed correct varied substantially across evaluation metrics. When comparing easy and hard datasets, success rates decreased substantially, with TM-score and lDDT showing the largest drops (Supplementary Tables S6 and S7). In contrast, INF and GDT-TS maintained relatively higher success rates on the hard dataset than the other metrics. lDDT is much more restrictive than INF among the local measures, probably due to an lDDT threshold of 0.75 being strict, in particular for models generated by some methods (trRosettaRNA and RF2NA).

To better understand performance differences across RNA types, we plotted the TM-scores of AF3 against those of all other methods, combining the easy and hard datasets. When RNAs were grouped into four structural classes: RNA loops (41 RNAs), G-quadruplexes (9 RNAs), L-shaped conformations such as tRNAs (16 RNAs), and complex RNAs composed of multiple secondary structure motifs (13 RNAs), we noticed apparent differences in prediction accuracy (Fig. 1B). In general, RNAs with well-defined and relatively simple folds were predicted more accurately than structurally complex RNAs. Structures that adopt common folds well represented in PDB, such as L-shaped RNAs, were modelled most accurately by all methods (Supplementary Figs S7 and S8). In contrast, small G-quadruplex RNAs consistently ranked among the worst-scoring targets.

The four lowest-scoring monomeric RNAs generated using AF3 are shown in Fig. 1C. When the predicted models are superposed onto ground-truth structures, it is evident that AF3 recognizes the quadruplex fold but fails to model it correctly. In addition, one G-quadruplex RNA, 7SXP, was modelled slightly less inaccurately by Boltz-1 (TM-score = 0.237) than by AF3 (TM-score = 0.063). The structure 7SXP was released in May 2023 and was not a part of the training set for either method. To assess whether structurally similar homologues were present in the database, we performed a structural similarity search using TM-score against all G-quadruplex structures obtained from PDB. The highest similarity to 7SXP was observed with 7Q48 (TM-score 0.311), followed by 3UYH (TM-score 0.305). These results suggest that no structure in the database was sufficiently similar to 7SXP to bias model training or directly influence the generation of the structure. Although Boltz-1 achieved higher GDT-TS (0.79) and INF (0.42) values for 7SXP, the overall difference in model quality is moderate, and TM-scores remain low for both models (Supplementary Fig. S9). This behaviour is consistent with the limitation of TM-score when applied to short RNAs (Supplementary Material, section ‘Length-Dependent Limitations of TM-score in RNA Structure Evaluation’) and G-quadruplex structures.

### Defining a successfully predicted RNA model

**Figure 2.**
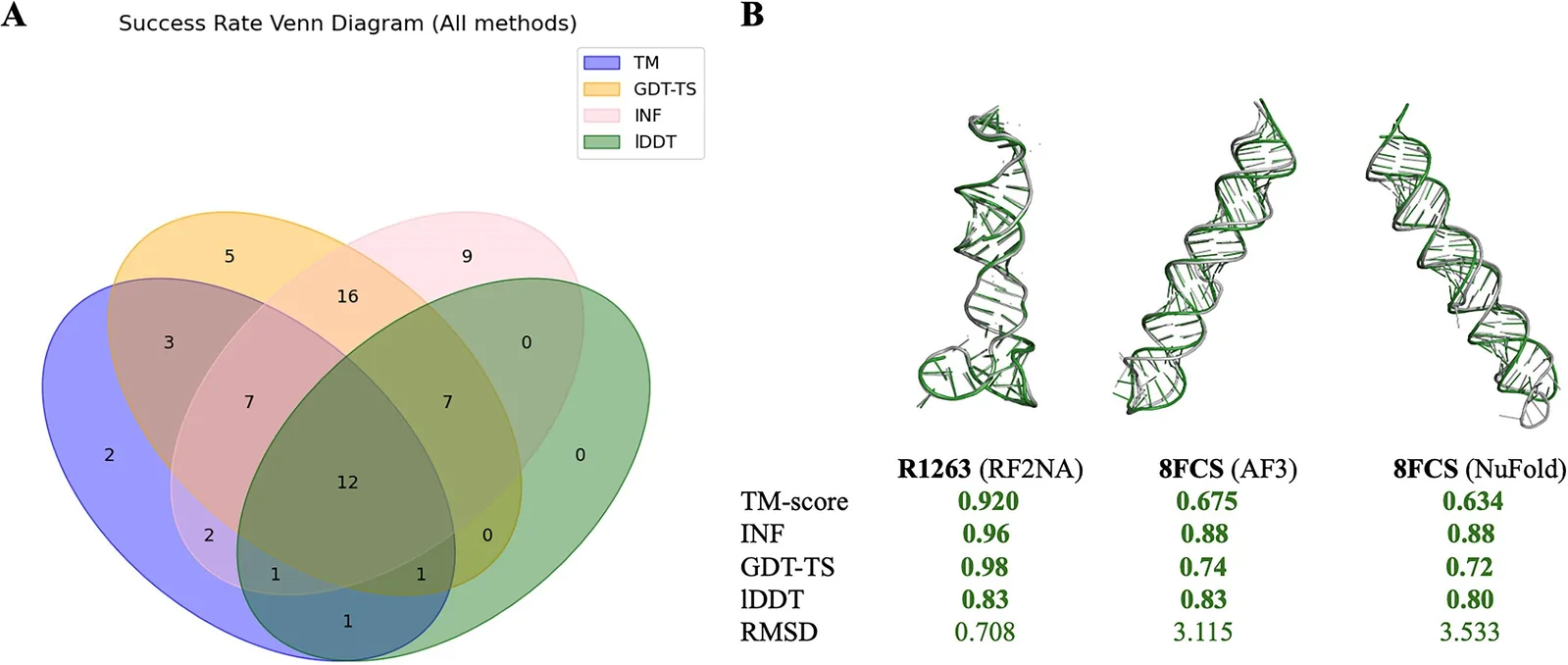
(**A**) Venn diagram showing the number of models passing the thresholds for different evaluation metrics, for all predictions across all methods. Each colour represents one metric. (**B**) The top three predicted structures according to the defined success thresholds.

We consider a prediction successful if any individual model exceeds the pre-defined success thresholds for each metric. We then analyse the overlap between these criteria using Venn diagrams, quantifying how many structures are consistently classified as successful across multiple metrics. When aggregating all predictions across all methods, only 12 structures satisfy the success thresholds for all four metrics simultaneously (Fig. 2A). Out of the 12 most successful predictions, the top three predictions shown in Fig. 2B include one tRNA-like structure (R1263) and two helical RNA structures (8FCS modelled by AF3 and 8FCS modelled by NuFold). In contrast, 66 predictions meet a threshold on at least one metric, and 42 predictions are classified as correct using both INF and GDT-TS, suggesting that individual metrics alone may overestimate accuracy. These results underscore the importance of using multiple evaluation metrics for reliable assessment of RNA structure prediction quality. Venn diagrams for individual methods are given in Supplementary Figs S10–S12.

### Model quality dependence on multiple sequence alignment depth

Many deep-learning methods rely on MSAs to capture evolutionary relationships, detect distant homologues, and improve model accuracy. To improve cross-method comparability, we used a uniform MSA input for methods that accept MSAs as external input, as mentioned in ‘Model generation’. This choice was made to reduce differences arising from method-specific MSA-generation pipelines, thereby focusing the benchmark primarily on each method’s structure prediction component. In particular, using a common MSA prevents one method from gaining an advantage simply because its default pipeline retrieves more homologous sequences or uses a differently filtered sequence database.

**Figure 3.**
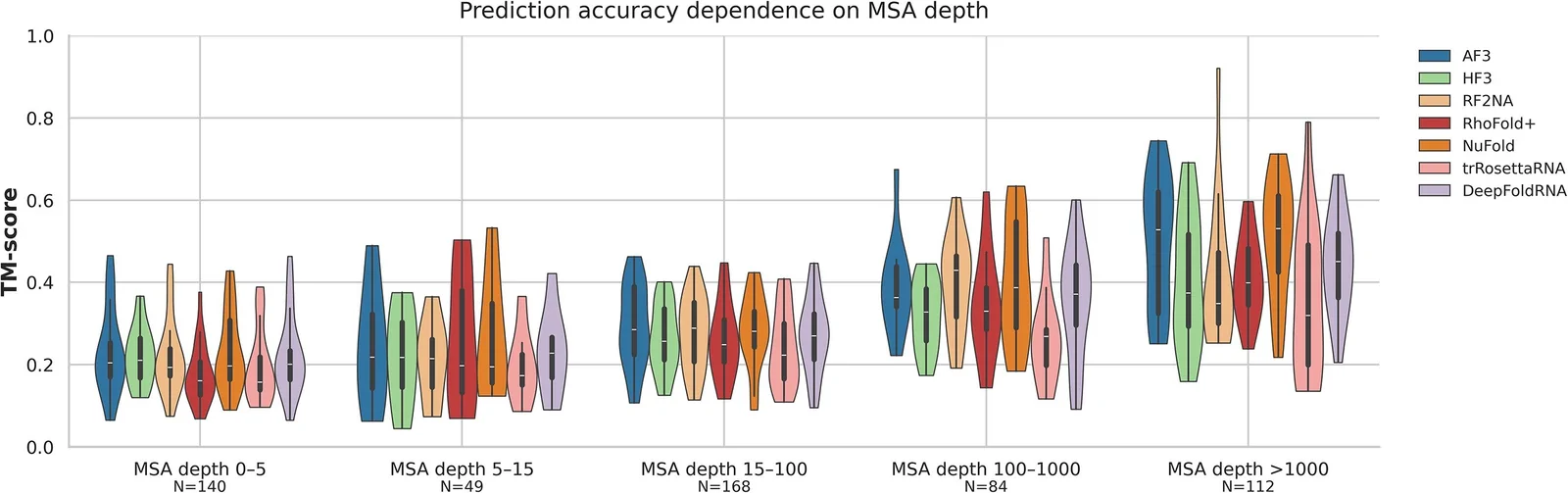
Prediction accuracy dependence on MSA depth. Distribution of TM-score across five MSA depth categories. *N* shows the total number of models in each category, across all methods. Results are shown for monomeric RNA prediction methods that incorporate MSAs during inference. Each colour corresponds to a different method

For all methods that incorporate MSA during inference, we computed MSA depth using NEFFy [29]. Across all tested methods, we observe a positive correlation between MSA depth and model quality, as measured by TM-score. The greatest improvement was observed for AF3 and NuFold (Fig. 3). However, correlations for individual targets remained moderate, suggesting that although deeper MSAs generally improve prediction accuracy, substantial gains are primarily observed for targets with very high sequence coverage. MSA metrics are all reported in Supplementary Table S8.

### Performance of structure prediction for RNA–RNA and RNA–protein complexes

To evaluate the performance of the benchmarked methods on RNA–RNA and RNA–protein complexes, we calculated the TM-score to evaluate the overall topology of the complex, and DockQ score, F1 score, fnat, iRMSD, and LRMSD to evaluate interface accuracy. We further divided this dataset into easy and hard sets based on the structural similarity of individual chains to the PDB. Complexes were put into an easy category if the protein or RNA chain had a similarity of ≥0.7 TM-score to any complex in the PDB before the cut-off date. Otherwise, complexes were part of a hard dataset. The results are shown in Tables 4 and 5.

**Table 4.** Evaluation of RNA complexes for the easy dataset, sorted by TM-score

Easy targets	DockQ	F1	iRMSD	LRMSD	fnat	TM-score
AF3	**0.417**	**0.393**	**10.468**	**22.831**	**0.844**	**0.415**
Boltz-1	0.382	0.346	**10.218**	**24.546**	**0.808**	0.387
HF3	0.173	0.108	21.120	43.494	0.448	*0.108*
RF2NA	0.075	0.033	26.885	56.081	0.594	*0.036*

The best- and worst-performing methods for each score are underlined. Methods not statistically different from the best method are shown in bold. Methods not statistically different from the worst method are shown in italic.

**Table 5.** Evaluation of RNA complexes for the hard dataset, sorted by TM-score

Hard targets	DockQ	F1	iRMSD	LRMSD	fnat	TM-score
AF3	**0.215**	**0.202**	**21.392**	**43.887**	**0.199**	**0.470**
Boltz-1	**0.224**	**0.205**	**19.637**	**41.236**	**0.216**	**0.446**
HF3	0.165	**0.162**	23.341	48.579	*0.161*	*0.288*
RF2NA	0.105	0.135	31.465	60.605	*0.137*	*0.286*

The best- and worst-performing methods for each score are underlined. Methods not statistically different from the best method are shown in bold. Methods not statistically different from the worst method are shown in italic.

Among the four methods, AF3 and Boltz-1 stand out, achieving mean TM-scores of 0.415 and 0.387, and mean DockQ scores of 0.417 and 0.382, respectively, for easy targets. The ranking of the methods was preserved for the other evaluation metrics as well (Table 4). For the hard targets (Table 5), AF3 achieved a mean TM-score of 0.470, followed by Boltz-1 (0.446); in contrast, when using DockQ, Boltz-1 was the best method (<DockQ> = 0.224), followed by AF3 with a mean DockQ of 0.215. None of the differences between AF3 and Boltz-1 was significant according to the Wilcoxon signed-rank test (Supplementary Figs S13 and S14), but RF2NA and HF3 showed significantly lower performance for both datasets. All scores are given in Supplementary Tables S9–S14.

**Figure 4.**
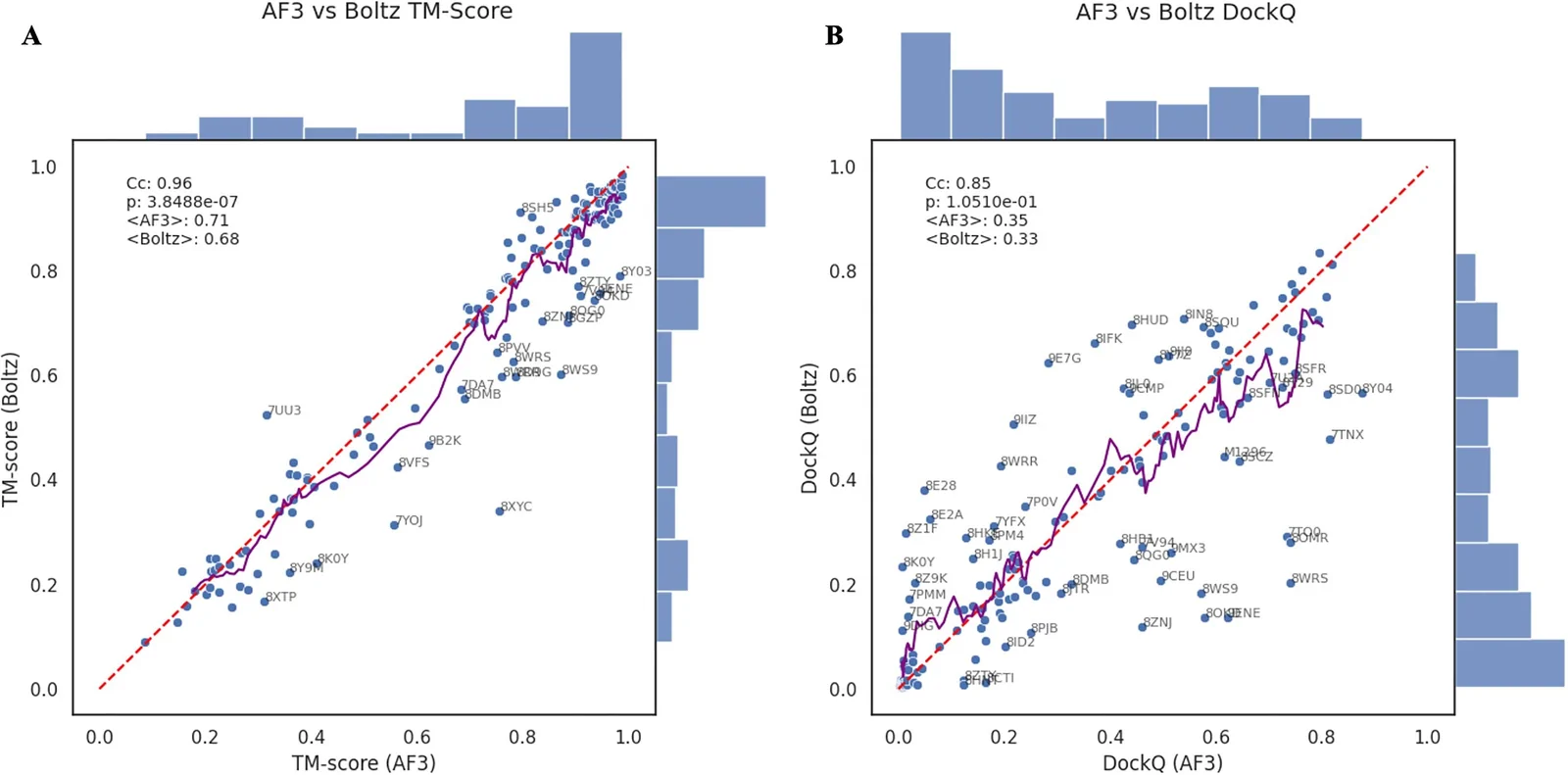
(**A**) Scatter plot showing correlation between AF3 and Boltz-1 TM-score for RNA complexes (Cc = 0.96, *P* = 3.85 × 10^−7^). A rolling average of the scores from the two methods is shown as a purple line. Bar plots on each side show the distribution of scores for each model separately (AF3 on the *x*-axis, Boltz on the *y*-axis). (**B**) Scatter plot showing correlation between AF3 and Boltz-1 DockQ score for complexes (Cc = 0.85, *P* = 1.05 × 10^−1^)

The relationships among model qualities between AF3 and Boltz-1 on a whole dataset are shown in Fig. 4A for TM-score and Fig. 4B for DockQ score. The remaining methods are shown in Supplementary Fig. S15. AF3 outperformed Boltz-1 in some cases. A statistically significant difference was found for TM-score (*P* = 3.85 × 10^−7^; Fig. 4A), but no statistically significant difference was found for DockQ score (*P* = 1.05 × 10^−1^; Fig. 4B).

To gain more insights, we analysed individual RNA–RNA and RNA–protein interfaces within each complex by calculating DockQ scores for each pair of chains, i.e. the interface DockQ (IF-DockQ). Data from the easy and hard sets were processed together in the further analysis. The individual DockQ scores for RNA–RNA interfaces, compared with those for RNA–protein interfaces, suggest no significant difference in prediction accuracy between the two in the case of AF3 (*P* = 0.07) and RF2NA (*P* = 0.13), while RNA–protein predictions are significantly less accurate than RNA–RNA predictions for both Boltz-1 (*P* = 3 × 10^−5^) and HF3 (*P* = 0.001), when calculated using Mann–Whitney U-test. AF3 shows the highest mean IF-DockQ score for RNA–protein (0.281), while Boltz-1 shows the highest mean IF-DockQ for RNA–RNA interfaces (0.420). All mean scores for individual interfaces are shown in Supplementary Table S15.

IF-DockQ scores across all methods revealed that the same models consistently achieved the highest scores. The highest IF-DockQ between an RNA and a protein chain was achieved by HF3 in 8T29 (0.670), followed closely by AF3 in 8T29 (0.647). The highest IF-DockQ between two RNA chains was achieved by Boltz-1 in 7TNY (0.894), followed by AF3 in 7TNX (0.883). Notably, these highly accurate models are either tRNA-like RNAs or double-helical RNAs in protein complexes, probably benefiting from the relative simplicity of base pair complementarity, which makes them easier to predict (Supplementary Fig. S16).

### Models with low DockQ scores and high TM-scores

**Figure 5.**
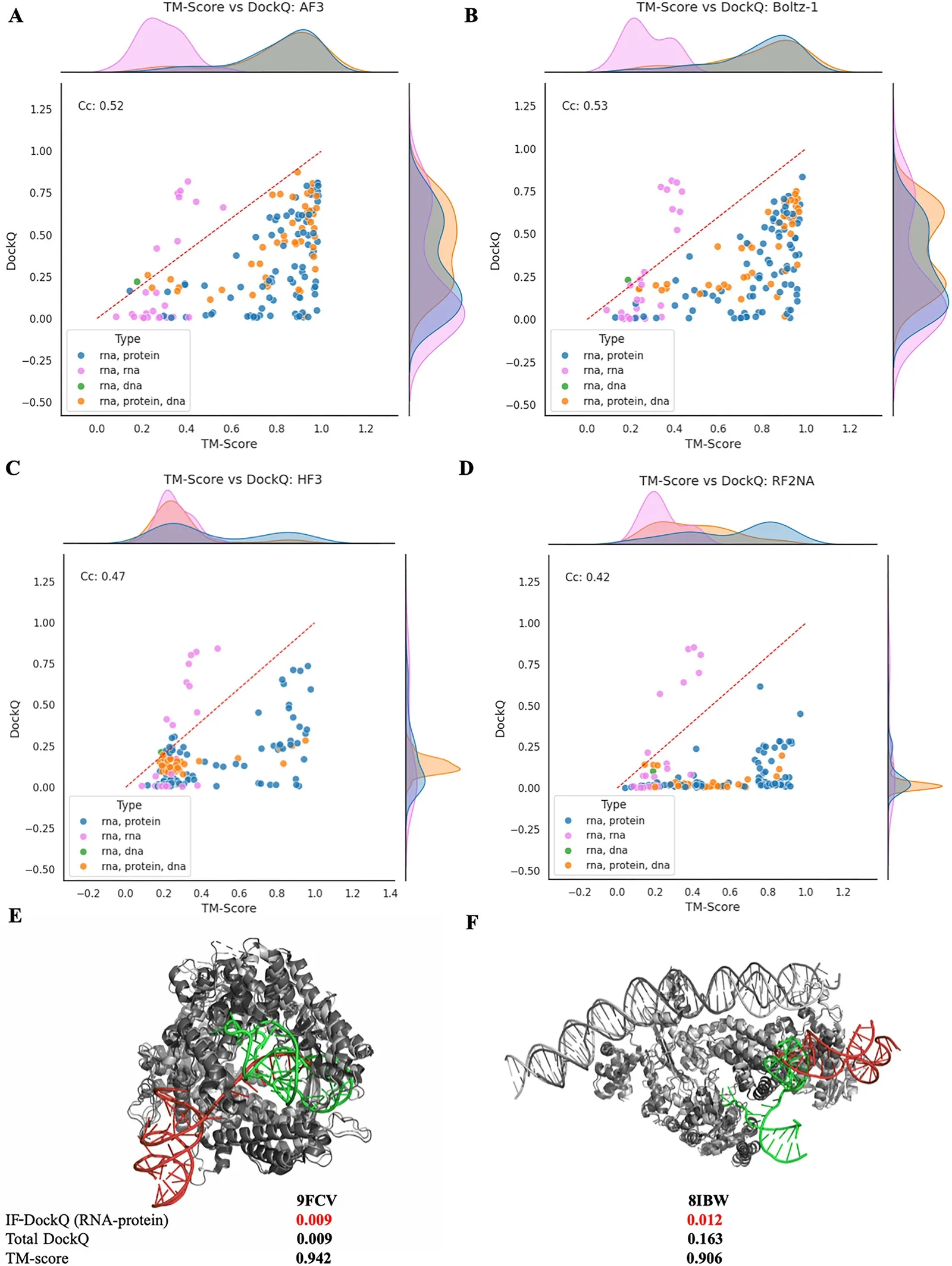
Scatter plots showing the correlation between TM-score and DockQ. Points are coloured based on the complex type (RNA–protein in blue, RNA–RNA in pink, RNA–DNA in green, and RNA–protein–DNA in orange). Plots are presented for each RNA complex prediction method: (**A**) AF3, (**B**) Boltz-1, (**C**) HF3, and (**D**) RF2NA. (**E**) Structure of 9FCV predicted with AF3. (**F**) Structure of 8IBW predicted with AF3. In both examples, the reference RNA chain is shown in green, and the predicted mislocalized RNA chain in red. TM-score is high, indicating an accurately modelled topology of complexes, whereas IF-DockQ indicates incorrectly predicted binding sites.

To investigate whether an accurate prediction of the overall complex fold also implies correct modelling of RNA–protein or RNA–RNA interfaces, we examined the relationship between interface quality and global structural accuracy. Specifically, for each method, we generated scatter plots comparing DockQ score against TM-score (Fig. 5A–D). Across many complexes, we observed substantial differences between DockQ and TM-score, revealing that these two measures capture distinct aspects of prediction quality. In addition, this also indicates that for many models, although the overall fold of the complex is often modelled correctly, the binding interface is not predicted accurately. In such cases, TM-score exceeds 0.45, while the corresponding DockQ falls below the 0.23 threshold and is therefore considered an inaccurately modelled interface. AF3 and Boltz-1 still show much higher agreement between the two metrics than HF3 and RF2NA do. Consequently, the correlation between DockQ and TM-score is weak (Fig. 5A–D).

The most pronounced example of this discrepancy is observed in the complex 9FCV, where both the protein and RNA components adopt accurate individual topologies, but the RNA is mispositioned relative to the protein and binds at an incorrect site. This resulted in a very low DockQ score and an IF-DockQ between RNA and protein of only 0.009 (Fig. 5E). The second largest discrepancy was found in a model 8IBW, in which the RNA chain is predicted to bind in the opposite orientation, resulting in a DockQ score of 0.163 and an IF-DockQ of 0.012 (Fig. 5F).

Furthermore, a few RNA–RNA complexes show high DockQ scores but very low TM-scores. These structures are short RNA–RNA complexes (< 20 nt) for which TM-score underevaluates structural accuracy, as with monomeric RNAs. In total, there were eight such structures: 7ECJ, 7ECK, 8HB1, 8YDC, 9BXE, 9CPD, 9CPJ, and 9I8B.

### Evaluation of the binding site prediction accuracy

**Figure 6.**
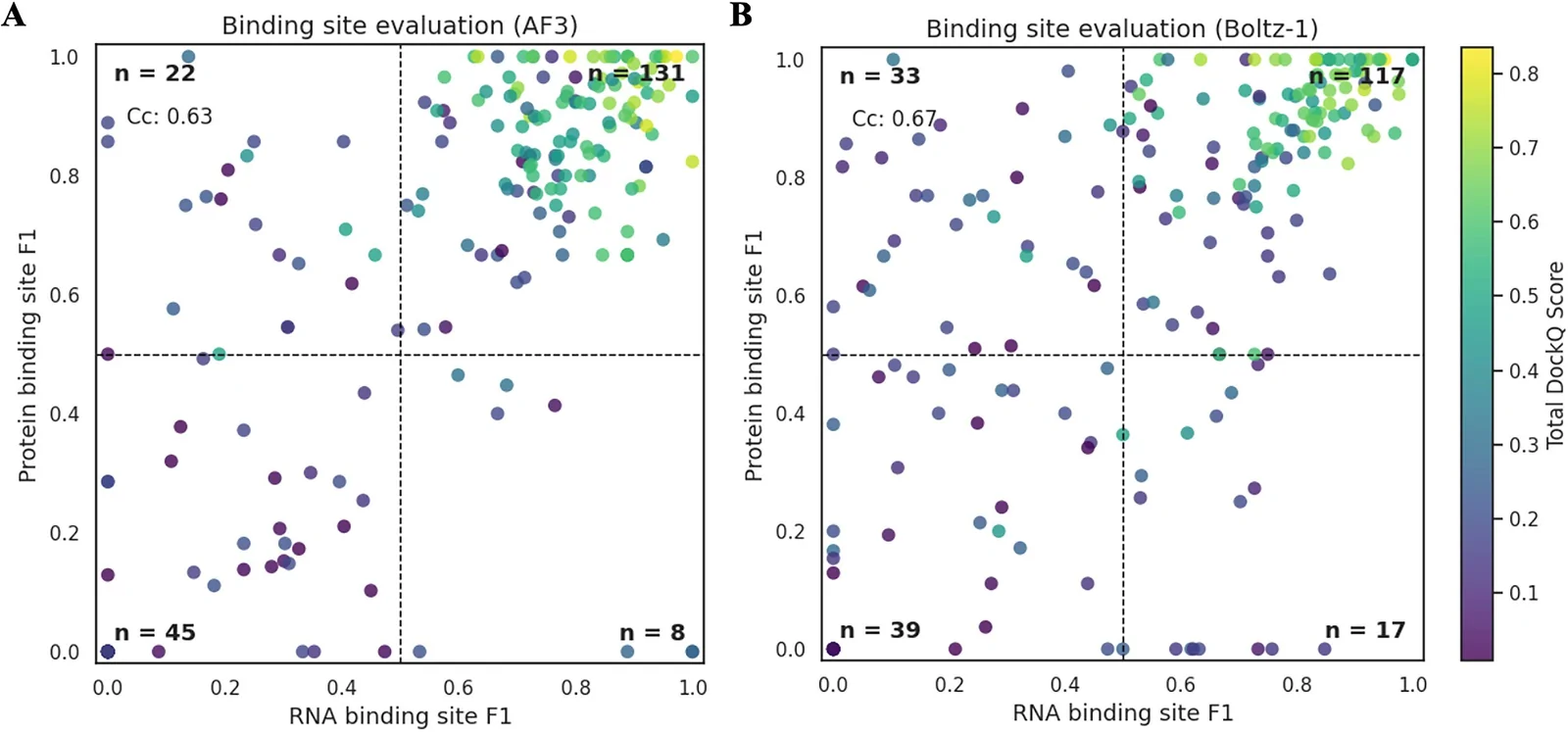
Scatter plots showing the correlation between RNA- and protein-binding site F1 score, coloured by DockQ score for each RNA–protein interface, for (**A**) AF3 and (**B**) Boltz-1. The plot is divided into four quadrants and the number of interfaces is counted and shown in bold.

Some models may have a low DockQ score even when the correct protein- or RNA-binding site is identified. To distinguish such cases, we calculated an independent binding site F1 score for the protein and RNA chains (see the Materials and methods, ‘Model evaluation’). Such cases indicate that the prediction method identifies a correct binding site but incorrectly positions one chain relative to the interacting partner. Figure 6 shows the relationship between protein- and RNA-binding site F1, coloured by the DockQ score of the structure for AF3 and Boltz-1, and Supplementary Fig. S17 for HF3 and RF2NA.

The plots in Fig. 6 can be divided into four quadrants using F1 thresholds of 0.5. Most RNA–protein complexes have a high-quality interface (DockQ > 0.23) and high protein- and RNA-binding F1 scores. However, 75 models for AF3 and 89 for Boltz-1 have one or both interfaces incorrect (low F1 scores). In most cases, both interfaces are wrong, but it is more common to have only a good protein-binding site (22/33), than vice versa (8/17).

In addition, for each RNA–protein interface, we estimated the TM-score for both the RNA and protein chains. We present the same scatter plots as in Fig. 6; however, this time coloured by TM-score for RNA chains (Supplementary Fig. S18) and protein chains (Supplementary Fig. S19). We observe that most RNA chains are predicted inaccurately, with TM-scores < 0.5, whereas most protein chains are predicted accurately, with TM-scores > 0.8. We noticed a couple of cases where Boltz-1 predicted both RNA and protein topologies accurately but still yielded low protein-binding site F1 (around 0) and a low DockQ score. As mentioned above (Fig. 5E), RNA–protein complex 9FCV had a misplaced RNA chain, resulting in a very low DockQ score of 0.009. The protein-binding site F1 for 9FCV was 0.76 (AF3), indicating that the correct RNA sequence was predicted to bind; however, this RNA bound to another RNA-binding site on the protein. RNA-binding site F1 for this model was 0.194.

## Conclusion

Advancements in deep learning have significantly impacted RNA structural biology, enabling the prediction of both monomeric RNA structures and RNA complexes. In this benchmark study, we systematically evaluated several state-of-the-art deep learning methods for RNA structure prediction, revealing their strengths and limitations. Our results suggest that while current methods show promising performance, particularly for well-characterized RNA motifs, substantial challenges remain.

Across all models, fewer than a third achieve high accuracy according to our multi-metric success criteria. These models predominantly succeed in predicting RNAs structurally similar to previously solved RNA structures, such as L-shaped RNAs, RNAs dominated by simple loop motifs, and molecules with a high fraction of paired bases, including double-helical RNAs. In contrast, longer RNAs and those with more complex folds are consistently more difficult to predict accurately. This performance gap highlights a central limitation of current RNA structure prediction methods: their reliance on training data that does not capture the full diversity of the RNA structural space, possibly due to the limited diversity of solved RNA structures deposited in the PDB and the dynamic nature of RNA. This is why those methods achieve high accuracy only on RNAs that resemble known folds but struggle to generalize to novel structures. In addition, this makes it hard for deep learning-based models to learn the underlying physics of RNA folding. Similarly, a strong dependence of predictor performance on how closely an available template matched the target was also discussed in the CASP16 evaluation [11]. Therefore, these findings underscore the need for broader, more structurally diverse RNA training datasets and improved strategies for model selection and confidence estimation.

One of the most pronounced challenges identified in this benchmark is the accurate modelling of RNA–protein interactions. In many cases, both the RNA and protein chains individually adopt the correct global fold, yet the RNA is incorrectly bound to the wrong region of the protein surface. A likely explanation for this behaviour is bias in the training data, where the method may preferentially identify protein-binding sites that are frequently involved in interactions in the PDB but are associated with different ligand or protein–protein interfaces rather than RNA. As shown, in many instances, RNA-binding sites are predicted accurately, but the RNA chain is incorrectly bound to a different protein-binding site.

RNA is increasingly recognized as a versatile macromolecule with essential catalytic and regulatory functions, many of which are still unknown. We anticipate that growing interest in RNA biology will stimulate further experimental efforts towards solving complex RNA structures. However, in the absence of an experimentally resolved structure or a closely related RNA family member with a known fold, we still recommend interpreting predicted RNA structures cautiously and, where possible, complementing them with experimental validation. Combined with innovations in techniques such as cryo-EM [2] and continued advances in machine learning [30], we are optimistic that RNA 3D structure prediction will evolve beyond simple motifs and towards accurately modelling the full complexity of RNA structural space.

## Supplementary Material

gkag813_Supplemental_File

## Data Availability

The code used for running the methods and analysing the main results is available from https://doi.org/10.5281/zenodo.21241326 and https://github.com/iammarcol/RNA-Benchmark
