# Supplementary material for: Limits of deep-learning-based RNA prediction methods
Source: Nucleic Acids Res. 2026 Aug 24;54(16):gkag813. doi: 10.1093/nar/gkag813 (PMC13501138; doi:10.1093/nar/gkag813)
Supplement: Supplementary file 1 [file gkag813_supplemental_file.pdf]

Supplementary Material for

Limits of deep-learning-based RNA prediction methods

Marko Ludaic, Arne Elofsson*

Supplementary Tables

Table S1. Deep-learning-based methods used in the benchmark and software versions.

METHOD	VERSION
AlphaFold 3	3.0.1
Boltz-1	0.4.1
Chai-1	0.6.1
DeepFoldRNA	1.0
DRfold	commit pre-release-4-g9586990
HelixFold3	HelixFold3/73cd80b-hpc1
NuFold	commit 169a618
RhoFold+	0.0.1
RosettaFoldNA	commit f761af2
trRosettaRNA	1.0

Table S2. TM-score for the whole benchmarked dataset of monomeric RNA molecules across all tested deep learning methods for monomeric RNA prediction, for both easy and hard dataset.

PDB (Easy)	AF3	Boltz-1	Chai-1	HF3	NuFold	RhoFold+	RoseTTAFoldNA	trRosettaRNA	DRfold	DeepFoldRNA
7KUB	0.36302	0.39686	0.33353	0.35902	0.4023	0.33309	0.4335	0.38691	0.43084	0.39256
7KUC	0.46535	0.33929	0.33573	0.36668	0.41476	0.26203	0.40323	0.36668	0.52201	0.40595
7KUD	0.38398	0.27041	0.30498	0.35551	0.34221	0.12448	0.20897	0.38897	0.42733	0.35017
7RWR	0.38245	0.44787	0.27836	0.28942	0.37568	0.39044	0.36085	0.39621	0.43429	0.44675
7UME	0.39126	0.36121	0.40673	0.22378	0.38316	0.27976	0.30098	0.14482	0.40486	0.27821
7V06	0.31867	0.389	0.48251	0.31508	0.35875	0.3823	0.31852	0.30012	0.4028	0.44887
7WIA	0.38924	0.3466	0.32442	0.26068	0.42405	0.30375	0.39318	0.38543	0.4363	0.37515
8CQ1	0.45643	0.42347	0.38626	0.38878	0.41691	0.36146	0.43917	0.40835	0.35597	0.4203
8FB3	0.36314	0.37594	0.25761	0.26562	0.58616	0.46508	0.30276	0.2659	0.35065	0.16767
8FCS	0.67471	0.615	0.59979	0.43196	0.63414	0.62008	0.60651	0.50856	0.70587	0.60069
8K2Z	0.55552	0.58768	0.34971	0.29386	0.44706	0.35743	0.27612	0.13557	0.3715	0.39662
8K30	0.61317	0.37553	0.53594	0.3084	0.63489	0.40124	0.33291	0.53919	0.44504	0.45235
8KEB	0.66235	0.60309	0.31577	0.69134	0.71253	0.59691	0.27968	0.1606	0.60011	0.63647
8Q6N	0.33247	0.54981	0.40027	0.44513	0.54175	0.3297	0.34414	0.12914	0.32963	0.46245
8SCF	0.45615	0.41437	0.35514	0.39699	0.41473	0.29352	0.45921	0.11683	0.38527	0.47586
8UPT	0.62407	0.66113	0.40738	0.21266	0.6028	0.46457	0.38416	0.49638	0.55814	0.55651
8UYP	0.43726	0.31299	0.22662	0.38017	0.37182	0.32979	0.42463	0.20298	0.35185	0.37341
8V1H	0.57092	0.59434	0.56451	0.58333	0.55012	0.49749	0.46147	0.13601	0.55377	0.51151
8V1I	0.61837	0.56803	0.42743	0.29223	0.5797	0.40787	0.37802	0.48961	0.51376	0.48587
8VT5	0.68961	0.67695	0.46968	0.40826	0.52719	0.48161	0.5076	0.54258	0.50472	0.54364
8XZL	0.27199	0.40012	0.30423	0.2601	0.27101	0.25009	0.36679	0.20187	0.2762	0.30814
9BLM	0.44052	0.51669	0.37959	0.23524	0.56686	0.47366	0.44748	0.28217	0.42566	0.43615
9DE6	0.43479	0.26276	0.55175	0.53952	0.53482	0.35804	0.59007	0.43245	0.56904	0.37101
9DE7	0.50035	0.44777	0.48962	0.50675	0.49865	0.39677	0.30042	0.47788	0.54552	0.40661
9ECQ	0.35831	0.50386	0.37724	0.27222	0.42793	0.24676	0.35474	0.35127	0.34187	0.33686
9ECR	0.41407	0.37967	0.4179	0.29519	0.3495	0.27964	0.44445	0.31863	0.32494	0.46338
R1211	0.47669	0.41407	0.41496	0.50952	0.45052	0.30872	0.34751	0.33897	0.34454	0.32637
R1263	0.304	0.49274	0.27553	0.48693	0.66333	0.48397	0.92072	0.7898	0.73992	0.49703
R1271	0.74432	0.72539	0.62939	0.66229	0.66617	0.57549	0.61516	0.1657	0.71862	0.66176
R1293	0.35571	0.36727	0.45625	0.40129	0.31029	0.23932	0.24329	0.38649	0.34689	0.2656

PDB (Hard)	AF3	Boltz-1	Chai-1	HF3	NuFold	RhoFold+	RoseTTAFoldNA	trRosettaRNA	DRfold	DeepFoldRNA
**7MKT**	0.06505	0.10619	0.11349	0.12008	0.08987	0.06848	0.07464	0.09654	0.11502	0.06492
**7PS8**	0.13037	0.10516	0.14895	0.13334	0.09658	0.1169	0.13584	0.12639	0.1126	0.09548
**7Q48**	0.10714	0.12714	0.12852	0.13898	0.09031	0.11763	0.13405	0.13234	0.09373	0.13903
**7Q6L**	0.10097	0.14818	0.12497	0.14837	0.11873	0.12734	0.14177	0.15612	0.13568	0.12973
**7QA2**	0.14075	0.15234	0.11873	0.12574	0.12327	0.1641	0.11432	0.10928	0.1256	0.11384
**7SHX**	0.17544	0.24919	0.17696	0.26902	0.17875	0.19728	0.19857	0.19667	0.25753	0.22776
**7SXP**	0.06271	0.23686	0.13791	0.0447	0.12365	0.06944	0.09124	0.17309	0.07753	0.13076
**7UMC**	0.34501	0.41391	0.31398	0.29232	0.31915	0.36128	0.4994	0.29601	0.46802	0.33267
**7UMD**	0.29226	0.29686	0.34879	0.21713	0.31574	0.3279	0.2611	0.16665	0.30402	0.2637
**8BTZ**	0.28753	0.22254	0.54226	0.25987	0.3149	0.3763	0.26744	0.21728	0.28162	0.23699
**8BWT**	0.22597	0.21981	0.24246	0.21498	0.20018	0.22829	0.21143	0.17153	0.21789	0.19714
**8CLR**	0.24165	0.22713	0.25075	0.27882	0.30522	0.23224	0.28221	0.20346	0.22188	0.22519
**8FZA**	0.26184	0.19618	0.18728	0.21402	0.25951	0.17132	0.24406	0.27127	0.27094	0.18634
**8HB8**	0.22502	0.19319	0.29295	0.19791	0.27355	0.21816	0.22435	0.14942	0.24477	0.27167
**8I43**	0.19923	0.19486	0.16197	0.26151	0.20185	0.15882	0.22929	0.14755	0.22297	0.213
**8I44**	0.21506	0.21954	0.17371	0.15447	0.20255	0.15596	0.16523	0.13993	0.17729	0.18189
**8I45**	0.20611	0.19284	0.16642	0.20113	0.18629	0.15649	0.21105	0.13365	0.18048	0.16469
**8I46**	0.19182	0.21902	0.18004	0.24284	0.2308	0.19841	0.18263	0.14397	0.22853	0.14745
**8ITS**	0.3098	0.26224	0.28633	0.29085	0.25766	0.21876	0.30097	0.17217	0.27375	0.2541
**8JHP**	0.21709	0.25444	0.24458	0.22424	0.24537	0.16361	0.17925	0.1402	0.14737	0.1792
**8K8B**	0.23959	0.20711	0.17385	0.20575	0.16605	0.16353	0.18501	0.18352	0.20082	0.20221
**8Q4O**	0.13807	0.11463	0.10855	0.13689	0.14232	0.10461	0.12586	0.10659	0.10659	0.16128
**8QO2**	0.46272	0.24538	0.22837	0.20328	0.31543	0.44752	0.27924	0.28936	0.2674	0.29891
**8QO3**	0.48945	0.41034	0.39579	0.37517	0.5326	0.43298	0.3651	0.36604	0.43417	0.42177
**8QO4**	0.40196	0.41335	0.27552	0.33193	0.38195	0.43898	0.36692	0.29529	0.43362	0.39941
**8QO5**	0.42299	0.31311	0.23883	0.36726	0.29979	0.26341	0.48055	0.21046	0.38737	0.36905
**8SCH**	0.34403	0.29319	0.3242	0.29693	0.23178	0.3039	0.32097	0.2726	0.35031	0.33954
**8T5O**	0.25107	0.30955	0.2834	0.15921	0.21788	0.3091	0.25267	0.21043	0.23016	0.20535
**8TNS**	0.1101	0.14566	0.10459	0.10507	0.13141	0.09314	0.07363	0.08636	0.02871	0.09038
**8UTG**	0.15968	0.16531	0.05151	0.12801	0.14738	0.09691	0.13584	0.13591	0.09463	0.13528
**8UYG**	0.39278	0.36566	0.30983	0.30681	0.29237	0.34361	0.33127	0.22807	0.28274	0.35105
**8UYM**	0.39602	0.40098	0.27704	0.38417	0.27121	0.32686	0.39409	0.31631	0.39224	0.31955
**8VCI**	0.27375	0.36171	0.23514	0.35249	0.28861	0.27583	0.29306	0.32766	0.31569	0.26676
**8ZAU**	0.35335	0.29659	0.3289	0.33727	0.3821	0.5033	0.26212	0.25314	0.28463	0.26946

**9BUN**	0.21178	0.38283	0.37039	0.36321	0.32323	0.29476	0.286	0.28951	0.35002	0.33548
**9EOW**	0.21778	0.20014	0.16285	0.18449	0.1944	0.17013	0.21459	0.13366	0.1998	0.2059
**9EWW**	0.2224	0.26513	0.1944	0.17392	0.18445	0.14388	0.1918	0.18433	0.20739	0.09177
**9FM4**	0.24104	0.2416	0.20345	0.2167	0.21894	0.16578	0.20931	0.23808	0.23361	0.18375
**9FO8**	0.22161	0.23082	0.1992	0.17442	0.23475	0.17154	0.19325	0.19494	0.18156	0.21732
**9FO9**	0.26401	0.24704	0.2359	0.25152	0.25481	0.21836	0.20034	0.21747	0.20999	0.27688
**9IO0**	0.20246	0.24801	0.18491	0.19481	0.19119	0.16545	0.17503	0.22239	0.23529	0.17206
**9IO1**	0.14843	0.21158	0.14867	0.20285	0.15892	0.16813	0.19405	0.20374	0.20414	0.19116
**9IOR**	0.23935	0.2393	0.24999	0.22025	0.23692	0.16596	0.21245	0.14246	0.2733	0.23093
**9IOS**	0.18702	0.22048	0.19625	0.1721	0.18025	0.12374	0.19107	0.11185	0.2141	0.19988
**9IOU**	0.17398	0.17086	0.20248	0.21529	0.18137	0.15022	0.19258	0.15908	0.18798	0.23294
**R1205**	0.30023	0.25182	0.269	0.34961	0.32268	0.24638	0.34767	0.25095	0.28081	0.24197
**R1209**	0.3027	0.30327	0.25469	0.23275	0.27681	0.23844	0.3004	0.25086	0.30331	0.28053
**R1288**	0.29578	0.30832	0.24632	0.2532	0.34882	0.25044	0.29095	0.18367	0.31149	0.26842
**R1296**	0.32625	0.29787	0.26851	0.33956	0.3388	0.29936	0.35016	0.2846	0.26721	0.33973

Table S3. INF (INF-ALL) for the whole benchmarked dataset of monomeric RNA molecules across all tested deep learning methods for monomeric RNA prediction, for both easy and hard dataset.

PDB (Easy)	AF3	Boltz-1	Chai-1	HF3	NuFold	RhoFold+	RoseTTAFoldNA	trRosettaRNA	DRfold	DeepFoldRNA
7KUB	0.88	0.81	0.86	0.85	0.78	0.64	0.69	0.63	0.82	0.82
7KUC	0.97	0.95	0.86	0.95	0.92	0.82	0.97	0.92	0.88	0.88
7KUD	0.82	0.76	0.9	0.8	0.74	0.85	0.77	0.77	0.89	0.89
7RWR	0.67	0.68	0.69	0.62	0.74	0.69	0.69	0.75	0.71	0.71
7UME	0.82	0.81	0.77	0.8	0.83	0.78	0.76	0.47	0.8	0.8
7V06	0.72	0.74	0.83	0.8	0.62	0.72	0.73	0.49	0.71	0.71
7WIA	0.82	0.79	0.8	0.73	0.81	0.7	0.68	0.84	0.79	0.79
8CQ1	0.9	0.9	0.87	0.88	0.84	0.72	0.81	0.62	0.82	0.82
8FB3	0.71	0.68	0.46	0.73	0.78	0.66	0.62	0.55	0.55	0.55
8FCS	0.88	0.85	0.88	0.88	0.88	0.78	0.91	0.8	0.86	0.86
8K2Z	0.9	0.63	0.76	0.86	0.84	0.79	0.66	0.2	0.8	0.8
8K30	0.4	0.24	0.33	0.35	0.38	0.38	0.33	0.3	0.37	0.37
8KEB	0.37	0.37	0.39	0.37	0.34	0.31	0.38	0.28	0.33	0.33
8Q6N	0.84	0.87	0.76	0.83	0.85	0.69	0.75	0.33	0.77	0.77
8SCF	0.82	0.82	0.81	0.8	0.81	0.79	0.76	0.3	0.82	0.82
8UPT	0.77	0.74	0.69	0.71	0.72	0.68	0.63	0.57	0.74	0.74
8UYP	0.89	0.74	0.77	0.83	0.72	0.75	0.72	0.59	0.81	0.81
8V1H	0.35	0.33	0.33	0.34	0.37	0.33	0.24	0.32	0.42	0.42
8V1I	0.36	0.33	0.4	0.33	0.32	0.35	0.32	0.24	0.39	0.39
8VT5	0.87	0.87	0.76	0.88	0.82	0.81	0.74	0.58	0.83	0.83
8XZL	0.82	0.83	0.79	0.71	0.75	0.73	0.66	0.32	0.72	0.72
9BLM	0.83	0.89	0.62	0.81	0.86	0.68	0.8	0.43	0.75	0.75
9DE6	0.89	0.38	0.88	0.92	0.88	0.87	0.75	0.69	0.9	0.9
9DE7	0.93	0.88	0.78	0.88	0.86	0.84	0.77	0.56	0.84	0.84
9ECQ	0.98	0.92	0.8	0.92	0.87	0.78	0.89	0.78	0.92	0.92
9ECR	0.86	0.73	0.74	0.85	0.82	0.67	0.75	0.56	0.79	0.79
R1211	0.88	0.88	0.86	0.89	0.84	0.77	0.76	0.61	0.76	0.76
R1263	0.86	0.92	0.75	0.84	0.86	0.86	0.96	0.78	0.89	0.89
R1271	0.82	0.82	0.79	0.82	0.81	0.72	0.79	0.36	0.8	0.8
R1293	0.68	0.76	0.71	0.76	0.69	0.6	0.66	0.5	0.61	0.61

PDB (Hard)	AF3	Boltz-1	Chai-1	HF3	NuFold	RhoFold+	RoseTTAFoldNA	trRosettaRNA	DRfold	DeepFoldRNA
7MKT	0.0	0.0	0.0	0.0	0.0	0.0	0.0	0.0	0.0	0.0
7PS8	0.25	0.31	0.35	0.21	0.26	0.23	0.33	0.26	0.17	0.17
7Q48	0.33	0.45	0.49	0.34	0.33	0.37	0.38	0.36	0.48	0.48
7Q6L	0.31	0.36	0.36	0.32	0.3	0.36	0.45	0.28	0.41	0.41
7QA2	0.4	0.49	0.46	0.46	0.27	0.45	0.42	0.36	0.41	0.41
7SHX	0.59	0.7	0.62	0.65	0.68	0.62	0.64	0.47	0.6	0.6
7SXP	0.3	0.43	0.33	0.24	0.25	0.44	0.34	0.36	0.32	0.32
7UMC	0.82	0.83	0.7	0.83	0.73	0.78	0.75	0.64	0.84	0.84
7UMD	0.81	0.82	0.81	0.71	0.68	0.75	0.78	0.55	0.77	0.77
8BTZ	0.88	0.87	0.89	0.9	0.79	0.77	0.62	0.45	0.7	0.7
8BWT	0.91	0.89	0.89	0.92	0.83	0.82	0.87	0.79	0.8	0.8
8CLR	0.97	0.97	0.91	0.88	0.94	0.94	0.83	0.76	0.63	0.63
8FZA	0.67	0.37	0.62	0.69	0.65	0.48	0.48	0.41	0.57	0.57
8HB8	0.61	0.59	0.56	0.5	0.53	0.42	0.48	0.2	0.51	0.51
8I43	0.81	0.78	0.81	0.85	0.69	0.74	0.67	0.69	0.71	0.71
8I44	0.72	0.81	0.74	0.86	0.69	0.71	0.76	0.22	0.69	0.69
8I45	0.89	0.83	0.8	0.87	0.72	0.84	0.76	0.32	0.79	0.79
8I46	0.9	0.9	0.85	0.94	0.74	0.82	0.65	0.72	0.84	0.84
8ITS	0.84	0.85	0.84	0.87	0.88	0.81	0.82	0.45	0.85	0.85
8JHP	0.63	0.65	0.77	0.72	0.78	0.64	0.55	0.47	0.73	0.73
8K8B	0.66	0.78	0.71	0.74	0.66	0.72	0.72	0.34	0.7	0.7
8Q4O	0.25	0.31	0.34	0.3	0.23	0.15	0.29	0.29	0.32	0.32
8QO2	0.57	0.55	0.55	0.58	0.66	0.52	0.52	0.58	0.24	0.24
8QO3	0.75	0.76	0.76	0.78	0.8	0.74	0.71	0.56	0.69	0.69
8QO4	0.7	0.67	0.67	0.68	0.71	0.66	0.68	0.62	0.66	0.66
8QO5	0.69	0.64	0.62	0.64	0.68	0.59	0.6	0.56	0.64	0.64
8SCH	0.82	0.83	0.72	0.78	0.76	0.78	0.85	0.65	0.86	0.86
8T5O	0.41	0.41	0.44	0.39	0.47	0.27	0.4	0.31	0.39	0.39
8TNS	0.0	0.0	0.0	0.0	0.0	0.0	0.0	0.0	0.0	0.0
8UTG	0.26	0.43	0.35	0.07	0.31	0.3	0.25	0.37	0.28	0.28
8UYG	0.86	0.79	0.57	0.77	0.76	0.66	0.69	0.54	0.65	0.65
8UYM	0.84	0.78	0.78	0.79	0.79	0.74	0.72	0.7	0.73	0.73
8VCI	0.83	0.81	0.8	0.76	0.69	0.73	0.71	0.6	0.8	0.8
8ZAU	0.72	0.74	0.75	0.7	0.69	0.74	0.64	0.57	0.63	0.63
9BUN	0.74	0.76	0.67	0.71	0.69	0.61	0.69	0.47	0.69	0.69

**9EOW**	0.8	0.85	0.84	0.87	0.78	0.75	0.77	0.35	0.82	0.82
**9EWW**	0.74	0.68	0.47	0.64	0.64	0.26	0.49	0.53	0.61	0.61
**9FM4**	0.79	0.78	0.73	0.82	0.8	0.8	0.8	0.56	0.7	0.7
**9FO8**	0.77	0.76	0.74	0.82	0.79	0.8	0.7	0.61	0.78	0.78
**9FO9**	0.77	0.76	0.8	0.78	0.77	0.75	0.67	0.74	0.76	0.76
**9IO0**	0.92	0.9	0.83	0.95	0.74	0.79	0.72	0.81	0.87	0.87
**9IO1**	0.74	0.74	0.8	0.78	0.74	0.74	0.72	0.48	0.72	0.72
**9IOR**	0.82	0.82	0.76	0.78	0.59	0.73	0.71	0.66	0.78	0.78
**9IOS**	0.93	0.87	0.9	0.9	0.79	0.65	0.68	0.57	0.82	0.82
**9IOU**	0.85	0.8	0.76	0.84	0.65	0.79	0.66	0.69	0.73	0.73
**R1205**	0.6	0.57	0.44	0.59	0.57	0.46	0.58	0.48	0.32	0.32
**R1209**	0.78	0.8	0.76	0.79	0.76	0.76	0.79	0.72	0.76	0.76
**R1288**	0.68	0.68	0.64	0.6	0.65	0.65	0.64	0.38	0.66	0.66
**R1296**	0.86	0.83	0.83	0.86	0.84	0.74	0.82	0.62	0.82	0.82

Table S4. GDT-TS for the whole benchmarked dataset of monomeric RNA molecules across all tested deep learning methods for monomeric RNA prediction, for both easy and hard dataset.

PDB (Easy)	AF3	Boltz-1	Chai-1	HF3	NuFold	RhoFold+	RoseTTAFoldNA	trRosettaRNA	DRfold	DeepFoldRNA
7KUB	0.22	0.24	0.23	0.23	0.25	0.23	0.25	0.2	0.61	0.61
7KUC	0.88	0.86	0.88	0.84	0.94	0.88	0.91	0.92	0.95	0.95
7KUD	0.86	0.83	0.81	0.94	0.9	0.79	0.81	0.58	0.86	0.86
7RWR	0.64	0.7	0.57	0.48	0.64	0.62	0.6	0.66	0.66	0.66
7UME	0.82	0.78	0.8	0.64	0.79	0.74	0.8	0.54	0.76	0.76
7V06	0.57	0.61	0.78	0.64	0.59	0.64	0.62	0.46	0.69	0.69
7WIA	0.24	0.24	0.25	0.19	0.26	0.24	0.25	0.22	0.34	0.34
8CQ1	0.7	0.71	0.68	0.69	0.69	0.64	0.7	0.67	0.65	0.65
8FB3	0.71	0.76	0.26	0.57	0.92	0.86	0.53	0.68	0.72	0.72
8FCS	0.74	0.69	0.66	0.49	0.72	0.69	0.69	0.62	0.75	0.75
8K2Z	0.71	0.75	0.51	0.43	0.64	0.56	0.42	0.23	0.54	0.54
8K30	0.8	0.51	0.69	0.38	0.79	0.65	0.45	0.72	0.34	0.34
8KEB	0.76	0.69	0.37	0.77	0.79	0.67	0.33	0.22	0.34	0.34
8Q6N	0.57	0.78	0.59	0.66	0.8	0.5	0.59	0.27	0.55	0.55
8SCF	0.82	0.82	0.78	0.82	0.78	0.75	0.81	0.3	0.81	0.81
8UPT	0.16	0.16	0.16	0.15	0.14	0.13	0.14	0.14	0.64	0.64
8UYP	0.35	0.25	0.2	0.31	0.28	0.26	0.33	0.15	0.29	0.29
8V1H	0.19	0.18	0.19	0.18	0.2	0.18	0.18	0.12	0.25	0.25
8V1I	0.78	0.75	0.55	0.35	0.73	0.59	0.51	0.64	0.24	0.24
8VT5	0.8	0.8	0.59	0.52	0.72	0.64	0.63	0.67	0.39	0.39
8XZL	0.39	0.59	0.42	0.38	0.43	0.43	0.49	0.32	0.27	0.27
9BLM	0.55	0.62	0.33	0.31	0.7	0.55	0.52	0.31	0.42	0.42
9DE6	0.33	0.34	0.45	0.47	0.43	0.36	0.52	0.4	0.48	0.48
9DE7	0.46	0.46	0.43	0.45	0.45	0.38	0.31	0.43	0.46	0.46
9ECQ	0.87	0.9	0.85	0.78	0.85	0.76	0.82	0.82	0.84	0.84
9ECR	0.64	0.67	0.67	0.59	0.64	0.69	0.66	0.64	0.67	0.67
R1211	0.48	0.44	0.41	0.48	0.44	0.29	0.36	0.33	0.35	0.35
R1263	0.41	0.62	0.38	0.56	0.78	0.58	0.98	0.86	0.83	0.83
R1271	0.76	0.76	0.69	0.7	0.7	0.62	0.7	0.22	0.75	0.75
R1293	0.33	0.44	0.42	0.37	0.31	0.24	0.23	0.42	0.33	0.33

PDB (Hard)	AF3	Boltz-1	Chai-1	HF3	NuFold	RhoFold+	RoseTTAFoldNA	trRosettaRNA	DRfold	DeepFoldRNA
7MKT	0.3	0.29	0.26	0.33	0.26	0.25	0.22	0.25	0.25	0.25
7PS8	0.32	0.45	0.28	0.32	0.29	0.29	0.3	0.3	0.33	0.33
7Q48	0.31	0.38	0.32	0.3	0.31	0.32	0.36	0.34	0.33	0.33
7Q6L	0.32	0.39	0.32	0.35	0.31	0.32	0.33	0.32	0.32	0.32
7QA2	0.34	0.43	0.33	0.34	0.31	0.33	0.33	0.34	0.34	0.34
7SHX	0.2	0.21	0.2	0.2	0.19	0.19	0.19	0.16	0.2	0.2
7SXP	0.37	0.54	0.38	0.47	0.34	0.36	0.34	0.32	0.34	0.34
7UMC	0.4	0.51	0.36	0.34	0.39	0.45	0.43	0.34	0.47	0.47
7UMD	0.48	0.57	0.59	0.38	0.49	0.55	0.51	0.42	0.49	0.49
8BTZ	0.17	0.13	0.34	0.19	0.21	0.22	0.16	0.12	0.18	0.18
8BWT	0.66	0.65	0.69	0.67	0.6	0.66	0.62	0.53	0.61	0.61
8CLR	0.91	0.88	0.88	0.86	0.91	0.88	0.86	0.77	0.52	0.52
8FZA	0.75	0.46	0.47	0.73	0.73	0.65	0.45	0.76	0.74	0.74
8HB8	0.35	0.24	0.4	0.28	0.34	0.33	0.28	0.2	0.31	0.31
8I43	0.58	0.6	0.6	0.67	0.59	0.58	0.62	0.58	0.6	0.6
8I44	0.67	0.67	0.63	0.7	0.66	0.62	0.64	0.38	0.66	0.66
8I45	0.76	0.71	0.7	0.71	0.7	0.67	0.72	0.42	0.72	0.72
8I46	0.79	0.71	0.68	0.75	0.76	0.62	0.7	0.72	0.74	0.74
8ITS	0.4	0.35	0.41	0.38	0.39	0.4	0.4	0.22	0.39	0.39
8JHP	0.56	0.61	0.69	0.49	0.57	0.56	0.59	0.58	0.62	0.62
8K8B	0.63	0.67	0.57	0.62	0.6	0.58	0.62	0.4	0.79	0.79
8Q4O	0.34	0.32	0.3	0.32	0.27	0.3	0.3	0.33	0.3	0.3
8QO2	0.07	0.07	0.06	0.06	0.07	0.07	0.07	0.07	0.13	0.13
8QO3	0.37	0.35	0.35	0.34	0.39	0.38	0.28	0.28	0.38	0.38
8QO4	0.31	0.26	0.2	0.22	0.28	0.31	0.28	0.2	0.3	0.3
8QO5	0.33	0.21	0.19	0.26	0.19	0.21	0.32	0.17	0.29	0.29
8SCH	0.42	0.39	0.38	0.28	0.35	0.38	0.41	0.35	0.42	0.42
8T5O	0.19	0.26	0.19	0.15	0.21	0.2	0.19	0.16	0.1	0.1
8TNS	0.31	0.29	0.34	0.28	0.3	0.24	0.21	0.26	0.2	0.2
8UTG	0.33	0.48	0.34	0.39	0.34	0.33	0.32	0.3	0.32	0.32
8UYG	0.29	0.31	0.13	0.23	0.25	0.25	0.28	0.2	0.26	0.26
8UYM	0.36	0.33	0.24	0.33	0.23	0.26	0.28	0.25	0.29	0.29
8VCI	0.23	0.28	0.22	0.29	0.22	0.25	0.21	0.28	0.23	0.23
8ZAU	0.39	0.34	0.34	0.36	0.38	0.6	0.3	0.23	0.35	0.35

**9BUN**	0.36	0.55	0.54	0.5	0.46	0.43	0.45	0.45	0.52	0.52
**9EOW**	0.33	0.32	0.3	0.32	0.32	0.3	0.33	0.26	0.64	0.64
**9EWW**	0.2	0.2	0.09	0.16	0.17	0.13	0.18	0.16	0.17	0.17
**9FM4**	0.69	0.68	0.63	0.66	0.66	0.62	0.64	0.67	0.66	0.66
**9FO8**	0.6	0.62	0.53	0.58	0.62	0.59	0.61	0.62	0.6	0.6
**9FO9**	0.54	0.55	0.52	0.58	0.6	0.57	0.55	0.64	0.58	0.58
**9IO0**	0.54	0.54	0.55	0.53	0.54	0.58	0.53	0.54	0.71	0.71
**9IO1**	0.59	0.53	0.51	0.58	0.55	0.47	0.57	0.55	0.63	0.63
**9IOR**	0.59	0.53	0.57	0.57	0.57	0.51	0.59	0.54	0.74	0.74
**9IOS**	0.5	0.51	0.49	0.46	0.51	0.42	0.53	0.5	0.62	0.62
**9IOU**	0.49	0.51	0.47	0.49	0.51	0.41	0.5	0.49	0.59	0.59
**R1205**	0.4	0.38	0.32	0.43	0.36	0.31	0.4	0.31	0.27	0.27
**R1209**	0.33	0.33	0.29	0.31	0.3	0.3	0.32	0.3	0.33	0.33
**R1288**	0.48	0.49	0.4	0.28	0.47	0.49	0.5	0.21	0.45	0.45
**R1296**	0.38	0.35	0.31	0.39	0.37	0.3	0.39	0.31	0.32	0.32

Table S5. lDDT for the whole benchmarked dataset of monomeric RNA molecules across all tested deep learning methods for monomeric RNA prediction, for both easy and hard dataset.

PDB (Easy)	AF3	Boltz-1	Chai-1	HF3	NuFold	RhoFold+	RoseTTAFoldNA	trRosettaRNA	DRfold	DeepFoldRNA
7KUB	0.71	0.68	0.7	0.65	0.62	0.52	0.48	0.54	0.64	0.64
7KUC	0.78	0.64	0.75	0.58	0.8	0.61	0.61	0.73	0.7	0.7
7KUD	0.42	0.69	0.74	0.12	0.18	0.7	0.26	0.47	0.75	0.75
7RWR	0.54	0.54	0.52	0.43	0.48	0.52	0.44	0.57	0.43	0.43
7UME	0.67	0.67	0.66	0.59	0.6	0.63	0.3	0.5	0.04	0.04
7V06	0.68	0.75	0.79	0.73	0.54	0.67	0.52	0.49	0.62	0.62
7WIA	0.75	0.73	0.64	0.55	0.6	0.6	0.51	0.69	0.57	0.57
8CQ1	0.77	0.73	0.75	0.75	0.75	0.67	0.58	0.68	0.22	0.22
8FB3	0.63	0.42	0.25	0.29	0.78	0.69	0.1	0.48	0.0	0.0
8FCS	0.83	0.8	0.81	0.73	0.8	0.68	0.74	0.73	0.8	0.8
8K2Z	0.68	0.4	0.56	0.63	0.43	0.61	0.16	0.49	0.54	0.54
8K30	0.72	0.4	0.59	0.6	0.54	0.65	0.25	0.68	0.44	0.44
8KEB	0.79	0.56	0.54	0.74	0.53	0.64	0.25	0.49	0.42	0.42
8Q6N	0.65	0.64	0.62	0.57	0.77	0.58	0.47	0.54	0.39	0.39
8SCF	0.74	0.78	0.77	0.66	0.75	0.68	0.18	0.51	0.72	0.72
8UPT	0.68	0.69	0.6	0.55	0.54	0.6	0.4	0.63	0.54	0.54
8V1H	0.77	0.61	0.7	0.75	0.62	0.62	0.16	0.54	0.7	0.7
8V1I	0.72	0.42	0.63	0.53	0.5	0.6	0.32	0.62	0.7	0.7
8VT5	0.69	0.56	0.45	0.62	0.43	0.67	0.27	0.67	0.73	0.73
8XZL	0.62	0.63	0.59	0.56	0.52	0.54	0.46	0.44	0.39	0.39
9BLM	0.79	0.78	0.5	0.56	0.73	0.58	0.57	0.43	0.39	0.39
9DE6	0.73	0.21	0.8	0.77	0.76	0.64	0.52	0.65	0.28	0.28
9DE7	0.8	0.73	0.76	0.75	0.69	0.63	0.4	0.64	0.2	0.2
9ECQ	0.75	0.76	0.78	0.68	0.76	0.68	0.3	0.71	0.58	0.58
9ECR	0.64	0.66	0.7	0.66	0.67	0.62	0.11	0.61	0.49	0.49
R1211	0.63	0.59	0.62	0.73	0.59	0.53	0.26	0.53	0.2	0.2
R1263	0.63	0.55	0.56	0.58	0.6	0.62	0.83	0.75	0.75	0.75
R1271	0.73	0.57	0.73	0.72	0.48	0.57	0.28	0.52	0.72	0.72
R1293	0.55	0.48	0.54	0.6	0.3	0.46	0.2	0.51	0.01	0.01

PDB (Hard)	AF3	Boltz-1	Chai-1	HF3	NuFold	RhoFold+	RoseTTAFoldNA	trRosettaRNA	DRfold	DeepFoldRNA
**7MKT**	0.26	0.04	0.25	0.0	0.25	0.19	0.05	0.18	0.0	0.0
**7PS8**	0.22	0.1	0.19	0.16	0.18	0.19	0.03	0.18	0.0	0.0
**7Q48**	0.25	0.14	0.23	0.18	0.18	0.22	0.09	0.22	0.01	0.01
**7Q6L**	0.26	0.22	0.28	0.2	0.23	0.23	0.15	0.28	0.04	0.04
**7QA2**	0.28	0.23	0.25	0.25	0.26	0.23	0.14	0.24	0.21	0.21
**7SHX**	0.48	0.5	0.54	0.56	0.44	0.48	0.28	0.47	0.35	0.35
**7SXP**	0.24	0.12	0.23	0.0	0.21	0.22	0.08	0.19	0.06	0.06
**7UMC**	0.63	0.71	0.46	0.57	0.53	0.63	0.41	0.55	0.59	0.59
**7UMD**	0.65	0.6	0.57	0.54	0.46	0.6	0.38	0.46	0.58	0.58
**8BTZ**	0.74	0.63	0.77	0.76	0.64	0.66	0.35	0.54	0.55	0.55
**8BWT**	0.73	0.74	0.74	0.69	0.58	0.64	0.6	0.62	0.68	0.68
**8CLR**	0.78	0.75	0.7	0.55	0.61	0.78	0.41	0.61	0.14	0.14
**8FZA**	0.66	0.19	0.41	0.55	0.33	0.53	0.08	0.55	0.01	0.01
**8HB8**	0.37	0.29	0.31	0.29	0.17	0.39	0.13	0.31	0.0	0.0
**8I43**	0.51	0.77	0.72	0.73	0.57	0.7	0.38	0.64	0.73	0.73
**8I44**	0.69	0.8	0.71	0.61	0.6	0.69	0.5	0.52	0.75	0.75
**8I45**	0.68	0.58	0.66	0.7	0.38	0.69	0.15	0.52	0.44	0.44
**8I46**	0.71	0.74	0.71	0.69	0.47	0.68	0.13	0.64	0.66	0.66
**8ITS**	0.55	0.49	0.55	0.54	0.53	0.55	0.34	0.4	0.5	0.5
**8JHP**	0.49	0.5	0.63	0.47	0.43	0.5	0.4	0.5	0.08	0.08
**8K8B**	0.68	0.7	0.64	0.56	0.63	0.63	0.52	0.48	0.68	0.68
**8Q4O**	0.27	0.03	0.22	0.22	0.26	0.21	0.09	0.22	0.2	0.2
**8QO2**	0.54	0.34	0.34	0.47	0.56	0.53	0.21	0.54	0.07	0.07
**8QO3**	0.7	0.68	0.66	0.68	0.65	0.66	0.3	0.58	0.57	0.57
**8QO4**	0.58	0.46	0.52	0.54	0.53	0.55	0.16	0.52	0.45	0.45
**8QO5**	0.67	0.48	0.47	0.53	0.6	0.55	0.21	0.48	0.43	0.43
**8SCH**	0.73	0.64	0.62	0.57	0.62	0.6	0.42	0.57	0.6	0.6
**8T5O**	0.43	0.38	0.37	0.31	0.34	0.34	0.23	0.34	0.26	0.26
**8TNS**	0.23	0.28	0.28	0.02	0.23	0.22	0.03	0.23	0.0	0.0
**8UTG**	0.29	0.16	0.23	0.02	0.21	0.21	0.12	0.21	0.0	0.0
**8VCI**	0.61	0.52	0.6	0.51	0.41	0.55	0.26	0.55	0.61	0.61
**8ZAU**	0.58	0.49	0.46	0.56	0.5	0.61	0.19	0.45	0.01	0.01
**9BUN**	0.48	0.44	0.47	0.46	0.34	0.46	0.27	0.44	0.17	0.17
**9EOW**	0.7	0.56	0.71	0.66	0.74	0.72	0.22	0.56	0.73	0.73
**9EWW**	0.46	0.39	0.22	0.36	0.36	0.0	0.19	0.31	0.03	0.03

**9FM4**	0.67	0.64	0.68	0.56	0.64	0.64	0.42	0.63	0.59	0.59
**9FO8**	0.59	0.63	0.62	0.61	0.6	0.64	0.42	0.65	0.62	0.62
**9FO9**	0.6	0.63	0.66	0.58	0.69	0.66	0.53	0.68	0.68	0.68
**9IO0**	0.8	0.81	0.75	0.67	0.53	0.7	0.52	0.72	0.7	0.7
**9IO1**	0.68	0.75	0.67	0.44	0.54	0.66	0.4	0.66	0.73	0.73
**9IOR**	0.76	0.8	0.7	0.75	0.52	0.68	0.52	0.7	0.58	0.58
**9IOS**	0.7	0.74	0.77	0.68	0.51	0.6	0.22	0.66	0.04	0.04
**9IOU**	0.71	0.77	0.64	0.55	0.58	0.68	0.42	0.67	0.55	0.55
**R1205**	0.41	0.37	0.28	0.45	0.24	0.33	0.3	0.34	0.04	0.04
**R1209**	0.6	0.47	0.58	0.57	0.5	0.54	0.35	0.54	0.56	0.56
**R1288**	0.64	0.62	0.54	0.41	0.45	0.55	0.48	0.39	0.37	0.37
**R1296**	0.69	0.57	0.58	0.69	0.58	0.55	0.36	0.55	0.32	0.32

Table S6. Success rates for monomeric RNAs, easy subset, shown per method. Methods are shown in alphabetic order.

	AF3	Boltz-1	Chai-1	DeepFoldRNA	DRfold	HelixFold3	NuFold	RhoFold+	RosettaFoldNA	trRosettaRNA
**TM-score**	46.67%	40%	30%	40%	36.67%	23.33%	50%	30%	23.33%	23.33%
**GDT-TS**	70%	73.33%	53.33%	63.33%	63.33%	60%	66.67%	66.67%	63.33%	46.67%
**INF**	73.33%	60%	63.33%	63.33%	63.33%	70%	63.33%	40%	40%	20%
**lDDT**	24.14%	13.79%	20.69%	3.45%	3.45%	3.45%	20.69%	0%	3.45%	0%

Table S7. Success rates for monomeric RNAs, hard subset, shown per method. Methods are shown in alphabetic order.

	AF3	Boltz-1	Chai-1	DeepFoldRNA	DRfold	HelixFold3	NuFold	RhoFold+	RosettaFoldNA	trRosettaRNA
**TM-score**	4.08%	0%	2.04%	0%	2.04%	0%	2.04%	2.04%	4.08%	0%
**GDT-TS**	37.78%	46.94%	38.78%	42.86%	42.86%	38.78%	40.82%	36.73%	36.73%	28.57%
**INF**	46.94%	55.10%	42.86%	32.65%	32.65%	51.02%	32.65%	26.53%	22.45%	6.12%
**lDDT**	6.38%	10.64%	4.26%	0%	0%	2.13%	0%	2.13%	0%	0%

Table S8. MSA metrics for MSAs used in monomeric RNA prediction.

PDB	MSA Length	MSA Depth	Neff
**9IO1**	19	1	0.229416
**7KUC**	16	1	0.25
**7KUD**	13	1	0.27735
**9IOU**	19	1	0.229416
**9IOR**	19	1	0.229416
**8CLR**	14	1	0.267261
**8K8B**	19	1	0.229416
**9ECQ**	17	1	0.242536
**9ECR**	17	1	0.242536
**8I46**	19	1	0.229416
**8I45**	19	2	0.229416
**8I44**	19	2	0.229416
**8I43**	19	2	0.229416
**9IOS**	19	2	0.229416
**8BTZ**	238	2	0.129641
**8UTG**	21	2	0.218218
**9IO0**	19	2	0.229416
**7MKT**	23	4	0.208514
**8Q4O**	23	4	0.208514
**7Q6L**	22	5	0.426401
**8TNS**	24	8	0.262445
**7SXP**	22	11	0.351027
**9EOW**	29	11	0.185695
**7SHX**	94	12	0.618853
**8QO3**	118	12	0.659745
**8ZAU**	69	13	1.10354
**7UMD**	40	14	1.26491
**7QA2**	22	17	0.310918
**8CQ1**	44	17	0.332238
**9BUN**	48	19	2.59808
**R1205**	59	19	0.664464

**R1293**	82	24	0.891995
**7UME**	28	27	1.51586
**8BWT**	26	29	3.74255
**7PS8**	23	31	0.212046
**R1288**	58	33	3.46868
**9FM4**	25	33	1.417
**8HB8**	55	35	1.98798
**8JHP**	27	40	2.93201
**7Q48**	22	41	0.96854
**8ITS**	46	44	3.39772
**9FO8**	29	48	0.984255
**8UYG**	135	50	0.412469
**8UYM**	135	55	0.407737
**8QO4**	156	57	0.331625
**8VCI**	118	57	2.57761
**9FO9**	33	60	1.33322
**8QO2**	222	67	1.01534
**7WIA**	50	71	1.22852
**8XZL**	53	72	1.55539
**7RWR**	38	77	5.69322
**8QO5**	145	106	0.415382
**8UYP**	143	116	0.66996
**8FZA**	30	130	2.84942
**8SCF**	30	144	2.6225
**9BLM**	68	231	4.06194
**7UMC**	70	235	1.42697
**7KUB**	60	238	6.66719
**8Q6N**	46	295	4.61645
**8FB3**	34	307	2.96818
**9EWW**	120	350	7.75368
**8SCH**	68	457	32.5883
**8FCS**	71	923	26.2691
**8V1H**	75	1326	10.7762
**R1296**	72	1911	2.88311
**R1209**	72	1923	2.81855

**7V06**	43	2087	1.50854
**R1211**	90	2269	4.18309
**R1263**	64	2347	58.8498
**8VT5**	60	2596	86.6046
**8V1I**	57	3716	92.951
**8K2Z**	57	3835	94.7904
**8KEB**	72	3945	83.6221
**8UPT**	71	4095	188.474
**8K30**	56	4153	133.441
**9DE7**	58	4371	3.94147
**9DE6**	57	4575	3.5827
**8T5O**	124	9699	14.5406
**R1271**	77	17463	161.281

Table S9. DockQ scores for the easy and hard dataset of RNA-RNA and RNA-Protein complexes across all tested deep learning methods for complex prediction.

PDB (Easy)	AF3	Boltz-1	HF3	RoseTTAFoldNA
**7DA7**	0.017	0.139	0.13	0.005
**7P0V**	0.24	0.349	0.078	0.091
**7PMM**	0.019	0.172	0.018	0.019
**7QR3**	0.208	0.229	0.052	0.238
**7QR4**	0.604	0.691	0.07	0.616
**7SC6**	0.218	0.252	0.252	0.258
**7SCQ**	0.215	0.257	0.229	0.251
**7TNX**	0.814	0.479	0.307	0.037
**7TNY**	0.76	0.673	0.3	0.23
**7TO0**	0.734	0.293	0.595	0.054
**7TO1**	0.22	0.177	0.029	0.041
**7TO2**	0.008	0.009	0.008	0.037
**7U2A**	0.7	0.586	0.326	0.272
**7UIM**	0.035	0.008	0.007	0.003
**7XJZ**	0.312	0.33	0.283	0.042
**7YFQ**	0.591	0.594	0.17	0.028
**7YFX**	0.18	0.313	0.048	0.009
**7YFY**	0.614	0.528	0.223	0.018
**7YG6**	0.402	0.419	0.368	0.023
**7YGN**	0.782	0.723	0.024	0.011
**8AF0**	0.196	0.136	0.134	0.024
**8B9K**	0.016	0.041	0.006	0.015
**8CTI**	0.165	0.012	0.012	0.015
**8DVR**	0.145	0.056	0.046	0.065
**8DVS**	0.19	0.145	0.033	0.025
**8E28**	0.048	0.381	0.039	0.45
**8E29**	0.381	0.376	0.351	0.283
**8E2A**	0.06	0.326	0.05	0.172
**8GZP**	0.028	0.012	0.005	0.012
**8GZR**	0.124	0.152	0.02	0.008
**8H1B**	0.278	0.206	0.046	0.282
**8HNT**	0.124	0.008	0.015	0.009

**8HNW**	0.808	0.751	0.173	0.014
**8HUD**	0.441	0.698	0.174	0.016
**8I3Q**	0.009	0.043	0.015	0.009
**8ID2**	0.202	0.082	0.097	0.077
**8IDF**	0.141	0.16	0.013	0.013
**8IEW**	0.454	0.439	0.166	0.009
**8IFK**	0.37	0.663	0.102	0.006
**8IN8**	0.539	0.708	0.113	0.021
**8IPM**	0.498	0.477	0.018	0.024
**8J8H**	0.625	0.65	0.108	0.009
**8JTJ**	0.643	0.546	0.252	0.014
**8JTR**	0.307	0.184	0.268	0.014
**8KAG**	0.499	0.447	0.131	0.019
**8OKD**	0.579	0.136	0.006	0.153
**8OMR**	0.741	0.281	0.737	0.282
**8PJB**	0.249	0.107	0.108	0.164
**8PVV**	0.244	0.189	0.079	0.014
**8QG0**	0.445	0.247	0.109	0.024
**8R0S**	0.602	0.607	0.232	0.115
**8SCZ**	0.645	0.437	0.023	0.023
**8SD0**	0.81	0.564	0.044	0.217
**8SFH**	0.426	0.421	0.113	0.035
**8SFI**	0.744	0.684	0.097	0.026
**8SFJ**	0.54	0.502	0.102	0.036
**8SFL**	0.455	0.428	0.096	0.025
**8SFN**	0.66	0.559	0.09	0.025
**8SFR**	0.748	0.604	0.098	0.028
**8SH5**	0.589	0.682	0.501	0.035
**8SQU**	0.576	0.694	0.122	0.012
**8SXT**	0.295	0.32	0.284	0.197
**8T29**	0.724	0.578	0.713	0.02
**8UZA**	0.645	0.606	0.144	0.012
**8VIV**	0.794	0.835	0.05	0.065
**8VMB**	0.609	0.54	0.628	0.02
**8WRR**	0.192	0.427	0.134	0.011
**8WRS**	0.74	0.204	0.155	0.141

**8WRT**	0.012	0.041	0.031	0.018
**8WRV**	0.184	0.18	0.158	0.138
**8WS9**	0.572	0.183	0.163	0.142
**8X5V**	0.764	0.7	0.177	0.013
**8XYC**	0.208	0.172	0.196	0.006
**8Y03**	0.46	0.396	0.039	0.099
**8Y04**	0.875	0.566	0.236	0.071
**8Y0C**	0.733	0.692	0.172	0.014
**8Y0D**	0.727	0.63	0.135	0.009
**8Y7Z**	0.491	0.631	0.1	0.008
**8Z1F**	0.013	0.298	0.015	0.019
**8ZMI**	0.226	0.243	0.253	0.251
**8ZNJ**	0.461	0.119	0.118	0.008
**8ZTY**	0.124	0.016	0.017	0.035
**9B2K**	0.376	0.369	0.125	0.007
**9CMP**	0.435	0.568	0.235	0.043
**9DCF**	0.026	0.052	0.02	0.024
**9DTT**	0.011	0.009	0.009	0.01
**9E7G**	0.282	0.624	0.26	0.013
**9ENE**	0.621	0.137	0.022	0.246
**9FCV**	0.009	0.055	0.025	0.021
**9IIY**	0.527	0.53	0.208	0.017
**9IIZ**	0.216	0.508	0.213	0.024
**9IJ0**	0.51	0.637	0.24	0.066
**9IJ1**	0.235	0.204	0.225	0.012
**9IJ2**	0.505	0.486	0.425	0.017
**9IJ3**	0.598	0.66	0.244	0.027
**9IJ4**	0.62	0.618	0.244	0.022
**9IJ5**	0.617	0.624	0.481	0.028
**9L6Y**	0.793	0.706	0.199	0.12
**9MX3**	0.514	0.262	0.259	0.25
**M1209**	0.487	0.485	0.454	0.016
**M1293**	0.639	0.591	0.708	0.02
**M1296**	0.615	0.444	0.654	0.284

PDB (Hard)	AF3	Boltz-1	HF3	RoseTTAFoldNA
**7ECJ**	0.819	0.813	0.823	0.843
**7ECK**	0.742	0.776	0.804	0.853
**7PDU**	0.22	0.231	0.213	0.102
**7UR5**	0.009	0.014	0.114	0.028
**7UU3**	0.159	0.154	0.155	0.064
**7V94**	0.461	0.273	0.096	0.009
**7YOJ**	0.171	0.2	0.154	0.012
**8D9G**	0.109	0.112	0.073	0.001
**8DC2**	0.259	0.178	0.179	0.006
**8DMB**	0.326	0.202	0.176	0.009
**8FTI**	0.008	0.009	0.013	0.013
**8H1J**	0.14	0.251	0.101	0.006
**8HB1**	0.419	0.278	0.413	0.074
**8HB3**	0.154	0.2	0.378	0.075
**8HBA**	0.009	0.017	0.007	0.013
**8HIO**	0.018	0.038	0.025	0.017
**8HKE**	0.128	0.29	0.134	0.019
**8HZJ**	0.044	0.04	0.012	0.017
**8HZK**	0.026	0.065	0.014	0.012
**8HZL**	0.011	0.012	0.008	0.003
**8IBW**	0.163	0.133	0.111	0.011
**8IBY**	0.005	0.016	0.022	0.033
**8IBZ**	0.006	0.015	0.007	0.009
**8J9G**	0.016	0.018	0.018	0.013
**8JL0**	0.426	0.575	0.105	0.008
**8K0Y**	0.007	0.234	0.011	0.026
**8OPP**	0.035	0.033	0.024	0.034
**8PM4**	0.172	0.285	0.167	0.01
**8T2P**	0.003	0.005	0.003	0.149
**8VFS**	0.664	0.631	0.172	0.01
**8WFA**	0.188	0.167	0.142	0.019
**8WRU**	0.185	0.182	0.099	0.01
**8XCC**	0.234	0.207	0.159	0.006
**8XTP**	0.006	0.004	0.008	0.003
**8Y9L**	0.005	0.017	0.015	0.01

**8Y9M**	0.164	0.093	0.132	0.009
**8YDC**	0.462	0.524	0.455	0.215
**8Z9K**	0.03	0.203	0.018	0.068
**9BXE**	0.749	0.761	0.638	0.571
**9C0I**	0.327	0.418	0.02	0.053
**9CER**	0.015	0.008	0.017	0.017
**9CEU**	0.495	0.208	0.091	0.015
**9CPD**	0.763	0.802	0.75	0.808
**9CPJ**	0.726	0.749	0.843	0.699
**9DIB**	0.007	0.007	0.005	0.01
**9DIG**	0.007	0.113	0.008	0.007
**9DII**	0.007	0.015	0.007	0.009
**9GBZ**	0.191	0.184	0.235	0.007
**9H82**	0.01	0.055	0.008	0.011
**9H83**	0.156	0.116	0.067	0.118
**9I8B**	0.698	0.646	0.615	0.641
**9JSP**	0.671	0.735	0.141	0.022
**9KAD**	0.077	0.082	0.081	0.082
**9KPH**	0.004	0.005	0.006	0.003
**9KPO**	0.01	0.011	0.007	0.003
**9NVU**	0.113	0.15	0.122	0.007

Table S10. US-Align TM-score for the easy and hard dataset of RNA-RNA and RNA-Protein complexes across all tested deep learning methods for complex prediction.

PDB (Easy)	AF3	Boltz-1	HF3	RoseTTAFoldNA
7DA7	0.68238	0.57465	0.62719	0.07523
7P0V	0.77768	0.82717	0.32433	0.77706
7PMM	0.48528	0.49073	0.17282	0.44737
7QR3	0.39158	0.40516	0.25644	0.4191
7QR4	0.77069	0.85496	0.35405	0.75873
7SC6	0.77964	0.77917	0.78353	0.7763
7SCQ	0.77089	0.78851	0.80694	0.77661
7TNX	0.98362	0.95107	0.25868	0.75037
7TNY	0.98441	0.97381	0.2446	0.7521
7TO0	0.98748	0.94507	0.97961	0.76202
7TO1	0.97538	0.96086	0.24711	0.75843
7TO2	0.9207	0.92805	0.91718	0.78521
7U2A	0.98435	0.97064	0.95207	0.92681
7UIM	0.5168	0.4655	0.48037	0.42106
7XJZ	0.88173	0.83494	0.87286	0.86782
7YFQ	0.94467	0.93084	0.94417	0.85695
7YFX	0.96902	0.91365	0.32918	0.89305
7YFY	0.9344	0.90134	0.21376	0.87786
7YG6	0.96636	0.93558	0.92061	0.90728
7YGN	0.96658	0.9208	0.2671	0.91899
8AF0	0.47963	0.44992	0.47859	0.41133
8B9K	0.77317	0.78386	0.23704	0.80669
8CTI	0.14623	0.12883	0.11586	0.12765
8DVR	0.97202	0.92154	0.33038	0.75148
8DVS	0.97258	0.95852	0.21688	0.77372
8E28	0.98095	0.96964	0.27198	0.97211
8E29	0.95959	0.95379	0.95899	0.92375
8E2A	0.73535	0.72989	0.23854	0.73072
8GZP	0.88433	0.70251	0.23755	0.66439
8GZR	0.59603	0.53753	0.30916	0.07532
8H1B	0.91553	0.90471	0.36195	0.91236
8HNT	0.76931	0.67264	0.76499	0.34801

**8HNW**	0.96207	0.95648	0.23096	0.5144
**8HUD**	0.94158	0.90837	0.20313	0.52965
**8I3Q**	0.89348	0.80123	0.79597	0.30555
**8ID2**	0.39234	0.40109	0.18109	0.40613
**8IDF**	0.8883	0.88179	0.24869	0.66293
**8IEW**	0.80364	0.73911	0.26905	0.18359
**8IFK**	0.9666	0.95494	0.23947	0.45867
**8IN8**	0.96901	0.96218	0.23583	0.57859
**8IPM**	0.94243	0.95224	0.29408	0.86989
**8J8H**	0.96568	0.96416	0.28226	0.56216
**8JTJ**	0.93881	0.9013	0.88178	0.37751
**8JTR**	0.91679	0.81789	0.90913	0.56184
**8KAG**	0.77924	0.73098	0.19862	0.33769
**8OKD**	0.93424	0.74392	0.16875	0.74798
**8OMR**	0.95391	0.89134	0.96433	0.86706
**8PJB**	0.92669	0.96137	0.23901	0.81857
**8PVV**	0.75219	0.64515	0.30346	0.34557
**8QG0**	0.88685	0.71653	0.26352	0.37786
**8R0S**	0.9418	0.94469	0.23761	0.85986
**8SCZ**	0.96132	0.92831	0.28326	0.76782
**8SD0**	0.98451	0.96255	0.23677	0.77847
**8SFH**	0.73886	0.7533	0.23776	0.50922
**8SFI**	0.81756	0.90454	0.21078	0.2851
**8SFJ**	0.91453	0.93111	0.25286	0.30795
**8SFL**	0.88308	0.87613	0.19488	0.28258
**8SFN**	0.97897	0.93795	0.2307	0.33218
**8SFR**	0.93807	0.9083	0.21848	0.35464
**8SH5**	0.796	0.91206	0.86445	0.42137
**8SQU**	0.95349	0.95189	0.23469	0.53367
**8SXT**	0.97329	0.96105	0.95187	0.87415
**8T29**	0.88649	0.85284	0.88686	0.41879
**8UZA**	0.94706	0.9001	0.83514	0.32341
**8VIV**	0.98677	0.98451	0.9007	0.91329
**8VMB**	0.80322	0.81183	0.83655	0.448
**8WRR**	0.76056	0.59731	0.20658	0.1928
**8WRS**	0.78284	0.62674	0.29219	0.19219

**8WRT**	0.67088	0.65712	0.20077	0.34267
**8WRV**	0.24591	0.23865	0.27196	0.2136
**8WS9**	0.87178	0.60265	0.22242	0.14617
**8X5V**	0.95384	0.93225	0.23206	0.66527
**8XYC**	0.75621	0.34103	0.1973	0.19681
**8Y03**	0.98372	0.79085	0.20213	0.78604
**8Y04**	0.89483	0.87396	0.81693	846
**8Y0C**	0.97885	0.91162	0.23683	0.32485
**8Y0D**	0.87298	0.82815	0.26562	0.46993
**8Y7Z**	0.97079	0.96892	0.22446	0.58415
**8Z1F**	0.90223	0.91534	0.90319	0.86902
**8ZMI**	0.76712	0.78734	0.81184	0.78836
**8ZNJ**	0.83708	0.70436	0.2751	0.36913
**8ZTY**	0.90509	0.77088	0.24299	0.21067
**9B2K**	0.62131	0.46813	0.51632	0.07643
**9CMP**	0.83141	0.8796	0.83206	0.75011
**9DCF**	0.51036	0.48265	0.2342	0.44771
**9DTT**	0.69908	0.70334	0.23827	0.67592
**9E7G**	0.79653	0.86392	0.81707	0.44714
**9ENE**	0.94547	0.75799	0.22573	0.83554
**9FCV**	0.94187	0.93031	0.24242	0.28248
**9IIY**	0.82046	0.84463	0.23278	0.76315
**9IIZ**	0.87398	0.8762	0.28758	0.84357
**9IJ0**	0.96531	0.89928	0.24559	0.89297
**9IJ1**	0.86269	0.9334	0.24692	0.79405
**9IJ2**	0.89722	0.8787	0.88688	0.84138
**9IJ3**	0.89747	0.90681	0.23174	0.80687
**9IJ4**	0.89837	0.94005	0.30536	0.82411
**9IJ5**	0.91378	0.91342	0.86361	0.83706
**9L6Y**	0.93578	0.90055	0.32427	0.75667
**9MX3**	0.9207	0.85612	0.87274	0.8873
**M1209**	0.70825	0.69966	0.70289	0.41442
**M1293**	0.86699	0.85049	0.92418	0.42098
**M1296**	0.84565	0.8054	0.82882	0.86068

PDB (Hard)	AF3	Boltz-1	HF3	RoseTTAFoldNA
7ECJ	0.40557	0.38658	0.37352	0.37754
7ECK	0.36327	0.33774	0.34593	0.40694
7PDU	0.17907	0.1871	0.18779	0.19043
7UR5	0.33999	0.33997	0.27816	0.21843
7UU3	0.31505	0.52534	0.41843	0.324
7V94	0.90962	0.75409	0.26342	0.18965
7YOJ	0.55721	0.31427	0.17996	0.18112
8D9G	0.78669	0.59792	0.21521	0.04273
8DC2	0.2267	0.23532	0.21237	0.20627
8DMB	0.68894	0.55588	0.59474	0.43294
8FTI	0.39743	0.31596	0.22863	0.13854
8H1J	0.69409	0.73203	0.21971	0.68244
8HB1	0.26654	0.26121	0.21682	0.13788
8HB3	0.26476	0.19766	0.24865	0.15076
8HBA	0.20181	0.18175	0.2041	0.18426
8HIO	0.72656	0.71955	0.703	0.27637
8HKE	0.89922	0.9099	0.1954	0.25108
8HZJ	0.25012	0.15663	0.21519	0.21989
8HZK	0.15646	0.22655	0.2359	0.2142
8HZL	0.21044	0.22514	0.15519	0.17922
8IBW	0.90644	0.86954	0.24262	0.41667
8IBY	0.69892	0.72561	0.71502	0.59132
8IBZ	0.71375	0.72918	0.21358	0.55067
8J9G	0.92547	0.904	0.26941	0.50171
8JL0	0.96514	0.93952	0.22576	0.47953
8K0Y	0.40995	0.24211	0.37985	0.29716
8OPP	0.91676	0.91338	0.25527	0.43416
8PM4	0.89967	0.90732	0.23339	0.49274
8T2P	0.27536	0.26496	0.30018	0.26251
8VFS	0.56308	0.42473	0.26751	0.21285
8WFA	0.64092	0.6142	0.58334	0.16776
8WRU	0.36546	0.36247	0.21141	0.18721
8XCC	0.32835	0.36532	0.38843	0.17443
8XTP	0.31137	0.16753	0.18356	0.10159

**8Y9L**	0.33144	0.25834	0.24522	0.22683
**8Y9M**	0.35869	0.22221	0.19136	0.18751
**8YDC**	0.35945	0.41154	0.37799	0.16162
**8Z9K**	0.21981	0.24911	0.21575	0.19706
**9BXE**	0.36054	0.36525	0.32187	0.22662
**9C0I**	0.72677	0.70673	0.21248	0.69273
**9CER**	0.29769	0.21999	0.2057	0.19335
**9CEU**	0.83352	0.84067	0.22311	0.20472
**9CPD**	0.3723	0.41024	0.33386	0.44313
**9CPJ**	0.36602	0.43385	0.48665	0.43372
**9DIB**	0.27955	0.19055	0.21449	0.17011
**9DIG**	0.20794	0.24915	0.21747	0.20767
**9DII**	0.22615	0.18507	0.19235	0.21609
**9GBZ**	0.73822	0.75835	0.71892	0.3614
**9H82**	0.08563	0.08929	0.08556	0.08492
**9H83**	0.21739	0.22869	0.1587	0.26549
**9I8B**	0.44226	0.39022	0.33738	0.35249
**9JSP**	0.9287	0.95196	0.31057	0.64381
**9KAD**	0.30271	0.33702	0.2411	0.38966
**9KPH**	0.20907	0.19558	0.22268	0.1403
**9KPO**	0.16545	0.15807	0.21169	0.12563
**9NVU**	0.50552	0.51557	0.23458	0.20092

Table S11. F1 score for the easy and hard dataset of RNA-RNA and RNA-Protein complexes across all tested deep learning methods for complex prediction.

PDB (Easy)	AF3	Boltz-1	HF3	RoseTTAFoldNA
7DA7	0.0	0.029	0.085	0.0
7P0V	0.4	0.424	0.05	0.132
7PMM	0.0	0.129	0.0	0.0
7QR3	0.0	0.0	0.0	0.0
7QR4	0.917	0.855	0.152	0.832
7SC6	0.0	0.0	0.0	0.0
7SCQ	0.0	0.0	0.0	0.0
7TNX	0.758	0.493	0.0	0.0
7TNY	0.699	0.569	0.0	0.0
7TO0	0.677	0.0	0.929	0.0
7TO1	0.633	0.451	0.0	0.052
7TO2	0.0	0.0	0.0	0.0
7U2A	0.641	0.353	0.0	0.0
7UIM	0.0	0.0	0.0	0.0
7XJZ	0.346	0.338	0.463	0.047
7YFQ	0.571	0.5	0.012	0.0
7YFX	0.503	0.503	0.049	0.0
7YFY	0.495	0.394	0.03	0.0
7YG6	0.759	0.683	0.748	0.0
7YGN	0.736	0.727	0.0	0.0
8AF0	0.0	0.0	0.0	0.0
8B9K	0.0	0.043	0.0	0.0
8CTI	0.0	0.0	0.0	0.0
8DVR	0.483	0.028	0.012	0.162
8DVS	0.604	0.406	0.015	0.0
8E+28	0.0	0.351	0.014	0.547
8E+29	0.735	0.652	0.649	0.414
8E2A	0.083	0.653	0.049	0.241
8GZP	0.014	0.0	0.0	0.0
8GZR	0.0	0.3	0.0	0.0
8H1B	0.0	0.0	0.0	0.0

**8HNT**	0.0	0.0	0.0	0.0
**8HNW**	0.788	0.73	0.003	0.0
**8HUD**	0.0	0.597	0.012	0.0
**8I3Q**	0.0	0.05	0.0	0.0
**8ID2**	0.143	0.027	0.0	0.0
**8IDF**	0.145	0.199	0.0	0.0
**8IEW**	0.384	0.23	0.0	0.0
**8IFK**	0.096	0.4	0.0	0.0
**8IN8**	0.302	0.609	0.0	0.0
**8IPM**	0.086	0.059	0.0	0.0
**8J8H**	0.405	0.5	0.0	0.0
**8JTJ**	0.675	0.672	0.0	0.0
**8JTR**	0.578	0.369	0.476	0.014
**8KAG**	0.337	0.21	0.0	0.0
**8OKD**	0.372	0.0	0.0	0.0
**8OMR**	0.776	0.0	0.794	0.0
**8PJB**	0.217	0.023	0.056	0.133
**8PVV**	0.011	0.0	0.0	0.0
**8QG0**	0.371	0.062	0.0	0.0
**8R0S**	0.412	0.463	0.0	0.0
**8SCZ**	0.798	0.639	0.014	0.0
**8SD0**	0.836	0.638	0.026	0.253
**8SFH**	0.47	0.364	0.0	0.0
**8SFI**	0.776	0.789	0.0	0.0
**8SFJ**	0.901	0.589	0.0	0.0
**8SFL**	0.498	0.45	0.0	0.0
**8SFN**	0.58	0.43	0.0	0.0
**8SFR**	0.8	0.672	0.0	0.0
**8SH5**	0.714	0.875	0.615	0.0
**8SQU**	0.465	0.485	0.0	0.0
**8SXT**	0.0	0.028	0.0	0.0
**8T29**	0.889	0.545	0.881	0.0
**8UZA**	0.767	0.712	0.0	0.0
**8VIV**	0.866	0.88	0.084	0.135
**8VMB**	0.725	0.857	0.867	0.0
**8WRR**	0.0	0.102	0.0	0.0

**8WRS**	0.711	0.0	0.0	0.0
**8WRT**	0.0	0.034	0.05	0.0
**8WRV**	0.0	0.0	0.0	0.0
**8WS9**	0.0	0.0	0.0	0.0
**8X5V**	0.821	0.705	0.0	0.0
**8XYC**	0.031	0.0	0.0	0.0
**8Y03**	0.704	0.624	0.0	0.18
**8Y04**	0.909	0.416	0.0	0.0
**8Y0C**	0.881	0.808	0.0	0.0
**8Y0D**	0.769	0.637	0.0	0.0
**8Y7Z**	0.151	0.448	0.0	0.0
**8Z1F**	0.0	0.354	0.0	0.0
**8ZMI**	0.0	0.04	0.0	0.0
**8ZNJ**	0.204	0.0	0.008	0.0
**8ZTY**	0.338	0.0	0.003	0.009
**9B2K**	0.567	0.584	0.0	0.0
**9CMP**	0.438	0.4	0.0	0.0
**9DCF**	0.0	0.0	0.0	0.0
**9DTT**	0.0	0.0	0.0	0.0
**9E7G**	0.0	0.839	0.0	0.0
**9ENE**	0.48	0.0	0.0	0.0
**9FCV**	0.0	0.057	0.0	0.005
**9IIY**	0.505	0.481	0.0	0.0
**9IIZ**	0.0	0.485	0.0	0.0
**9IJ0**	0.304	0.462	0.0	0.0
**9IJ1**	0.0	0.01	0.0	0.0
**9IJ2**	0.367	0.207	0.368	0.0
**9IJ3**	0.583	0.698	0.021	0.0
**9IJ4**	0.636	0.565	0.033	0.0
**9IJ5**	0.61	0.659	0.372	0.0
**9L6Y**	0.944	0.88	0.208	0.231
**9MX3**	0.306	0.0	0.0	0.0
**M1209**	0.935	0.906	0.897	0.0
**M1293**	0.8	0.571	0.921	0.0
**M1296**	0.8	0.25	1.0	0.0

PDB (Hard)	AF3	Boltz-1	HF3	RoseTTAFoldNA
**7ECJ**	1.0	1.0	1.0	1.0
**7ECK**	0.926	0.926	0.926	0.926
**7PDU**	0.617	0.594	0.585	0.407
**7UR5**	0.0	0.0	0.0	0.0
**7UU3**	0.0	0.0	0.0	0.0
**7V94**	0.309	0.0	0.0	0.0
**7YOJ**	0.0	0.006	0.0	0.0
**8D9G**	0.0	0.0	0.0	0.0
**8DC2**	0.0	0.0	0.0	0.0
**8DMB**	0.167	0.0	0.0	0.0
**8FTI**	0.0	0.005	0.012	0.026
**8H1J**	0.0	0.0	0.0	0.0
**8HB1**	0.706	0.628	0.658	0.235
**8HB3**	0.426	0.475	0.671	0.241
**8HBA**	0.0	0.0	0.0	0.0
**8HIO**	0.0	0.0	0.0	0.0
**8HKE**	0.0	0.028	0.004	0.0
**8HZJ**	0.061	0.029	0.0	0.0
**8HZK**	0.019	0.107	0.0	0.0
**8HZL**	0.0	0.0	0.0	0.0
**8IBW**	0.0	0.0	0.006	0.0
**8IBY**	0.0	0.004	0.017	0.017
**8IBZ**	0.0	0.009	0.0	0.0
**8J9G**	0.0	0.0	0.0	0.0
**8JL0**	0.075	0.436	0.0	0.0
**8K0Y**	0.0	0.034	0.0	0.0
**8OPP**	0.0	0.0	0.02	0.018
**8PM4**	0.0	0.0	0.006	0.0
**8T2P**	0.0	0.0	0.0	0.286
**8VFS**	0.87	0.812	0.394	0.0
**8WFA**	0.0	0.0	0.0	0.006
**8WRU**	0.0	0.0	0.0	0.0
**8XCC**	0.063	0.0	0.0	0.0

**8XTP**	0.0	0.0	0.0	0.0
**8Y9L**	0.0	0.016	0.0	0.0
**8Y9M**	0.0	0.0	0.0	0.0
**8YDC**	0.907	0.828	0.826	0.595
**8Z9K**	0.031	0.451	0.0	0.0
**9BXE**	1.0	0.964	1.0	0.967
**9C0I**	0.183	0.151	0.0	0.0
**9CER**	0.011	0.0	0.011	0.005
**9CEU**	0.407	0.027	0.0	0.0
**9CPD**	0.954	0.954	0.954	0.928
**9CPJ**	1.0	0.985	0.985	0.943
**9DIB**	0.0	0.0	0.0	0.0
**9DIG**	0.0	0.351	0.0	0.0
**9DII**	0.0	0.0	0.0	0.0
**9GBZ**	0.0	0.0	0.0	0.0
**9H82**	0.0	0.0	0.0	0.0
**9H83**	0.0	0.0	0.0	0.0
**9I8B**	0.982	0.964	0.991	0.945
**9JSP**	0.585	0.653	0.0	0.0
**9KAD**	0.0	0.0	0.0	0.0
**9KPH**	0.0	0.0	0.0	0.0
**9KPO**	0.0	0.022	0.0	0.0
**9NVU**	0.0	0.002	0.011	0.0

Table S12. Fnat score for the easy and hard dataset of RNA-RNA and RNA-Protein complexes across all tested deep learning methods for complex prediction.

PDB (Easy)	AF3	Boltz-1	HF3	RoseTTAFoldNA
7DA7	0.0	0.034	0.052	0.0
7P0V	0.457	0.514	0.057	0.143
7PMM	0.0	0.155	0.0	0.0
7QR3	0.0	0.0	0.0	0.0
7QR4	0.926	0.926	0.148	0.963
7SC6	0.0	0.0	0.0	0.0
7SCQ	0.0	0.0	0.0	0.0
7TNX	0.714	0.486	0.0	0.0
7TNY	0.741	0.603	0.0	0.0
7TO0	0.703	0.0	0.963	0.0
7TO1	0.53	0.386	0.0	0.036
7TO2	0.0	0.0	0.0	0.0
7U2A	0.82	0.48	0.0	0.0
7UIM	0.0	0.0	0.0	0.0
7XJZ	0.423	0.635	0.481	0.058
7YFQ	0.75	0.75	0.012	0.0
7YFX	0.494	0.575	0.057	0.0
7YFY	0.627	0.518	0.029	0.0
7YG6	0.759	0.741	0.69	0.0
7YGN	0.812	0.833	0.0	0.0
8AF0	0.0	0.0	0.0	0.0
8B9K	0.0	0.053	0.0	0.0
8CTI	0.0	0.0	0.0	0.0
8DVR	0.354	0.025	0.013	0.101
8DVS	0.478	0.339	0.017	0.0
8E28	0.0	0.379	0.016	0.589
8E29	0.768	0.768	0.67	0.536
8E2A	0.083	0.786	0.048	0.345
8GZP	0.015	0.0	0.0	0.0
8GZR	0.0	0.273	0.0	0.0
8H1B	0.0	0.0	0.0	0.0
8HNT	0.0	0.0	0.0	0.0

**8HNW**	0.809	0.73	0.003	0.0
**8HUD**	0.0	0.767	0.017	0.0
**8I3Q**	0.0	0.058	0.0	0.0
**8ID2**	0.111	0.026	0.0	0.0
**8IDF**	0.13	0.185	0.0	0.0
**8IEW**	0.331	0.221	0.0	0.0
**8IFK**	0.102	0.367	0.0	0.0
**8IN8**	0.265	0.609	0.0	0.0
**8IPM**	0.176	0.059	0.0	0.0
**8J8H**	0.378	0.4	0.0	0.0
**8JTJ**	0.685	0.733	0.0	0.0
**8JTR**	0.567	0.453	0.459	0.018
**8KAG**	0.762	0.524	0.0	0.0
**8OKD**	0.368	0.0	0.0	0.0
**8OMR**	0.963	0.0	0.926	0.0
**8PJB**	0.2	0.022	0.056	0.111
**8PVV**	0.01	0.0	0.0	0.0
**8QG0**	0.302	0.047	0.0	0.0
**8R0S**	0.606	0.475	0.0	0.0
**8SCZ**	0.785	0.667	0.022	0.0
**8SD0**	0.892	0.723	0.036	0.241
**8SFH**	0.635	0.554	0.0	0.0
**8SFI**	0.877	0.836	0.0	0.0
**8SFJ**	0.847	0.604	0.0	0.0
**8SFL**	0.642	0.529	0.0	0.0
**8SFN**	0.593	0.393	0.0	0.0
**8SFR**	0.862	0.695	0.0	0.0
**8SH5**	0.556	0.778	0.444	0.0
**8SQU**	0.435	0.471	0.0	0.0
**8SXT**	0.0	0.016	0.0	0.0
**8T29**	1.0	0.75	0.839	0.0
**8UZA**	0.735	0.744	0.0	0.0
**8VIV**	0.887	0.887	0.081	0.081
**8VMB**	0.833	0.75	0.867	0.0
**8WRR**	0.0	0.097	0.0	0.0
**8WRS**	0.711	0.0	0.0	0.0

**8WRT**	0.0	0.04	0.04	0.0
**8WRV**	0.0	0.0	0.0	0.0
**8WS9**	0.0	0.0	0.0	0.0
**8X5V**	0.819	0.706	0.0	0.0
**8XYC**	0.025	0.0	0.0	0.0
**8Y03**	0.73	0.716	0.0	0.191
**8Y04**	0.938	0.412	0.0	0.0
**8Y0C**	0.907	0.829	0.0	0.0
**8Y0D**	0.84	0.796	0.0	0.0
**8Y7Z**	0.123	0.462	0.0	0.0
**8Z1F**	0.0	0.5	0.0	0.0
**8ZMI**	0.0	0.043	0.0	0.0
**8ZNJ**	0.156	0.0	0.011	0.0
**8ZTY**	0.307	0.0	0.003	0.007
**9B2K**	0.776	0.8	0.0	0.0
**9CMP**	0.575	0.525	0.0	0.0
**9DCF**	0.0	0.0	0.0	0.0
**9DTT**	0.0	0.0	0.0	0.0
**9E7G**	0.0	0.839	0.0	0.0
**9ENE**	0.569	0.0	0.0	0.0
**9FCV**	0.0	0.048	0.0	0.005
**9IIY**	0.418	0.463	0.0	0.0
**9IIZ**	0.0	0.615	0.0	0.0
**9IJ0**	0.308	0.59	0.0	0.0
**9IJ1**	0.0	0.01	0.0	0.0
**9IJ2**	0.5	0.333	0.389	0.0
**9IJ3**	0.524	0.714	0.024	0.0
**9IJ4**	0.533	0.543	0.038	0.0
**9IJ5**	0.524	0.671	0.293	0.0
**9L6Y**	0.944	0.917	0.417	0.25
**9MX3**	0.367	0.0	0.0	0.0
**M1209**	0.935	0.935	0.839	0.0
**M1293**	1.0	1.0	0.906	0.0
**M1296**	1.0	0.5	1.0	0.0

PDB (Hard)	AF3	Boltz-1	HF3	RoseTTAFoldNA
**7ECJ**	1.0	1.0	1.0	1.0
**7ECK**	1.0	1.0	1.0	1.0
**7PDU**	0.506	0.506	0.469	0.272
**7UR5**	0.0	0.0	0.0	0.0
**7UU3**	0.0	0.0	0.0	0.0
**7V94**	0.313	0.0	0.0	0.0
**7YOJ**	0.0	0.007	0.0	0.0
**8D9G**	0.0	0.0	0.0	0.0
**8DC2**	0.0	0.0	0.0	0.0
**8DMB**	0.146	0.0	0.0	0.0
**8FTI**	0.0	0.005	0.015	0.02
**8H1J**	0.0	0.0	0.0	0.0
**8HB1**	0.72	0.573	0.653	0.16
**8HB3**	0.329	0.418	0.633	0.165
**8HBA**	0.0	0.0	0.0	0.0
**8HIO**	0.0	0.0	0.0	0.0
**8HKE**	0.0	0.026	0.004	0.0
**8HZJ**	0.077	0.077	0.0	0.0
**8HZK**	0.045	0.136	0.0	0.0
**8HZL**	0.0	0.0	0.0	0.0
**8IBW**	0.0	0.0	0.012	0.0
**8IBY**	0.0	0.009	0.036	0.064
**8IBZ**	0.0	0.017	0.0	0.0
**8J9G**	0.0	0.0	0.0	0.0
**8JL0**	0.06	0.48	0.0	0.0
**8K0Y**	0.0	0.667	0.0	0.0
**8OPP**	0.0	0.0	0.022	0.022
**8PM4**	0.0	0.0	0.006	0.0
**8T2P**	0.0	0.0	0.0	0.441
**8VFS**	0.909	0.848	0.379	0.0
**8WFA**	0.0	0.0	0.0	0.005
**8WRU**	0.0	0.0	0.0	0.0
**8XCC**	0.043	0.0	0.0	0.0
**8XTP**	0.0	0.0	0.0	0.0

**8Y9L**	0.0	0.023	0.0	0.0
**8Y9M**	0.0	0.0	0.0	0.0
**8YDC**	0.917	0.854	0.792	0.521
**8Z9K**	0.024	0.39	0.0	0.0
**9BXE**	1.0	0.931	1.0	1.0
**9C0I**	0.165	0.131	0.0	0.0
**9CER**	0.012	0.0	0.019	0.012
**9CEU**	0.412	0.027	0.0	0.0
**9CPD**	0.969	0.969	0.969	1.0
**9CPJ**	1.0	1.0	0.97	1.0
**9DIB**	0.0	0.0	0.0	0.0
**9DIG**	0.0	0.309	0.0	0.0
**9DII**	0.0	0.0	0.0	0.0
**9GBZ**	0.0	0.0	0.0	0.0
**9H82**	0.0	0.0	0.0	0.0
**9H83**	0.0	0.0	0.0	0.0
**9I8B**	1.0	0.981	1.0	0.963
**9JSP**	0.5	0.667	0.0	0.0
**9KAD**	0.0	0.0	0.0	0.0
**9KPH**	0.0	0.0	0.0	0.0
**9KPO**	0.0	0.022	0.0	0.0
**9NVU**	0.0	0.002	0.011	0.0

Table S13. iRMSD for the easy and hard dataset of RNA-RNA and RNA-Protein complexes across all tested deep learning methods for complex prediction.

PDB (Easy)	AF3	Boltz-1	HF3	RoseTTAFoldNA
7DA7	45.608	17.468	35.885	122.977
7P0V	7.836	4.516	8.852	11.355
7PMM	30.034	9.933	25.467	39.634
7QR3	25.962	16.033	24.889	23.075
7QR4	845	1.129	7.515	1.083
7SC6	44.392	12.405	16.865	21.428
7SCQ	36.092	18.713	21.934	22.987
7TNX	1.349	3.519	22.146	17.698
7TNY	1.628	2.634	26.944	21.949
7TO0	1.731	18.254	4.681	18.051
7TO1	12.243	11.757	26.437	17.452
7TO2	26.435	25.919	27.342	14.104
7U2A	3.05	3.783	14.74	26.816
7UIM	35.941	34.343	36.676	49.215
7XJZ	6.238	7.498	7.469	20.085
7YFQ	3.109	3.376	23.839	18.748
7YFX	16.963	5.811	24.226	21.303
7YFY	3.474	4.619	27.186	33.55
7YG6	4.277	4.072	4.828	12.162
7YGN	1.178	1.511	21.403	18.213
8AF0	14.163	18.777	18.585	14.307
8B9K	20.623	17.716	33.872	28.514
8CTI	12.584	12.099	17.907	15.689
8DVR	15.681	12.243	22.49	22.422
8DVS	14.742	14.617	26.771	20.886
8E+28	9.039	3.097	22.618	3.137
8E+29	5.834	6.512	5.745	7.068
8E2A	7.122	4.53	20.314	6.053
8GZP	14.955	23.117	33.958	24.865
8GZR	27.396	23.1	21.212	64.848
8H1B	8.3	21.191	9.817	7.129
8HNT	25.598	27.862	39.685	30.43

**8HNW**	3.612	3.417	35.271	31.749
**8HUD**	7.557	3.184	27.821	27.141
**8I3Q**	29.062	15.112	18.085	35.696
**8ID2**	14.472	17.292	16.74	9.312
**8IDF**	7.18	7.655	23.653	26.407
**8IEW**	10.024	9.503	23.461	42.472
**8IFK**	5.368	2.625	25.219	37.034
**8IN8**	3.19	1.909	24.521	44.522
**8IPM**	7.257	11.913	16.153	18.819
**8J8H**	2.737	2.498	29.345	36.219
**8JTJ**	8.703	8.755	7.862	37.422
**8JTR**	8.52	14.826	8.077	31.778
**8KAG**	9.178	11.25	24.09	29.169
**8OKD**	2.793	23.973	40.526	24.542
**8OMR**	2.59	27.068	2.73	26.349
**8PJB**	4.633	5.592	29.337	6.338
**8PVV**	9.641	19.656	20.211	22.661
**8QG0**	6.222	16.689	22.753	26.615
**8R0S**	2.887	2.448	22.752	17.199
**8SCZ**	1.428	2.988	22.581	16.2
**8SD0**	1.22	2.688	23.323	10.929
**8SFH**	9.78	9.822	13.499	33.996
**8SFI**	2.97	2.39	19.615	41.176
**8SFJ**	7.445	5.59	30.872	37.555
**8SFL**	6.63	6.331	27.845	40.1
**8SFN**	3.842	4.78	31.216	40.541
**8SFR**	1.865	3.638	22.351	41.6
**8SH5**	1.134	1.381	3.294	33.46
**8SQU**	3.249	2.619	28.191	30.923
**8SXT**	5.085	4.858	5.916	15.071
**8T29**	981	2.354	747	29.861
**8UZA**	7.72	5.882	32.232	36.786
**8VIV**	1.246	1.038	14.443	10.776
**8VMB**	1.012	1.413	862	24.169
**8WRR**	21.07	14.98	20.476	32.906
**8WRS**	2.186	18.602	23.2	35.885

**8WRT**	24.063	15.303	23.447	29.386
**8WRV**	26.366	25.696	26.554	33.851
**8WS9**	2.433	21.929	23.054	41.873
**8X5V**	4.095	3.984	31.852	25.23
**8XYC**	31.39	29.407	35.223	44.558
**8Y03**	4.09	7.899	30.126	13.845
**8Y04**	1.27	2.559	18.679	20.059
**8Y0C**	2.711	3.673	23.838	25.915
**8Y0D**	2.332	4.791	27.985	30.603
**8Y7Z**	3.763	2.563	26.196	42.084
**8Z1F**	25.687	3.13	23.73	18.254
**8ZMI**	24.92	18.041	19.743	24.746
**8ZNJ**	5.951	21.924	22.855	39.279
**8ZTY**	17.105	21.661	31.249	30.049
**9B2K**	12.648	13.399	27.446	49.375
**9CMP**	5.453	3.054	21.839	21.095
**9DCF**	26.821	17.614	17.624	17.741
**9DTT**	23.397	21.926	34.842	31.366
**9E7G**	5.644	1.017	11.568	25.616
**9ENE**	2.814	25.876	28.014	25.902
**9FCV**	25.676	12.601	26.349	31.21
**9IIY**	6.917	6.698	24.608	28.973
**9IIZ**	23.371	2.733	22.8	28.122
**9IJ0**	3.6	4.297	26.715	16.127
**9IJ1**	18.671	18.767	28.495	23.102
**9IJ2**	2.428	2.706	3.32	27.151
**9IJ3**	3.544	2.94	25.76	27.375
**9IJ4**	3.749	2.736	21.889	24.609
**9IJ5**	3.486	2.851	5.029	19.165
**9L6Y**	1.414	1.802	11.217	10.544
**9MX3**	3.91	19.322	19.351	14.929
**M1209**	5.891	6.013	9.553	28.603
**M1293**	1.628	2.03	0.9	36.983
**M1296**	1.604	2.463	827	13.977

PDB (Hard)	AF3	Boltz-1	HF3	RoseTTAFoldNA
7ECJ	1.457	1.496	1.42	1.303
7ECK	2.121	1.815	1.545	1.236
7PDU	18.518	15.976	17.966	34.284
7UR5	25.178	28.444	49.857	11.207
7UU3	12.277	8.277	9.356	8.484
7V94	7.206	26.796	25.935	40.971
7YOJ	30.107	35.147	34.585	43.373
8D9G	21.704	29.921	32.153	168.744
8DC2	19.959	22.02	20.128	52.495
8DMB	22.783	19.251	25.925	45.808
8FTI	33.884	39.168	38.365	39.657
8H1J	29.947	34.056	29.525	36.529
8HB1	7.709	10.491	7.037	21.536
8HB3	14.426	14.388	8.412	21.857
8HBA	24.957	20.529	25.683	19.535
8HIO	18.305	21.563	29.567	21.466
8HKE	46.895	23.97	30.263	40.766
8HZJ	13.685	16.424	17.859	17.431
8HZK	21.252	11.434	18.417	21.053
8HZL	20.06	17.367	20.726	35.97
8IBW	23.266	48.636	22.472	34.791
8IBY	39.641	29.668	28.225	28.905
8IBZ	36.399	28.821	36.886	34.877
8J9G	26.926	37.583	26.999	38.515
8JL0	4.22	2.806	22.901	40.612
8K0Y	22.803	13.951	17.161	9.403
8OPP	13.612	15.803	23.389	16.522
8PM4	18.91	11.461	29.831	39.575
8T2P	32.984	27.936	37.792	63.405
8VFS	2.857	3.178	18.219	31.425
8WFA	20.898	17.922	32.993	35.497
8WRU	19.217	20.712	28.717	44.452
8XCC	26.503	21.743	24.666	33.785
8XTP	47.923	47.04	40.0	73.311

**8Y9L**	35.324	28.716	27.068	30.659
**8Y9M**	37.409	32.926	34.393	32.297
**8YDC**	5.676	4.897	7.094	17.389
**8Z9K**	18.287	10.004	18.202	12.676
**9BXE**	1.964	1.726	3.406	4.48
**9C0I**	22.553	25.098	21.293	34.504
**9CER**	26.779	26.609	30.633	29.004
**9CEU**	9.305	9.51	21.487	29.019
**9CPD**	1.765	1.491	1.949	1.583
**9CPJ**	2.371	2.16	1.241	2.736
**9DIB**	33.625	21.111	30.729	31.353
**9DIG**	40.317	18.68	32.468	33.364
**9DII**	37.315	24.626	32.985	33.468
**9GBZ**	27.604	16.304	30.493	28.611
**9H82**	17.189	3.987	21.045	15.32
**9H83**	27.215	10.75	27.3	28.331
**9I8B**	2.515	3.243	3.831	3.416
**9JSP**	2.411	2.373	17.711	26.23
**9KAD**	9.19	8.768	8.945	8.774
**9KPH**	39.183	48.568	32.752	52.512
**9KPO**	39.632	40.693	35.107	52.721
**9NVU**	31.755	31.654	31.999	44.794

Table S14. LRMSD for the easy and hard dataset of RNA-RNA and RNA-Protein complexes across all tested deep learning methods for complex prediction.

PDB (Easy)	AF3	Boltz-1	HF3	RoseTTAFoldNA
7DA7	82.096	45.099	61.416	190.728
7P0V	15.648	9.72	20.308	23.687
7PMM	50.42	31.236	78.158	98.658
7QR3	44.827	35.364	54.94	69.719
7QR4	22.356	8.359	53.981	15.696
7SC6	60.731	73.1	72.83	73.367
7SCQ	53.776	63.273	35.383	66.194
7TNX	659	7.028	18.487	31.638
7TNY	2.405	4.951	36.481	35.448
7TO0	2.607	43.12	7.163	33.637
7TO1	23.48	22.124	27.944	28.835
7TO2	56.577	54.902	58.222	25.409
7U2A	5.124	6.941	22.679	45.605
7UIM	25.205	55.391	60.856	100.662
7XJZ	9.23	12.444	12.104	32.5
7YFQ	5.757	6.17	50.592	33.683
7YFX	43.276	12.926	28.571	54.951
7YFY	6.059	8.111	44.712	78.843
7YG6	11.886	10.479	12.252	35.439
7YGN	2.572	3.736	32.011	52.945
8AF0	60.267	49.416	53.239	59.1
8B9K	40.194	33.093	69.28	40.177
8CTI	87.093	72.189	106.323	73.596
8DVR	30.784	22.3	22.997	27.208
8DVS	28.518	27.872	29.2	31.01
8E28	23.265	7.328	25.807	7.295
8E29	12.566	12.71	12.391	13.969
8E2A	35.148	26.531	25.968	23.703
8GZP	33.9	46.531	71.869	45.815
8GZR	56.662	69.718	71.683	78.761
8H1B	16.995	59.95	52.055	16.111
8HNT	54.832	63.409	84.316	70.911

**8HNW**	1.939	2.833	58.991	72.19
**8HUD**	17.173	3.776	40.551	62.76
**8I3Q**	54.791	32.821	42.488	52.564
**8ID2**	23.862	23.42	32.401	42.032
**8IDF**	14.716	14.379	44.523	44.502
**8IEW**	18.626	12.454	36.103	95.867
**8IFK**	11.781	4.188	54.271	90.573
**8IN8**	6.41	3.379	79.912	115.397
**8IPM**	25.097	21.265	41.823	52.444
**8J8H**	4.213	3.114	51.337	99.654
**8JTJ**	11.477	11.977	17.495	79.321
**8JTR**	12.272	27.496	12.632	57.904
**8KAG**	11.52	12.755	37.719	52.521
**8OKD**	4.985	38.463	105.14	94.381
**8OMR**	3.607	43.538	3.684	44.96
**8PJB**	9.367	15.518	14.177	12.141
**8PVV**	20.089	35.713	41.677	58.751
**8QG0**	8.712	24.609	53.061	54.302
**8R0S**	5.78	4.961	29.95	32.245
**8SCZ**	6.574	9.505	40.303	33.432
**8SD0**	2.235	5.153	26.562	10.62
**8SFH**	25.602	25.439	60.797	64.112
**8SFI**	2.699	4.61	49.865	60.778
**8SFJ**	9.875	9.978	51.793	49.511
**8SFL**	9.913	10.696	52.81	55.41
**8SFN**	7.03	8.662	42.84	42.175
**8SFR**	5.17	5.201	70.013	63.615
**8SH5**	13.316	10.369	38.542	79.316
**8SQU**	5.182	2.867	75.774	74.241
**8SXT**	16.762	16.346	19.122	32.214
**8T29**	19.642	34.421	18.559	68.89
**8UZA**	11.693	8.6	97.709	70.345
**8VIV**	2.778	2.101	33.618	26.331
**8VMB**	38.451	33.331	37.177	47.021
**8WRR**	66.039	18.637	43.855	117.757
**8WRS**	4.299	62.556	45.43	92.1

**8WRT**	46.464	30.434	37.376	36.659
**8WRV**	61.603	68.869	54.209	88.476
**8WS9**	2.783	62.762	57.154	133.668
**8X5V**	6.944	6.915	49.466	62.184
**8XYC**	59.008	81.215	59.293	82.827
**8Y03**	8.004	9.661	23.495	26.333
**8Y04**	1.167	6.332	49.451	46.063
**8Y0C**	4.665	4.888	41.861	58.77
**8Y0D**	4.793	6.426	46.525	65.134
**8Y7Z**	7.422	5.222	59.569	95.374
**8Z1F**	43.388	16.701	41.872	37.013
**8ZMI**	56.108	44.122	38.934	60.186
**8ZNJ**	9.354	43.32	51.387	78.119
**8ZTY**	34.734	40.359	39.304	26.103
**9B2K**	21.409	24.787	75.872	97.986
**9CMP**	14.435	7.971	50.398	45.175
**9DCF**	55.841	44.45	62.987	70.326
**9DTT**	49.17	57.973	54.587	49.734
**9E7G**	43.303	32.033	41.961	53.518
**9ENE**	4.769	39.199	50.771	38.302
**9FCV**	53.622	24.866	30.343	34.485
**9IIY**	4.755	4.987	34.84	50.488
**9IIZ**	44.693	12.296	48.201	45.827
**9IJ0**	6.768	7.247	54.219	45.768
**9IJ1**	46.251	47.878	30.512	52.695
**9IJ2**	10.627	11.064	12.602	50.733
**9IJ3**	3.328	2.718	42.865	43.095
**9IJ4**	2.775	3.568	34.468	46.341
**9IJ5**	2.953	4.368	5.486	46.459
**9L6Y**	2.751	4.337	19.204	26.891
**9MX3**	7.431	32.774	32.711	29.899
**M1209**	30.159	29.192	47.845	50.614
**M1293**	39.637	27.885	16.745	90.566
**M1296**	19.031	46.207	22.964	36.817

PDB (Hard)	AF3	Boltz-1	HF3	RoseTTAFoldNA
7ECJ	2.105	2.204	2.121	1.772
7ECK	2.941	2.481	2.375	1.651
7PDU	20.513	18.248	19.259	46.279
7UR5	87.305	53.249	94.244	33.412
7UU3	40.274	32.828	28.092	58.762
7V94	14.738	61.105	54.907	65.564
7YOJ	46.925	54.473	59.619	68.923
8D9G	53.406	73.204	90.384	287.719
8DC2	26.963	74.005	54.536	109.691
8DMB	37.362	60.32	50.357	90.4
8FTI	55.642	59.08	56.196	62.294
8H1J	109.389	73.697	46.311	78.005
8HB1	8.468	15.059	7.819	34.352
8HB3	22.891	18.61	9.043	35.344
8HBA	56.179	39.303	62.412	45.536
8HIO	45.999	45.277	64.095	58.599
8HKE	79.903	61.615	70.877	61.728
8HZJ	40.381	44.671	48.571	39.098
8HZK	49.291	40.336	44.921	46.768
8HZL	49.906	50.189	62.004	93.172
8IBW	46.741	98.354	59.571	69.589
8IBY	73.622	44.347	52.104	45.294
8IBZ	68.744	51.502	62.553	53.724
8J9G	58.222	56.294	55.451	63.356
8JL0	9.349	4.702	56.592	88.496
8K0Y	67.65	52.993	53.065	35.564
8OPP	26.537	26.895	38.626	30.656
8PM4	41.214	26.76	54.475	73.145
8T2P	111.046	76.47	99.597	122.333
8VFS	3.351	3.408	21.871	49.475
8WFA	36.079	30.717	63.407	44.253
8WRU	61.417	45.372	63.235	95.164
8XCC	39.201	55.806	70.479	84.687
8XTP	63.846	84.288	55.498	97.651

**8Y9L**	73.397	53.492	41.137	50.491
**8Y9M**	69.472	54.467	63.768	65.024
**8YDC**	10.342	6.483	8.013	23.325
**8Z9K**	34.278	17.211	37.755	17.579
**9BXE**	3.174	2.495	4.874	6.748
**9C0I**	45.113	49.686	64.414	61.496
**9CER**	48.912	57.918	49.249	44.969
**9CEU**	15.573	17.506	81.483	64.797
**9CPD**	2.827	2.247	2.7	1.917
**9CPJ**	2.965	2.489	1.631	3.331
**9DIB**	62.457	68.62	77.076	49.425
**9DIG**	59.961	53.591	57.348	62.906
**9DII**	58.579	40.892	60.089	51.728
**9GBZ**	50.86	31.615	57.926	75.719
**9H82**	57.701	41.055	61.571	53.312
**9H83**	76.309	40.084	46.003	76.779
**9I8B**	3.808	4.5	5.405	4.285
**9JSP**	2.529	2.152	59.839	89.36
**9KAD**	16.694	16.125	16.211	16.125
**9KPH**	88.066	70.632	69.137	91.129
**9KPO**	50.378	84.146	58.743	100.493
**9NVU**	66.665	53.973	61.386	110.49

Table S15. Mean IF DockQ scores across all methods for RNA-protein and RNA-RNA complex prediction.

	**AF3**	**Boltz-1**	**HF3**	**RF2NA**
**RNA-protein**	0.281	0.256	0.075	0.039
**RNA-RNA**	0.406	0.42	0.373	0.138

Supplementary Figures

A

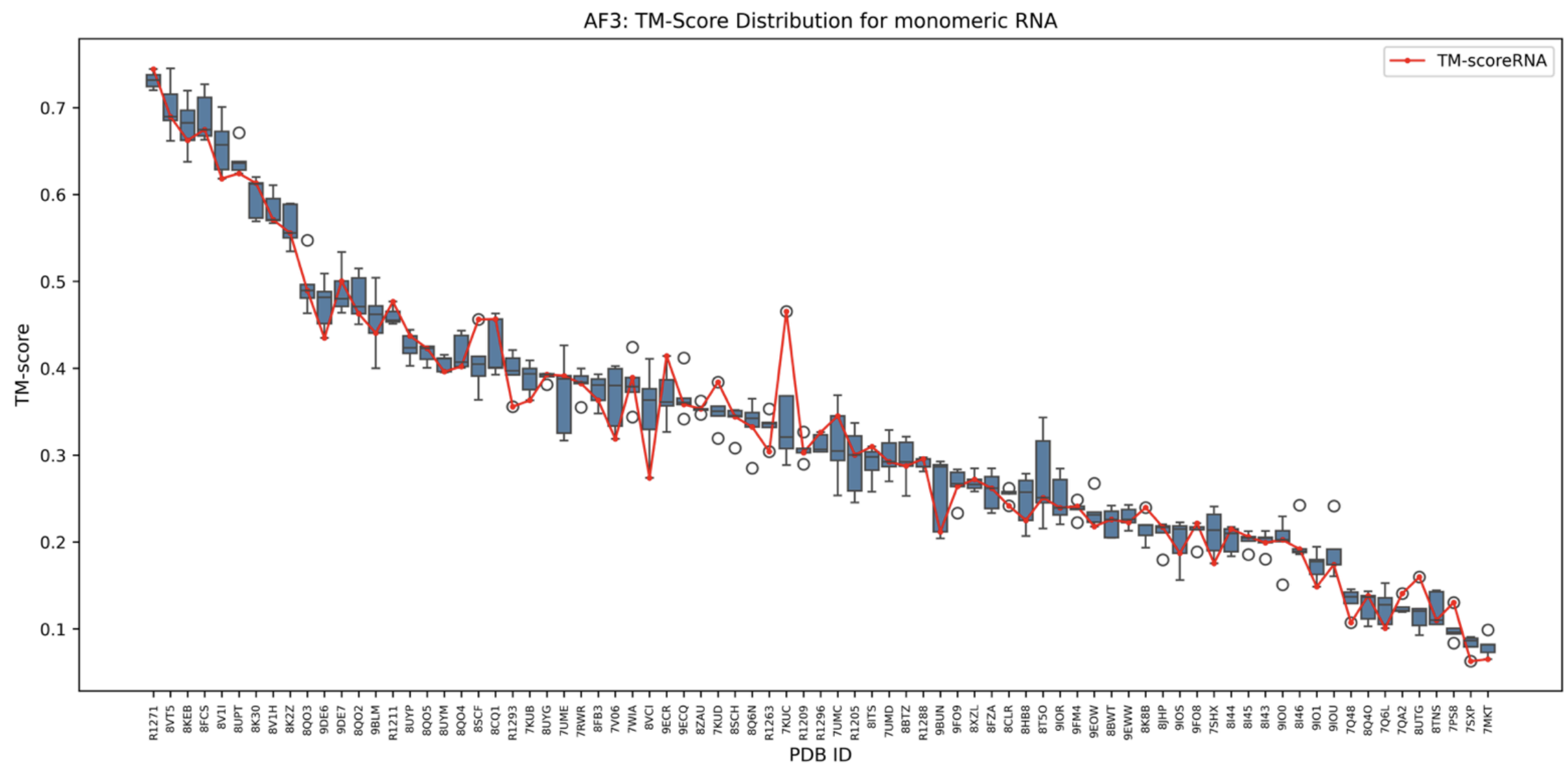

B

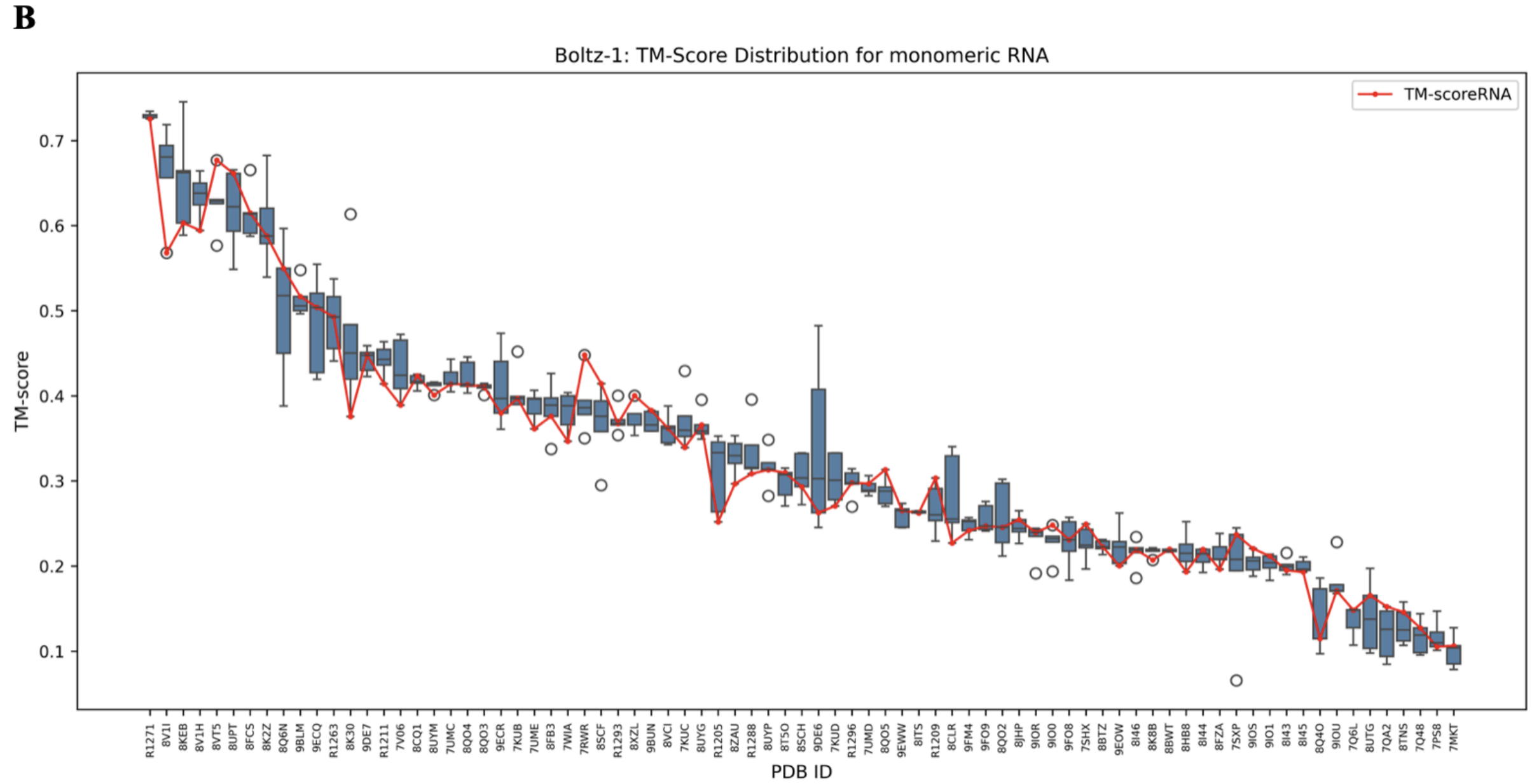

Figure S1. Distribution of TM-scores across n = 5 generated single-chain RNA models for AlphaFold3 (A) and Boltz-1 (B). TM-scores for each ID are shown as box plots, ordered from highest to lowest mean TM-score. The red line across the plot connects the top-ranked models of each ID.

A

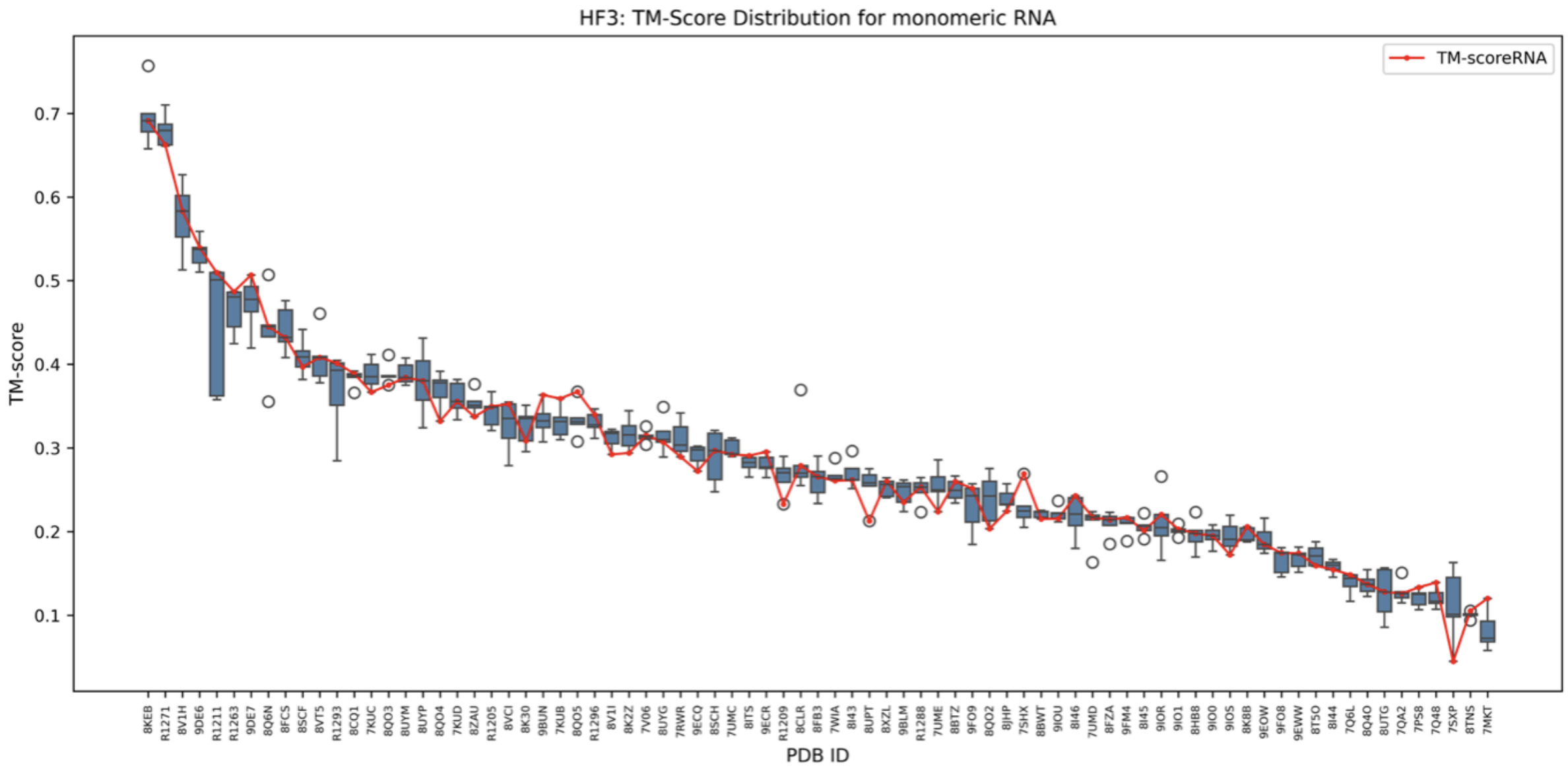

B

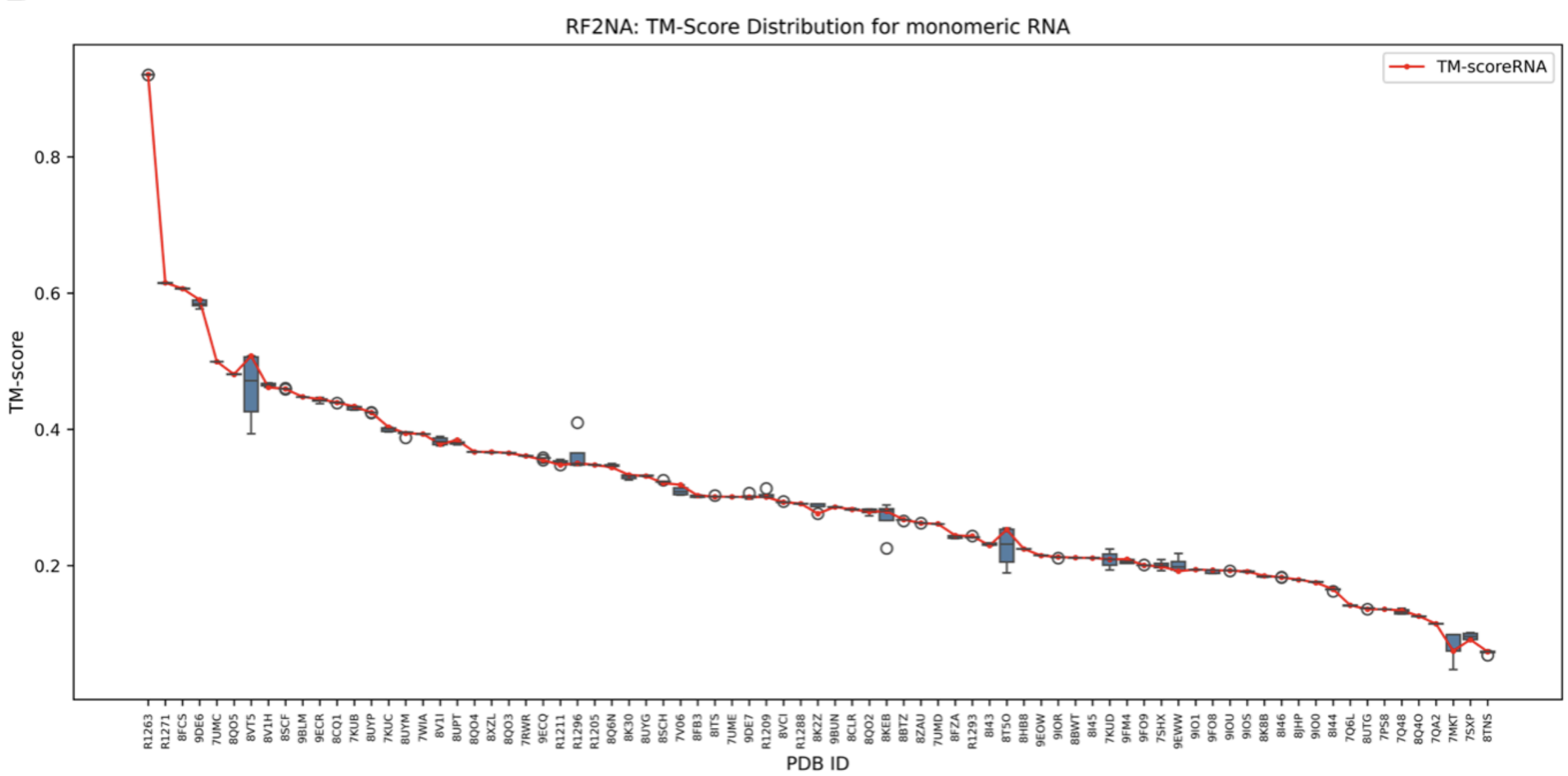

Figure S2. Distribution of TM-scores across *n = 5* generated single-chain RNA models for HF3 (A) and RF2NA (B). For each ID, TM-scores are visualized as box plots, ordered from highest to lowest mean TM-score. A red line connects the top-ranked model for each ID.

A

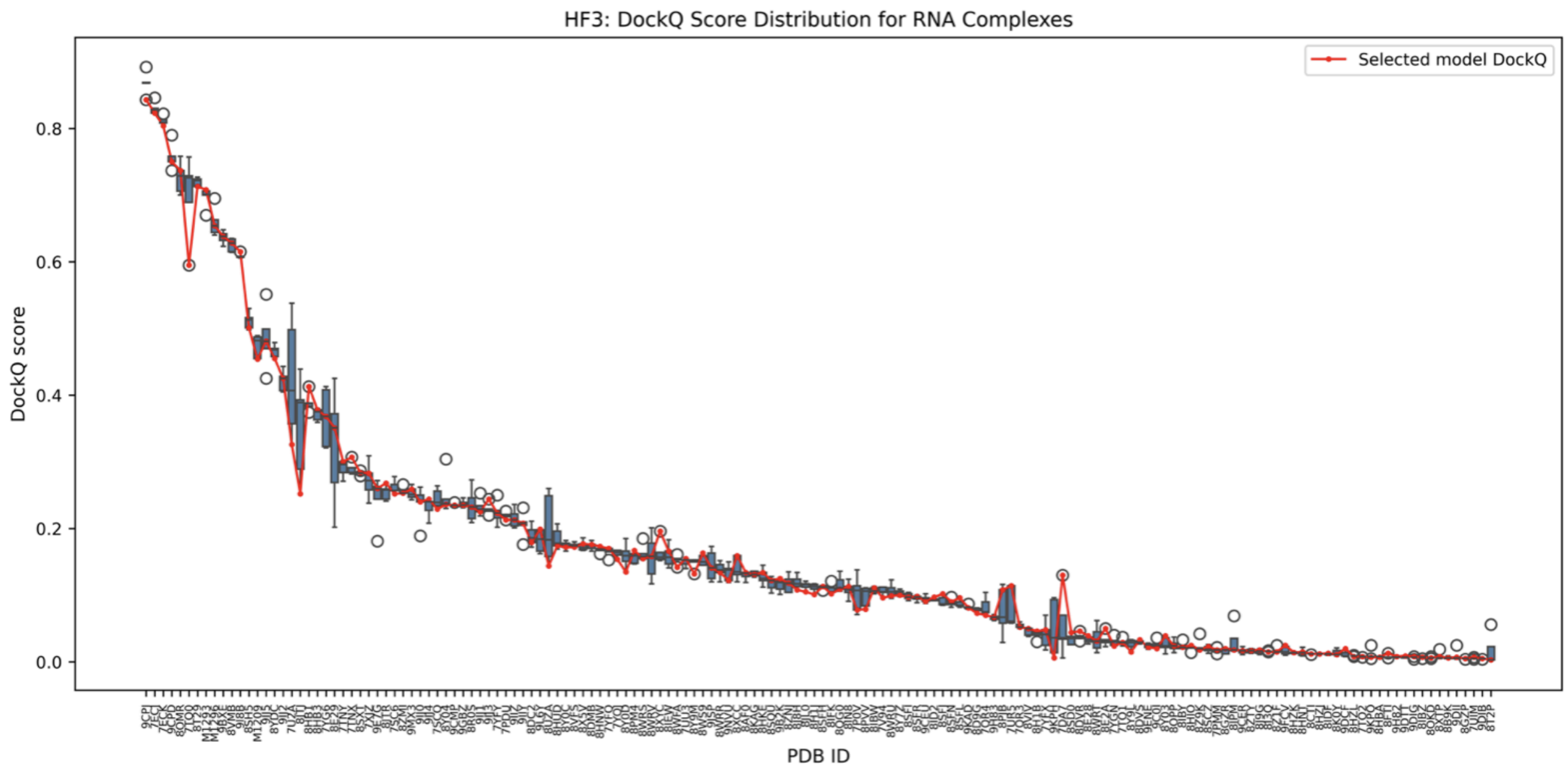

B

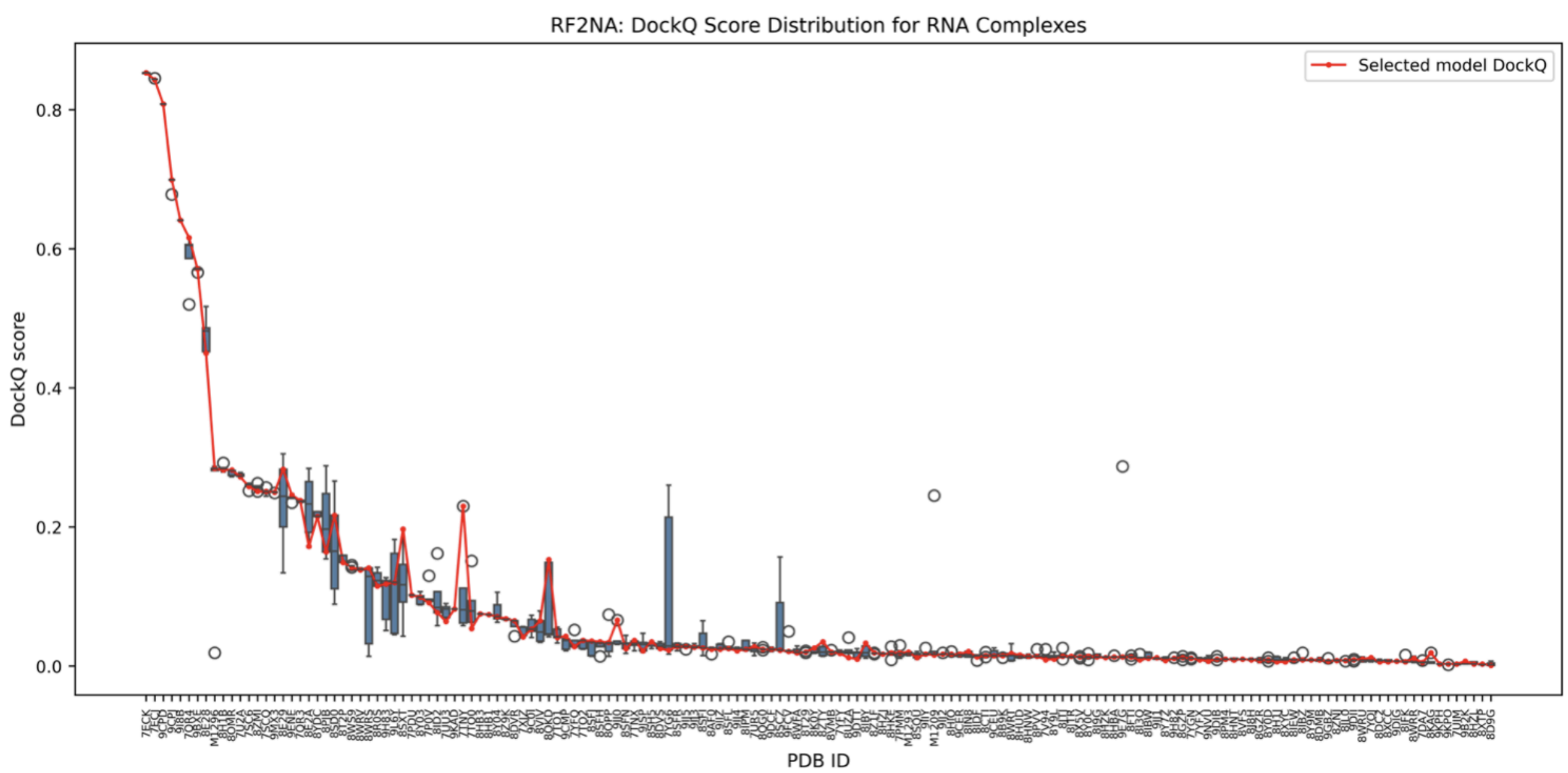

Figure S3. Distribution of DockQ scores across $n = 5$ generated RNA complex models for AF3 (A) and Boltz-1 (B). For each ID, DockQ scores are visualized as box plots, ordered from highest to lowest mean DockQ score. A red line connects the top-ranked model for each ID.

A

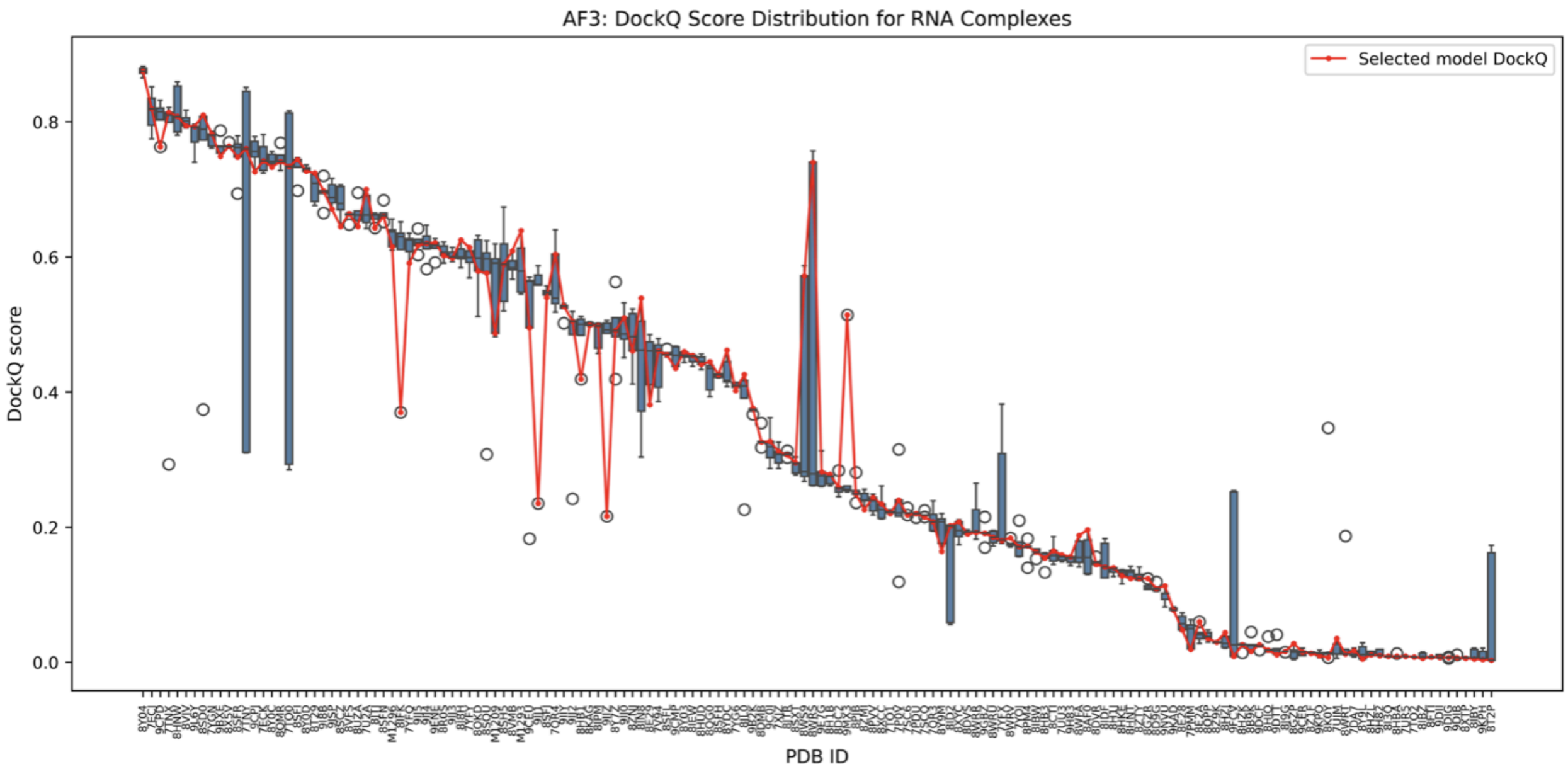

B

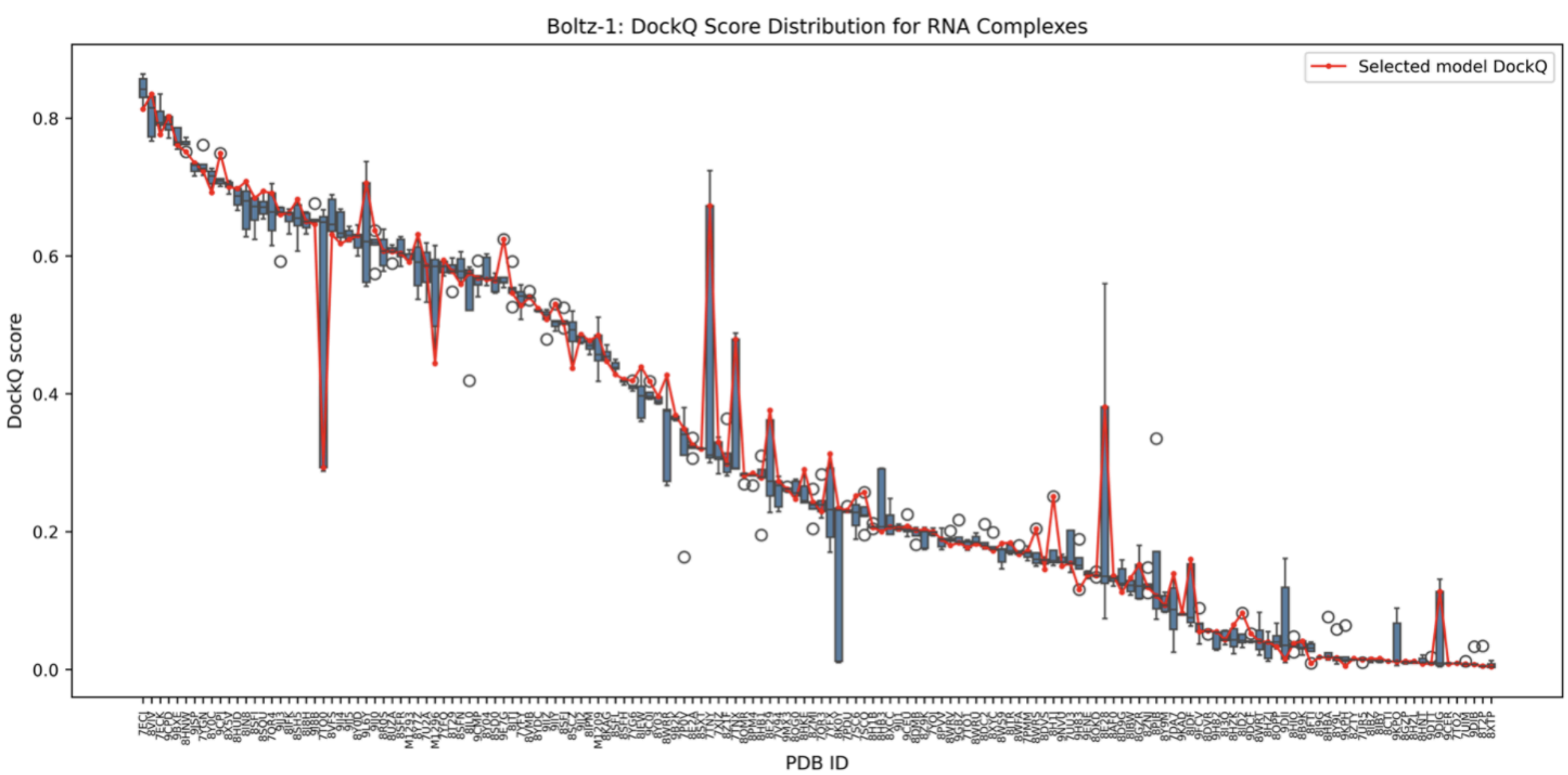

Figure S4. Distribution of DockQ scores across $n = 5$ generated RNA complex models for HF3 (A) and RF2NA (B). For each ID, DockQ scores are visualized as box plots, ordered by decreasing mean DockQ score. A red line connects the top-ranked model for each ID.

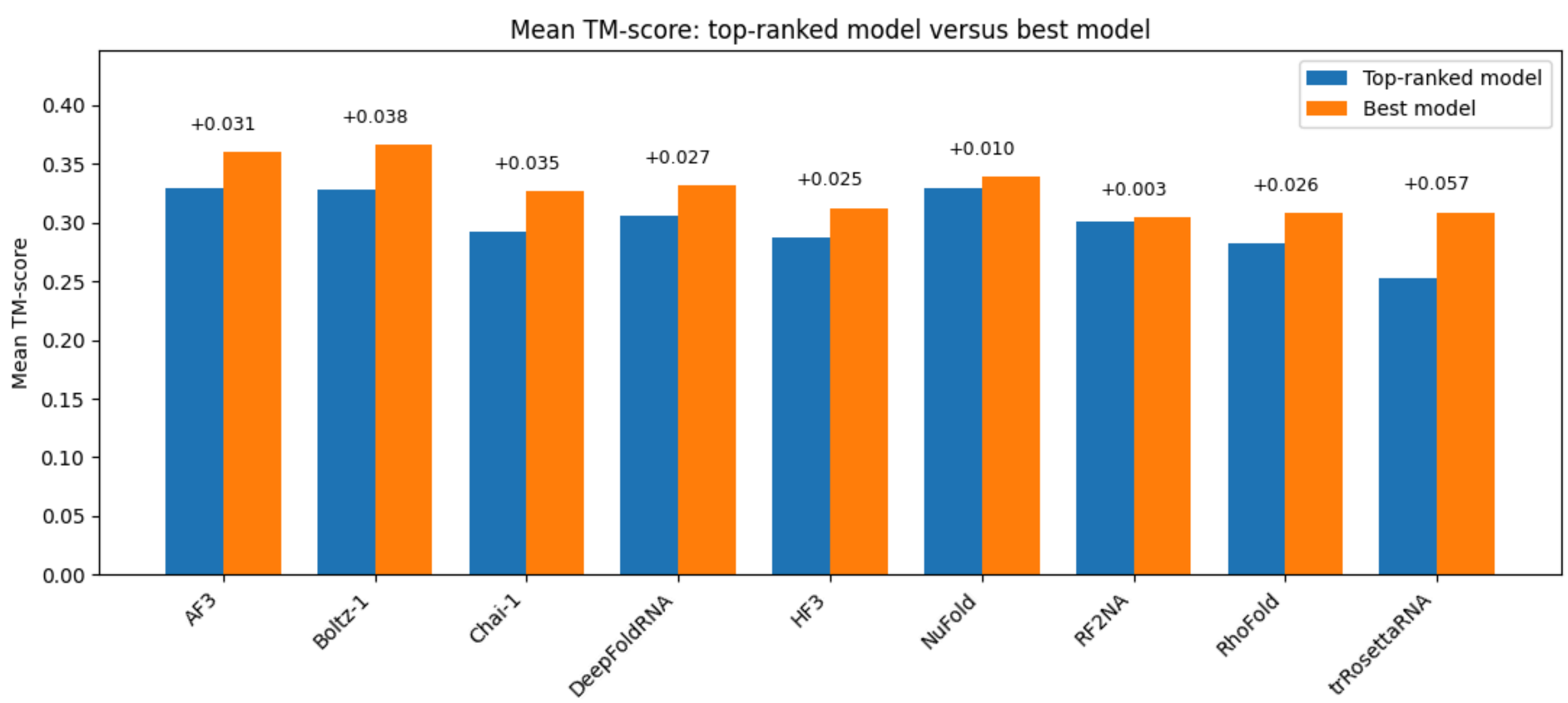

Figure S5. Mean TM-score across all methods by choosing the top-ranked (or the first-generated) model (left blue) compared to choosing the best generated model (right orange). Differences in the mean TM-score are minor across all methods.

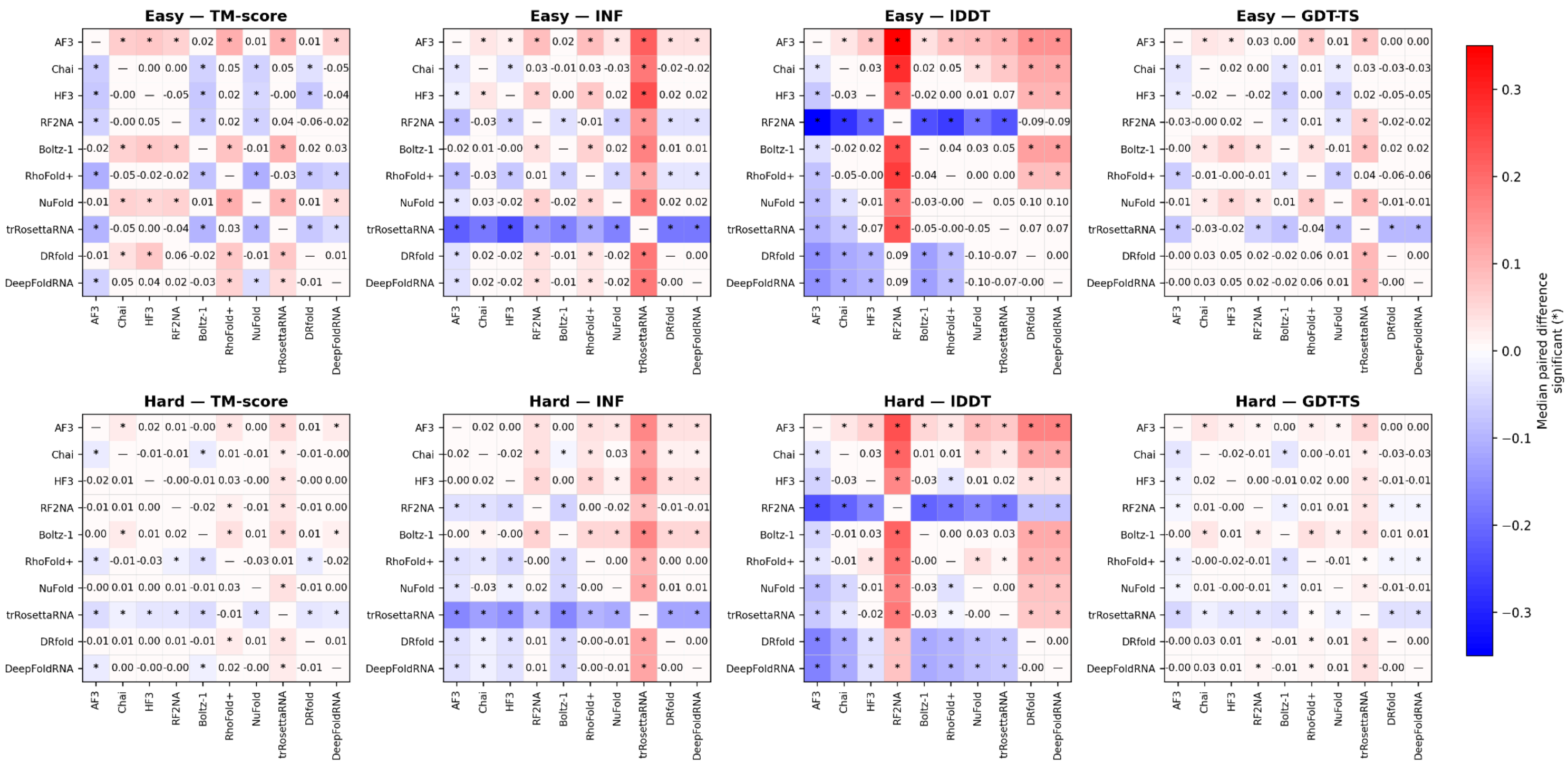

Figure S6. Wilcoxon rank-signed test results for TM-score and INF on easy and hard monomeric RNA datasets. Significant differences between the benchmarked methods are highlighted with an asterisk (*).

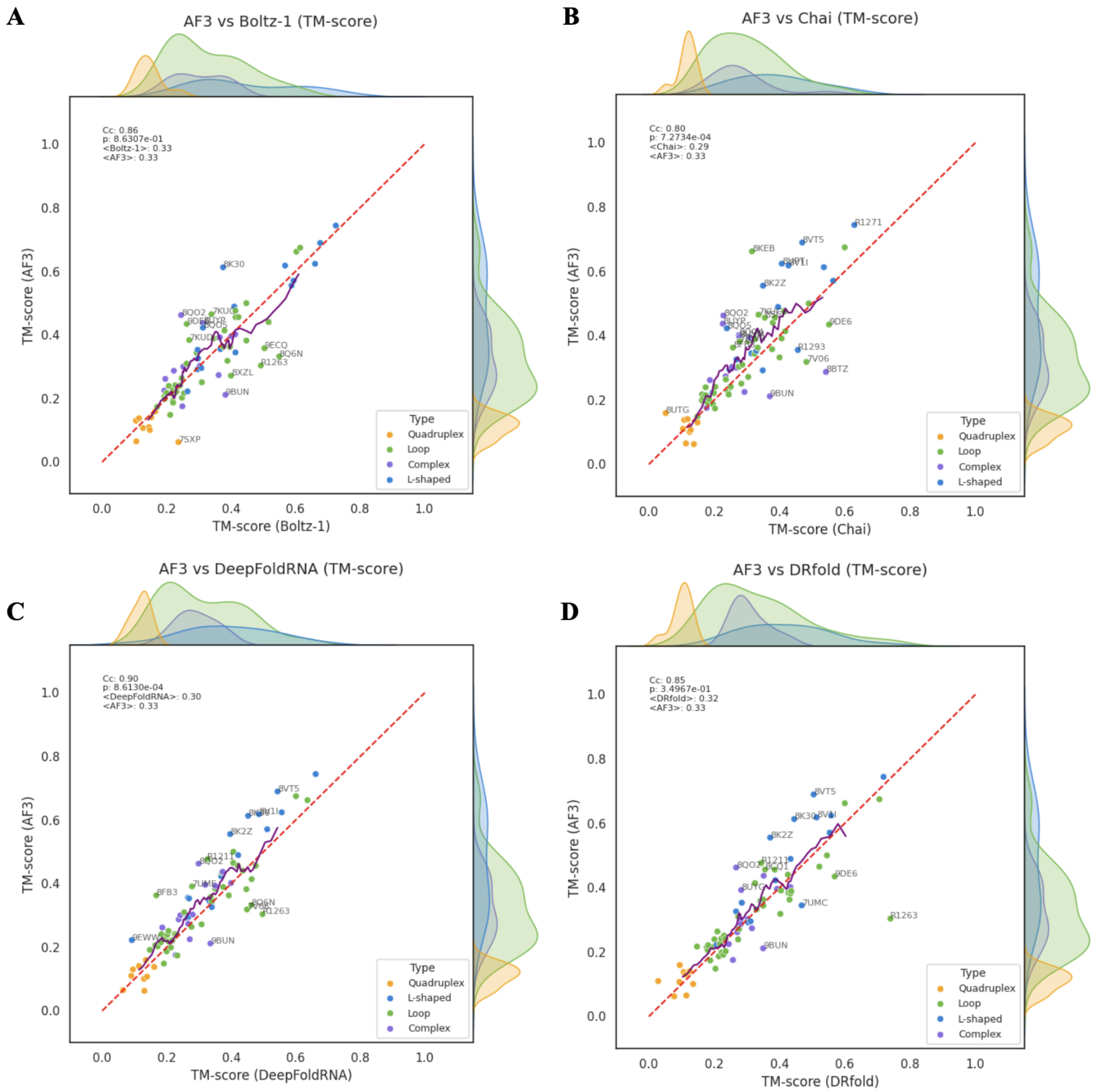

Figure S7. Pair plots showing TM-score correlation between AF3 against TM-scores of other methods: (A) Boltz-1, (B) Chai-1, (C) DeepFoldRNA and (D) DRfold. Cc denotes the Pearson correlation coefficient, paired T-test p-value by *p*.

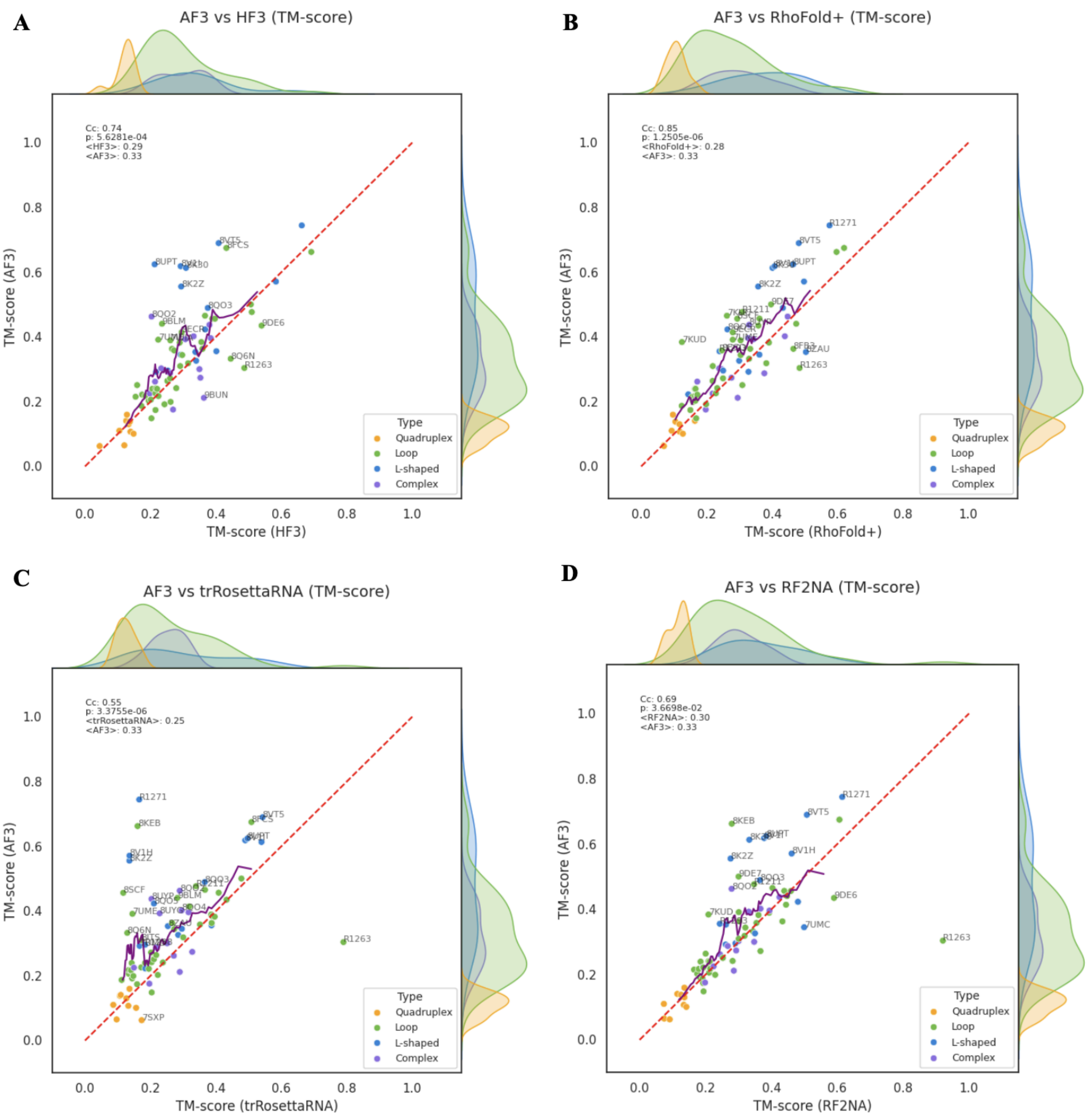

Figure S8. Pair plots showing TM-score correlation between AF3 against TM-scores of other methods: (A) HF3, (B) RhoFold+, (C) trRosettaRNA and (D) RF2NA. Cc denotes the Pearson correlation coefficient, paired T-test p-value by *p*.

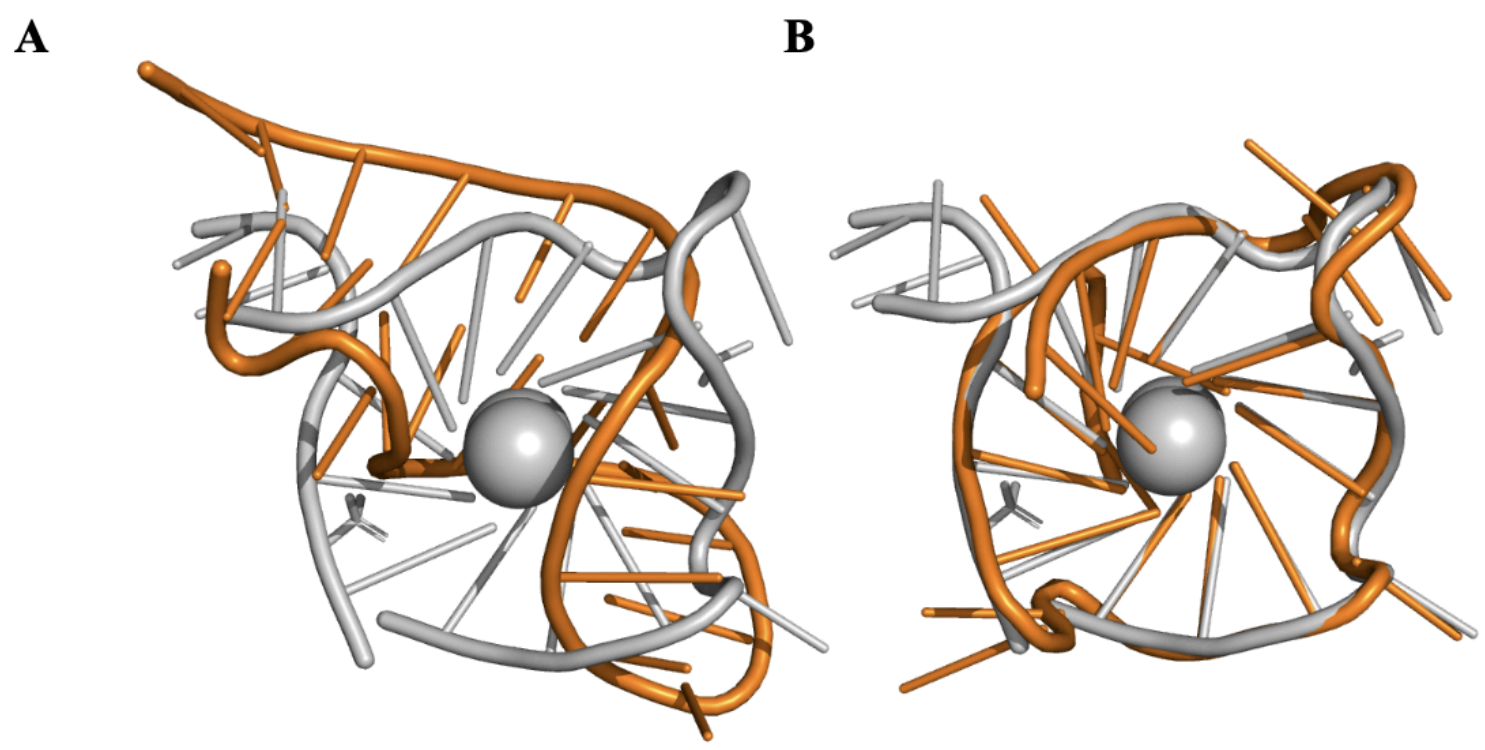

	**8VT5** (AF3)	**8FCS** (Boltz-1)
TM-score	0.063	0.237
INF	0.29	0.42
GDT-TS	0.34	0.79
lDDT	0.26	0.2
RMSD	10.528	0.796

Figure S9. Comparison of modelling 8VT5 between (A) AlphaFold 3 and (B) Boltz-1 where Boltz-1 predicted the structure with better accuracy, predicting the correct overall fold, while AlphaFold 3 failed.

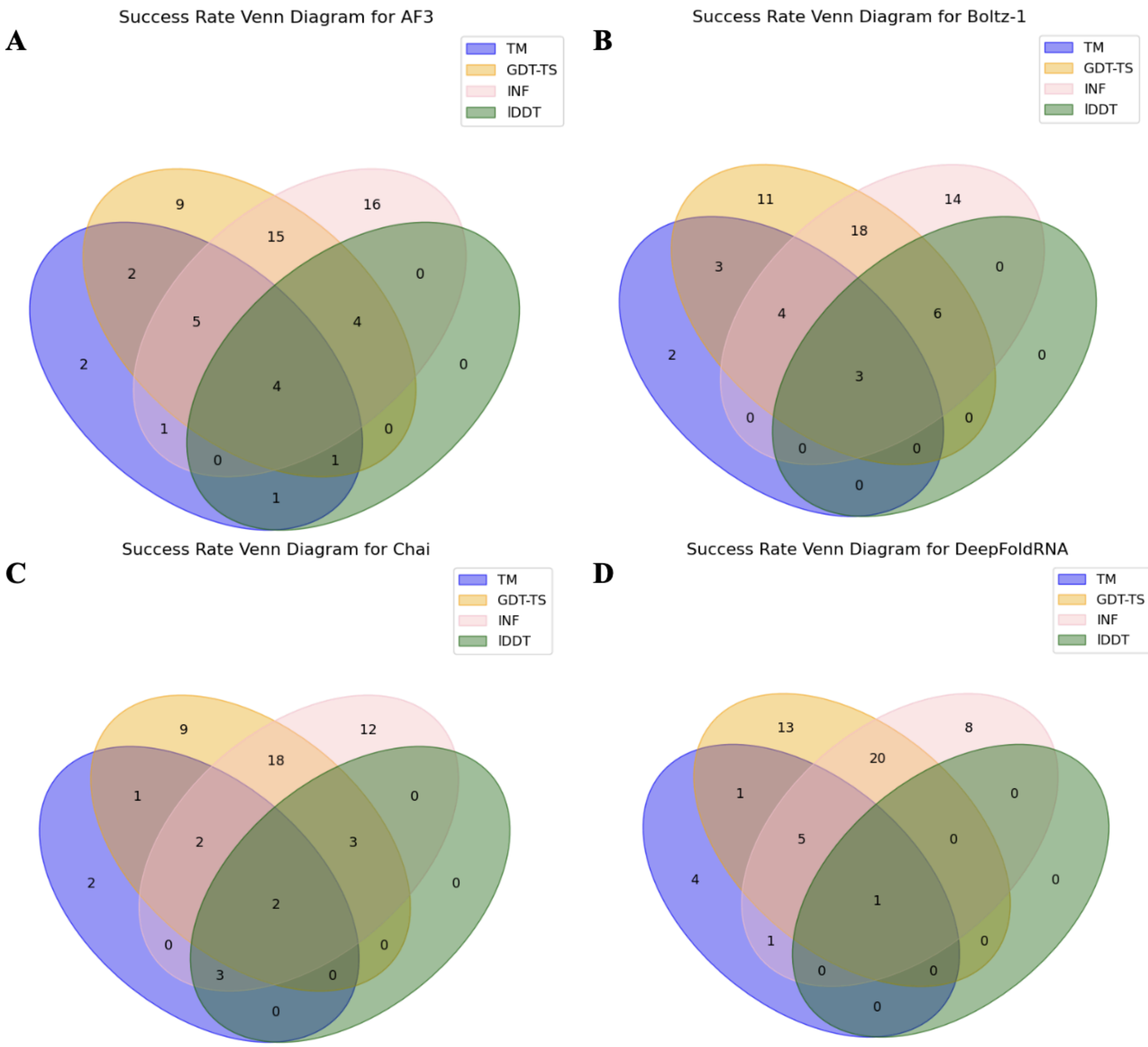

Figure S10. Venn diagrams showing the number of successful models based on the thresholds of different evaluation metrics, for (A) AF3, (B) Boltz-1, (C) Chai-1 and (D) DeepFoldRNA

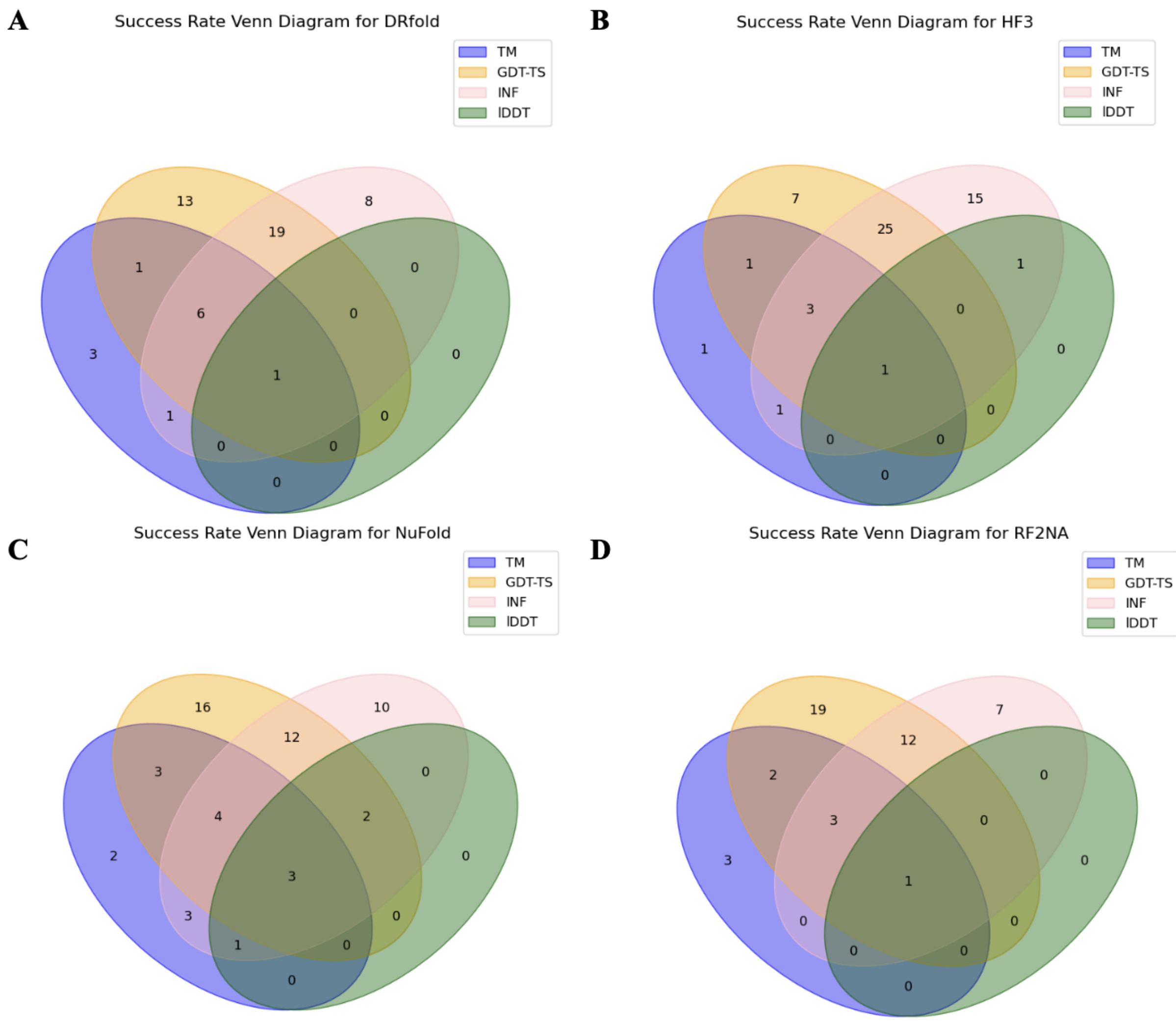

Figure S11. Venn diagrams showing the number of successful models based on the thresholds of different evaluation metrics, for (A) DRfold, (B) HF3, (C) RhoFold+, (D) RF2NA

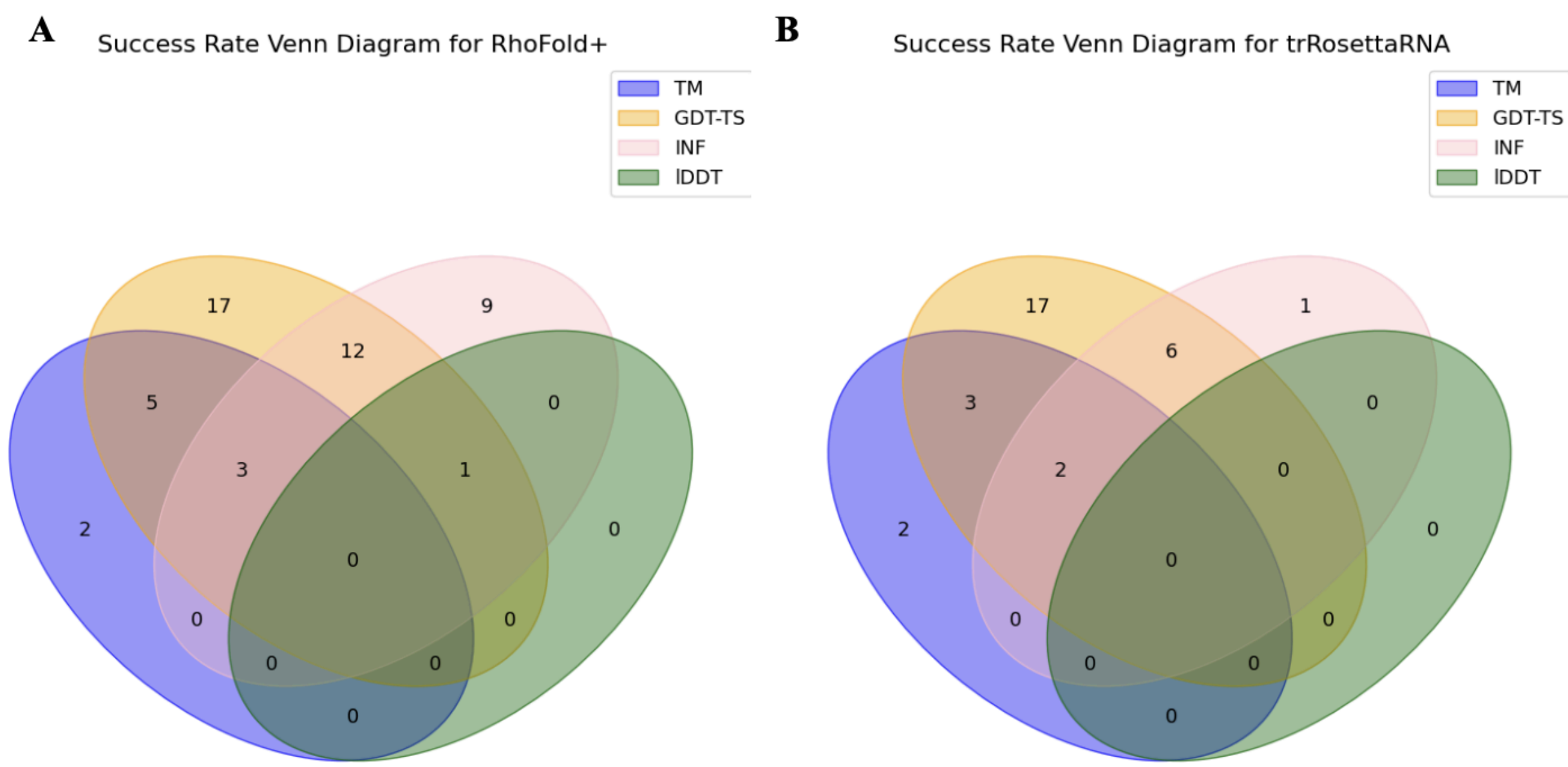

Figure S12. Venn diagrams showing the number of successful models based on the thresholds of different evaluation metrics, for (A) RhoFold+, (B) trRosettaRNA

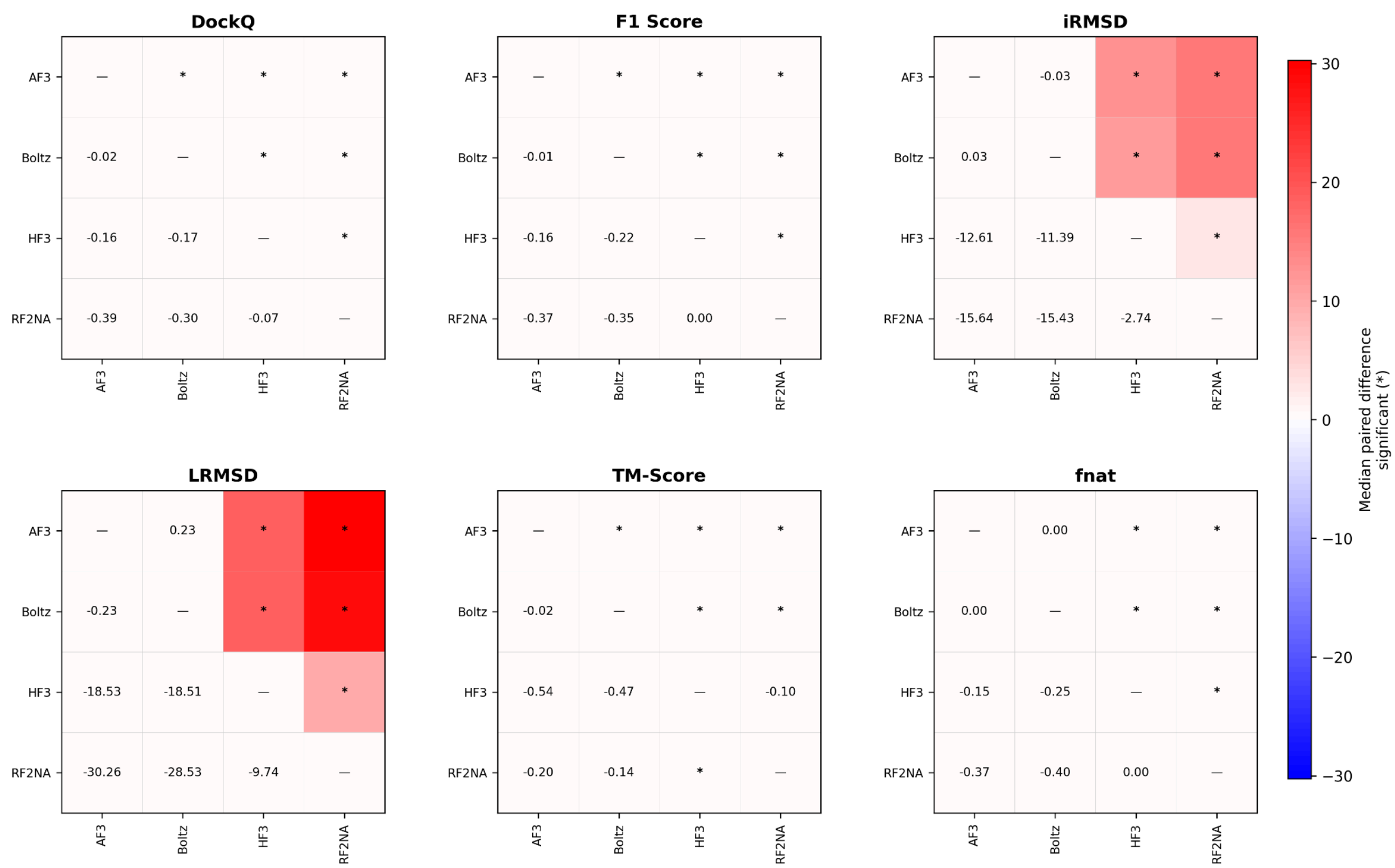

Figure S13. Wilcoxon signed-rank test results for the easy dataset of RNA-RNA and RNA-protein complexes. No significant differences were found between AF3 and Boltz-1, whereas HF3 and RF2NA were significantly worse than AF3 and Boltz-1 across all evaluation metrics.

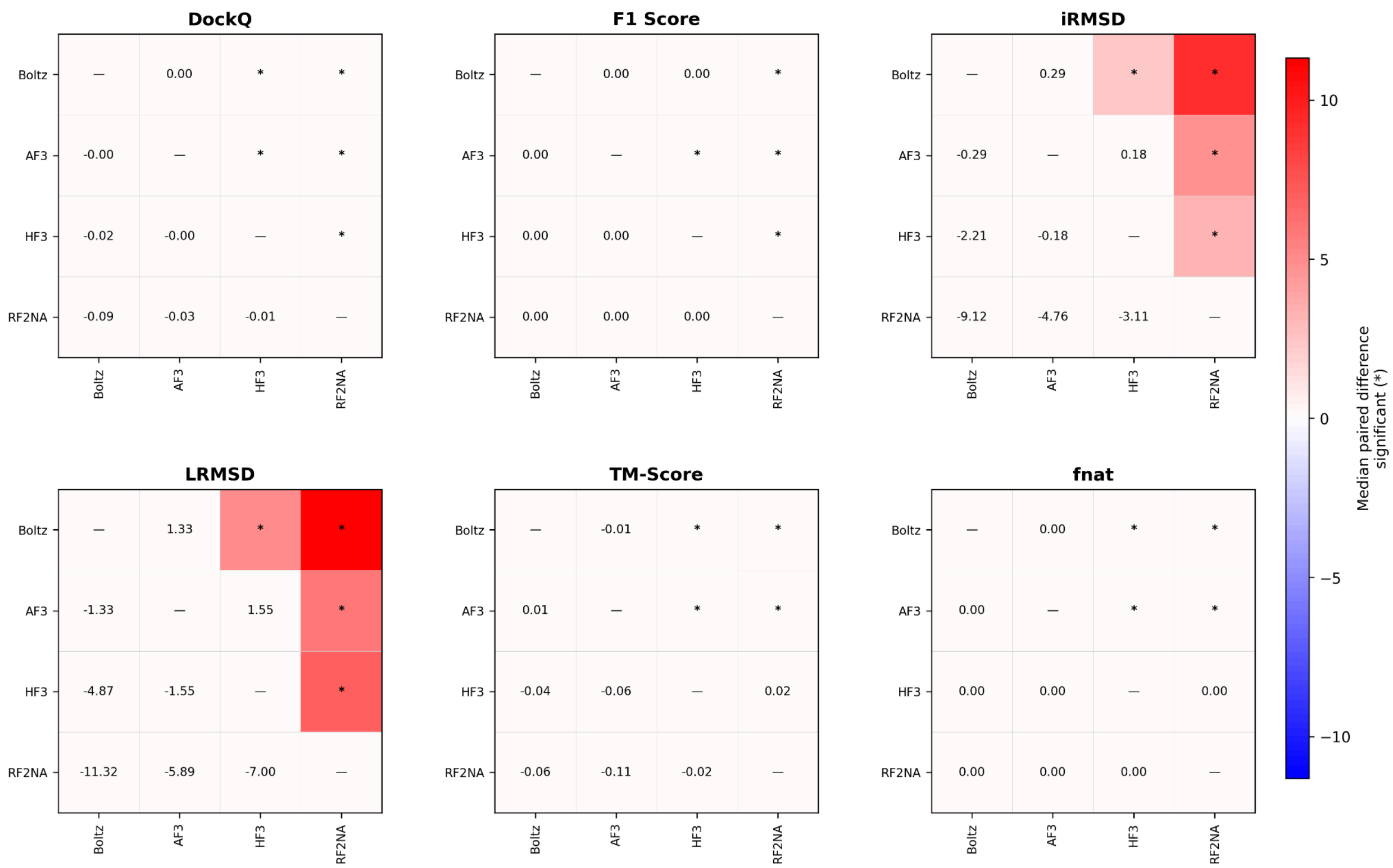

Figure S14. Wilcoxon signed-rank test results for the hard RNA-RNA and RNA-protein complex dataset. No significant differences were found between AF3 and Boltz-1 for all evaluation metrics. No significant differences were found between AF3 and Boltz-1, whereas HF3 and RF2NA were significantly worse than AF3 and Boltz-1 across all evaluation metrics.

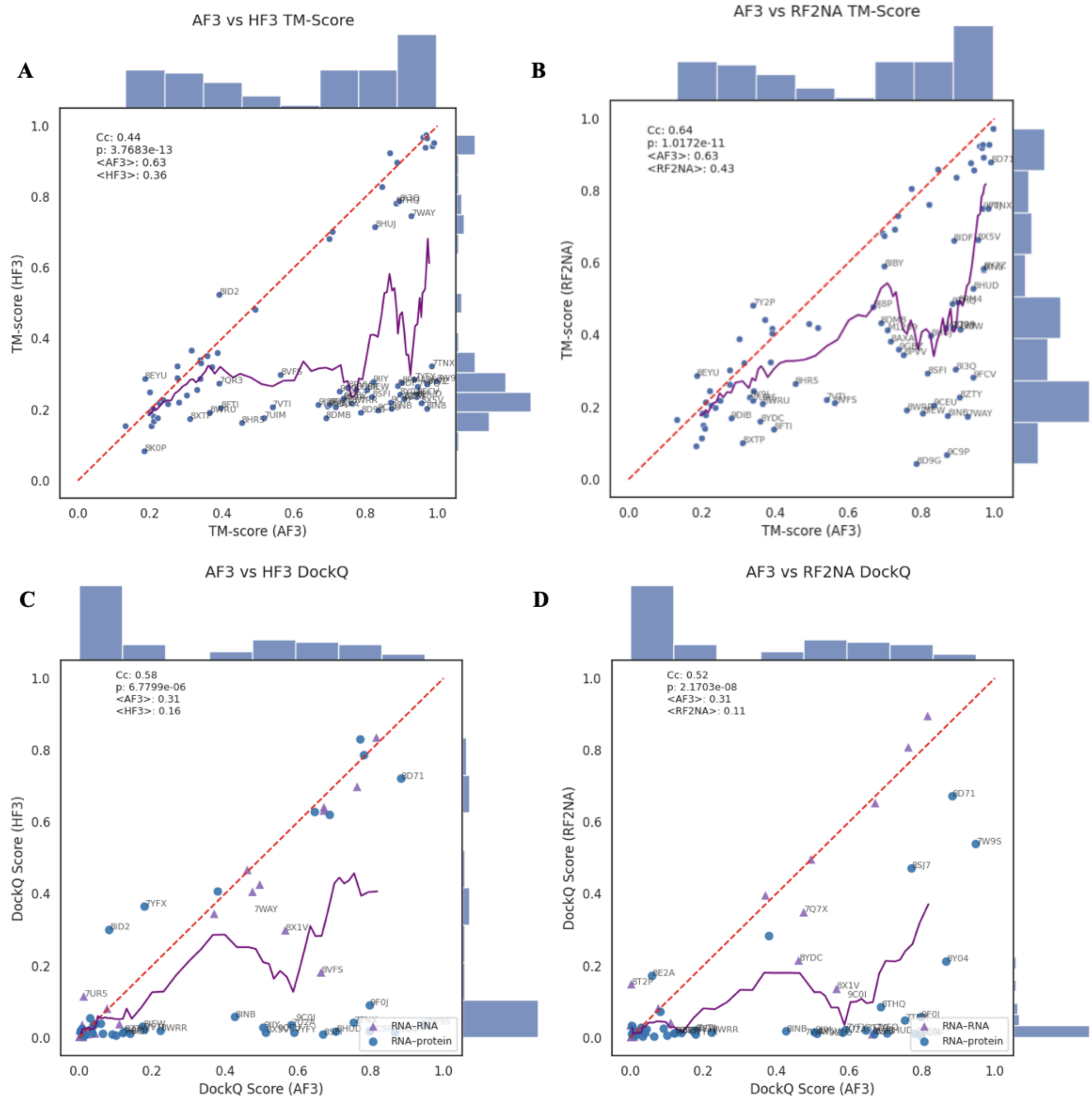

Figure S15. Scatter plots showing (A, B) TM-score and (C, D) Interface DockQ score correlation between the top-ranked method (AlphaFold3) against the corresponding scores of other methods. Cc denotes the Pearson correlation coefficient, and the paired T-test p-value by p. Significant differences in TM-scores and interface DockQ scores were found between AlphaFold3 and Boltz-1, HF3, and RF2NA, for both TM-score and interface DockQ score.

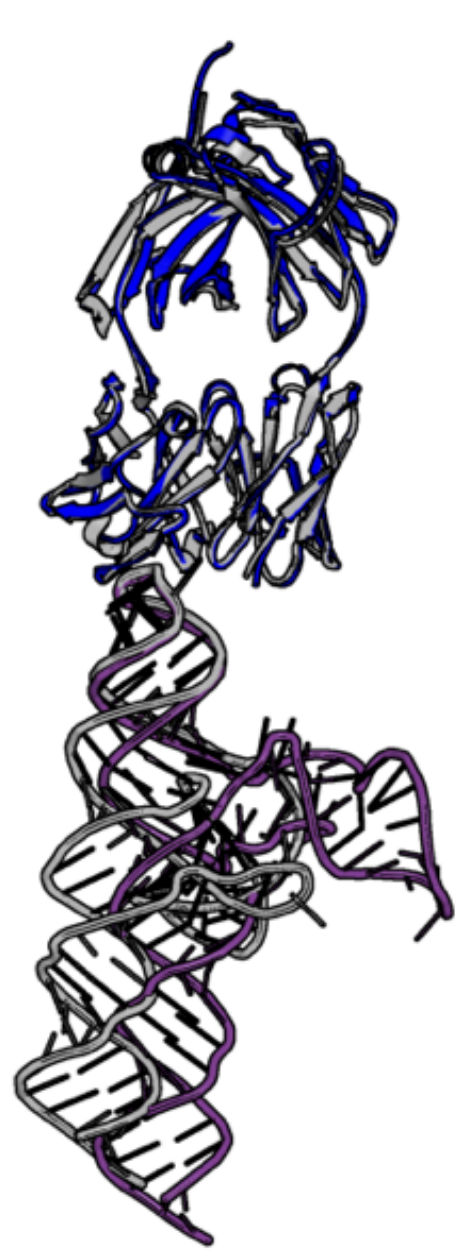

8T29 (HF3)
RNA-protein DockQ 0.670
RNA-RNA DockQ /
Total DockQ 0.713
TM-score 0.887

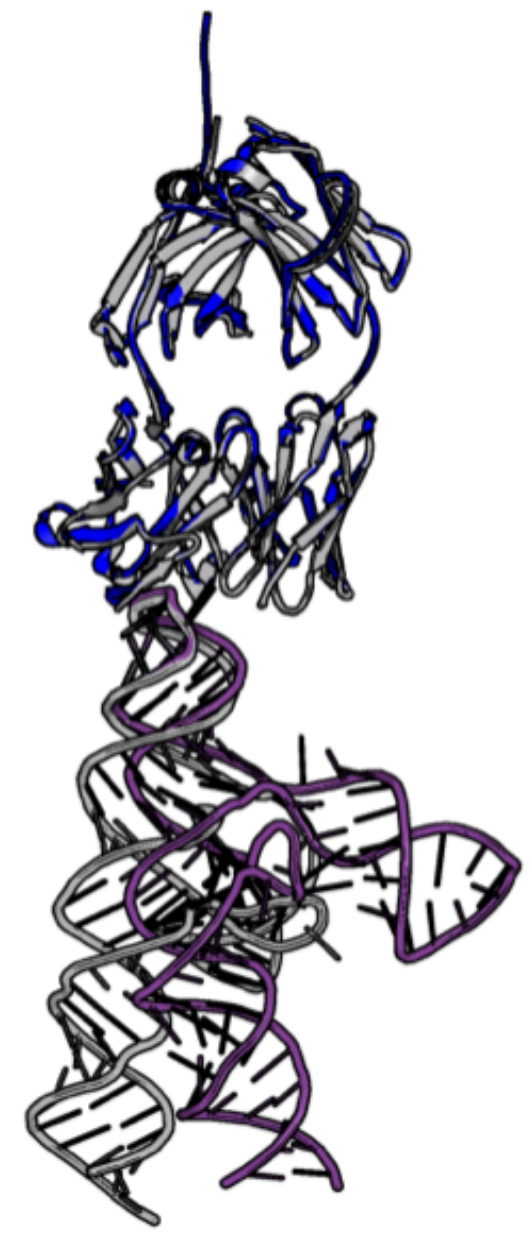

8T29 (AF3)
RNA-protein DockQ 0.647
RNA-RNA DockQ /
Total DockQ 0.724
TM-score 0.886

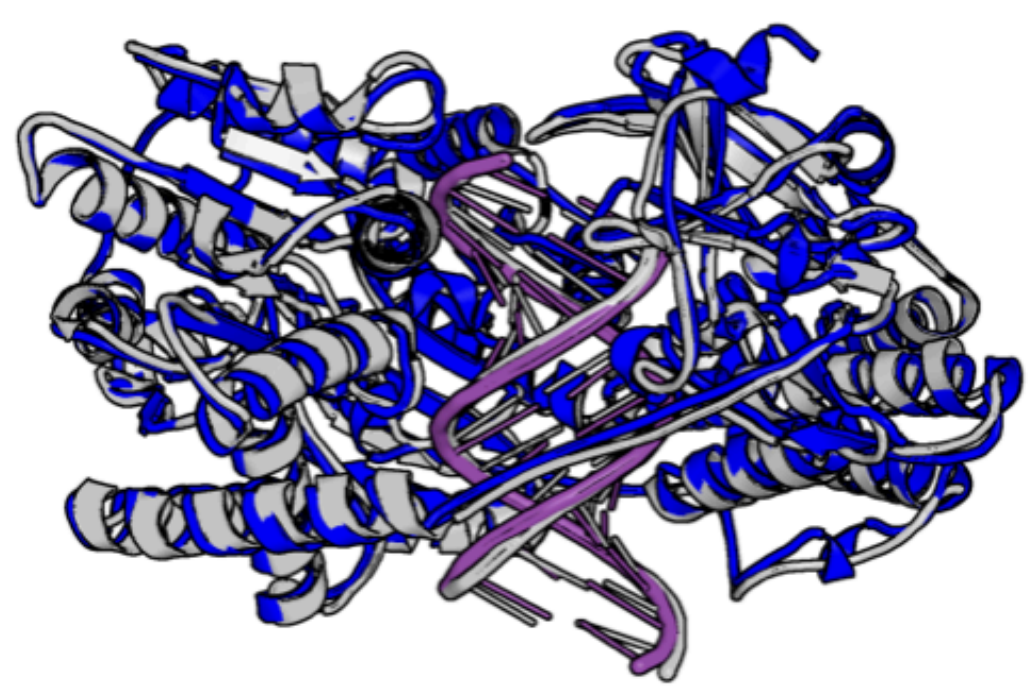

7TNY (Boltz-1)
RNA-protein DockQ 0.532/0.592
RNA-RNA DockQ 0.894
Total DockQ 0.673
TM-score 0.974

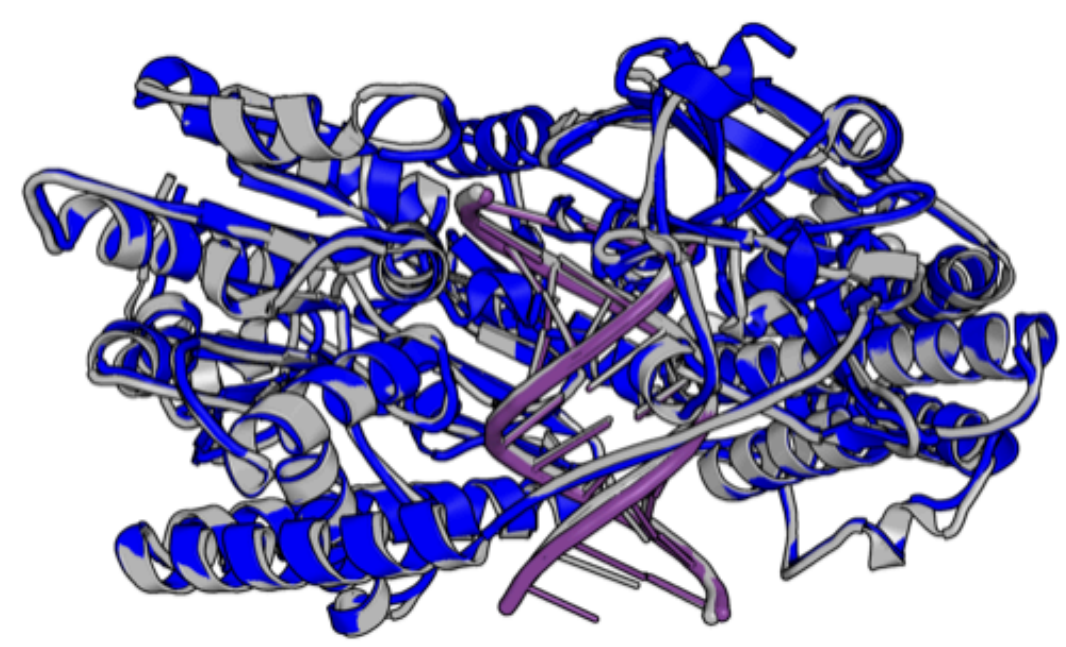

7TNX (AF3)
RNA-protein DockQ 0.754/0.805
RNA-RNA DockQ 0.883
Total DockQ 0.814
TM-score 0.984

Figure S16. Visualization of the top 2 predicted interfaces for both RNA-protein and RNA-RNA interfaces. Top row shows the top 2 predicted RNA-protein interfaces (PDB 8T29 HF3 and 8T29 AF3), while the bottom row shows the top 2 predicted RNA-RNA interfaces (PDB 7TNY Boltz-1 and 7TNX AF3) with IF-DockQ scores shown in bold.

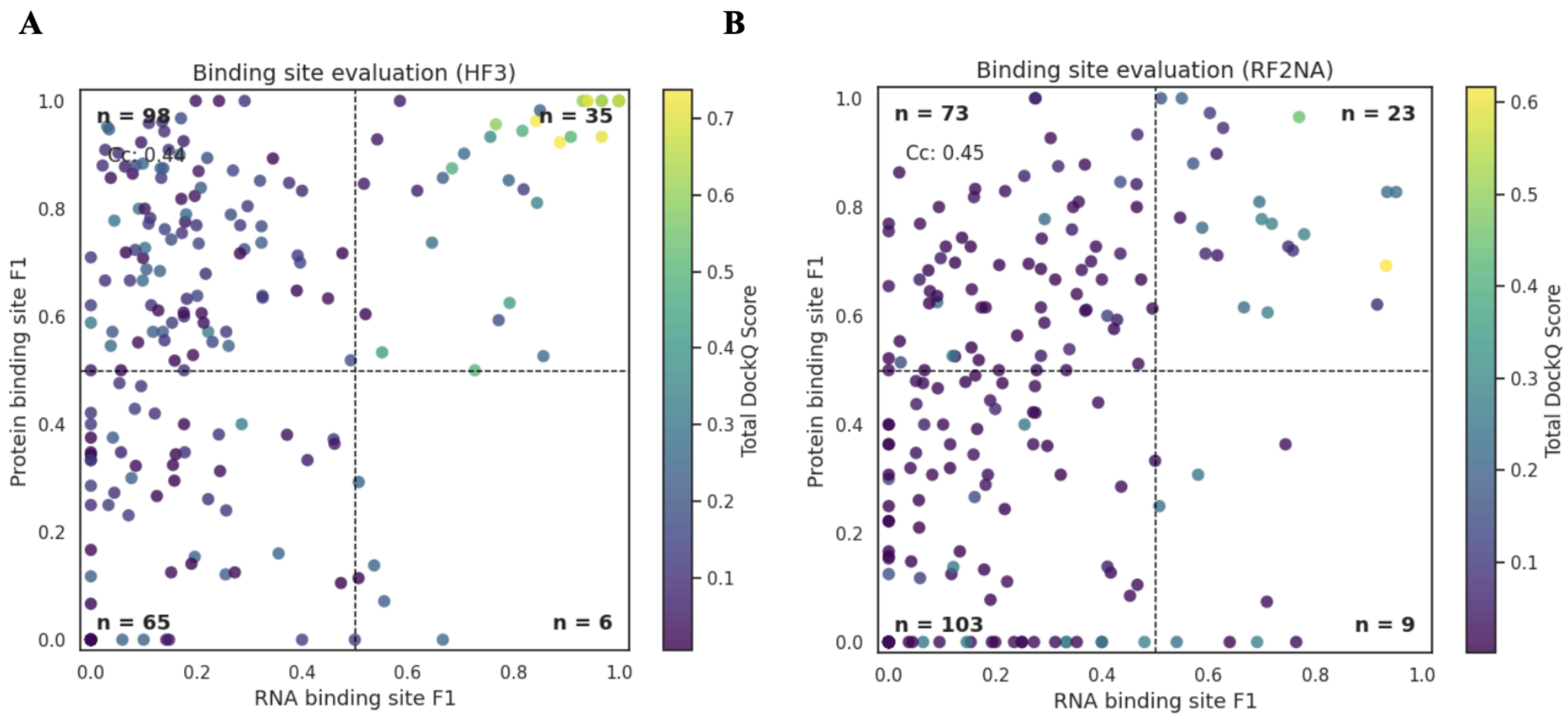

Figure S17. Scatter plots showing the correlation between RNA and protein binding site F1 score, colored by DockQ score for each RNA-protein interface, for (A) HF3 and (B) RF2NA. The plot is divided into four quadrants and the number of interfaces is counted and shown in bold.

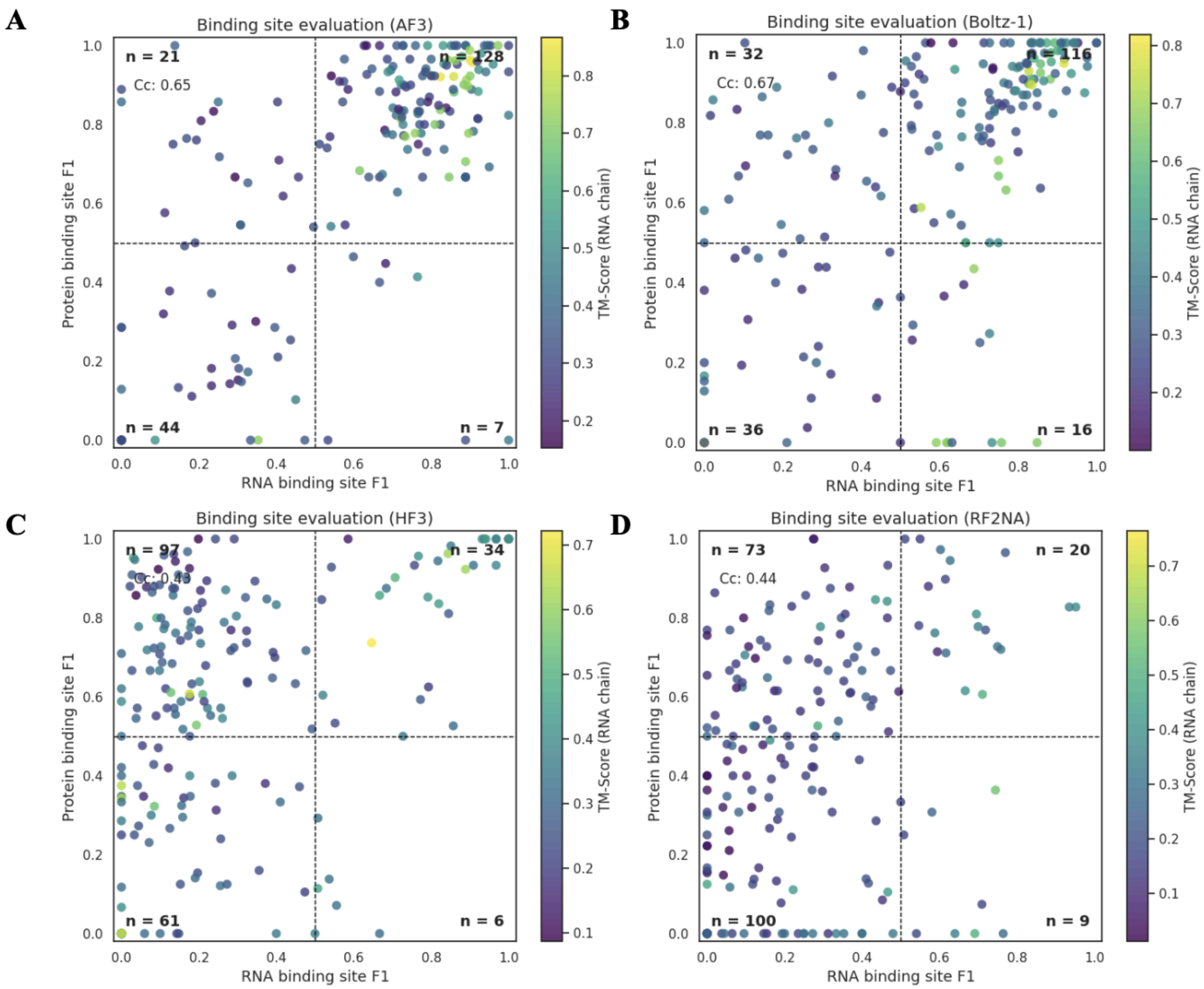

Figure S18. Scatter plots showing the correlation between RNA and protein binding site F1 score, colored by TM-score for RNA chains for each RNA-protein interface, for (A) AF3, (B) Boltz-1, (C) HF3, (D) RF2NA. The plot is divided into four quadrants and the number of interfaces is counted and shown in bold.

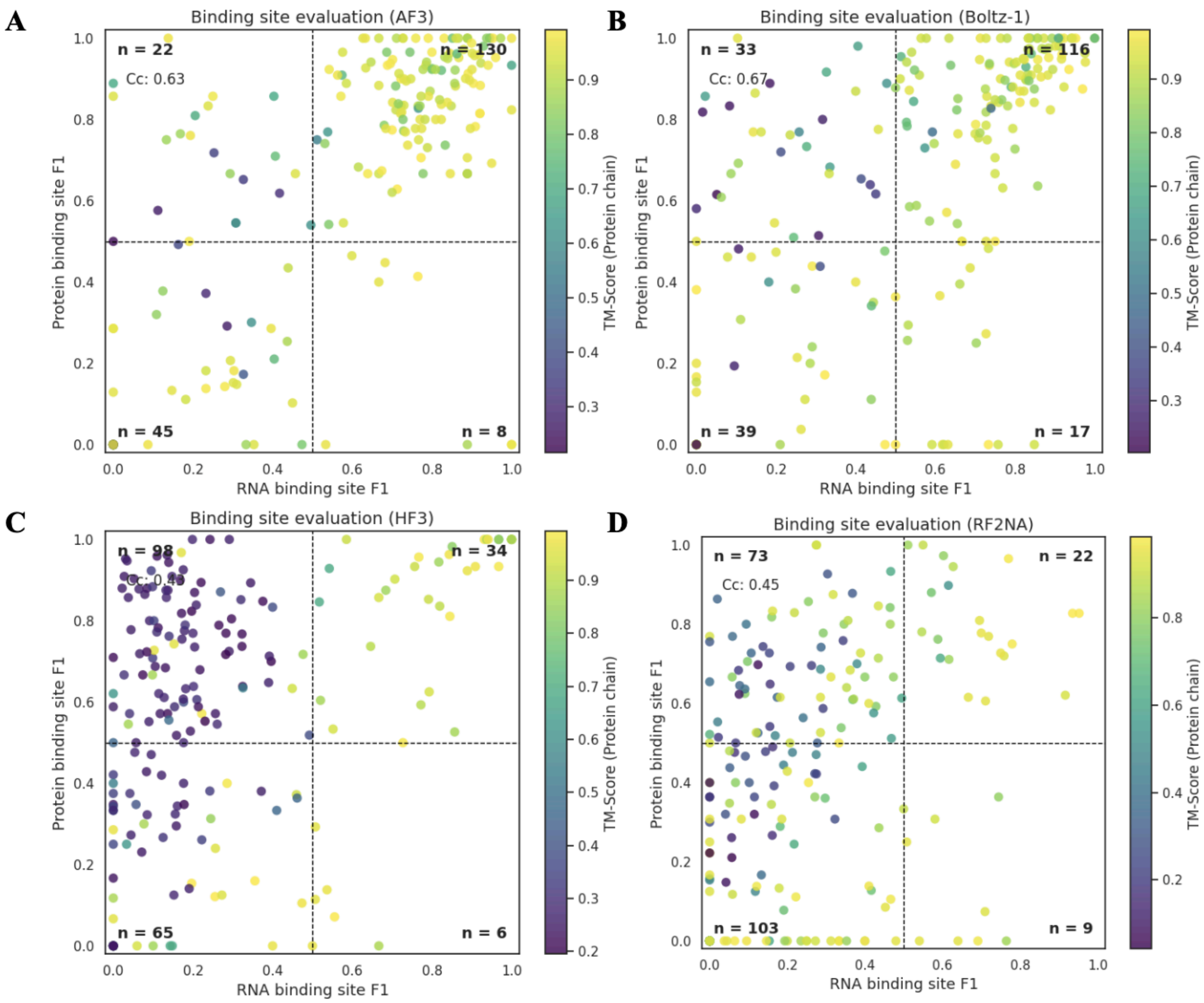

Figure S19. Scatter plots showing the correlation between RNA and protein binding site F1 score, colored by TM-score for protein chains for each RNA-protein interface, for (A) AF3, (B) Boltz-1, (C) HF3, (D) RF2NA. The plot is divided into four quadrants and the number of interfaces is counted and shown in bold.

Length-Dependent Limitations of TM-score in RNA Structure Evaluation

While examining success rates, we observed that among the two global fold metrics (GDT-TS and TM-score), GDT-TS consistently yields substantially higher success rates than TM-score across all methods. This was observed for both the easy and hard datasets. In other words, many models that are classified as correct by GDT-TS are not considered successful by TM-score. To better understand this discrepancy, we examined structures with high GDT-TS but low TM-score. The four most pronounced examples are shown in Figure S19. These structures also exhibit high INF values, indicating well-captured base pairing, stacking, and local interactions. Therefore, low TM-scores are not necessarily reflective of a poor structural quality, but rather a limitation of the TM-score when applied to short RNA chains. This behaviour comes from normalisation by the length-dependent scaling factor (d_0) used in the TM-score formulation (Gong et al. 2019). For short RNAs (e.g., < 40 nt), the scaling factor becomes so small that even minor structural deviations lead to disproportionately low TM-scores.

A previous RNA benchmarking study (Nithin et al. 2024) also noted this issue and addressed it by excluding all RNAs shorter than 60 nucleotides from their benchmark. However, we consider this filtering undesirable for several reasons. Many biologically important RNAs are short yet structured, and excluding them removes a meaningful part of RNA structural space. Moreover, because the methods evaluated in this benchmark were trained on the entire PDB, which includes numerous RNAs shorter than 60 nt, excluding short RNAs from the benchmark would prevent a fair assessment of model performance on sequences that were part of their training distribution.

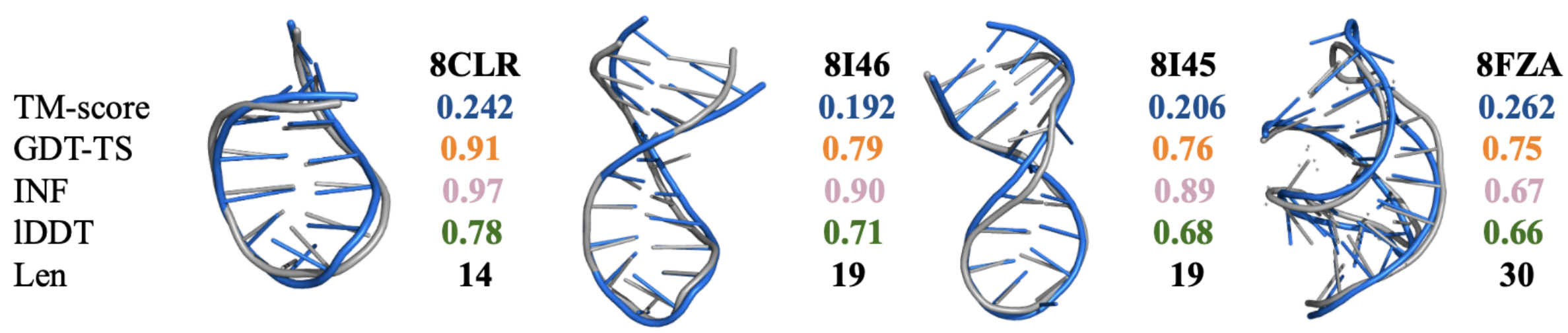

Figure S20. Four models with a higher GDT-TS and INF, but very low TM-score. The ground-truth structures are colored in grey, while predicted structures are shown in blue.

To further investigate the influence of RNA length on structure accuracy, we analysed pairwise relationships among the four evaluation metrics. Figure S20 shows all six pairwise correlations with data points colored by RNA length. The dataset is divided into three length intervals: short RNAs (13-28 nt), medium RNAs (29-68 nt), and long RNAs (69-497 nt). Dark blue points correspond to short RNA structures, lighter blue medium-sized RNAs and red points to the long RNAs. Grey dashed lines indicate the thresholds used to define the success rate for each metric.

Comparing correlations between TM-score and the other three metrics, a pronounced length-dependent effect is observed. Many short models fall into the lower-right quadrant, i.e., exceeding the GDT-TS (Figure S20A) and INF (Figure S20B) thresholds but being below the TM-score threshold. This indicates that short RNA models achieve relatively high GDT-TS and INF values despite low TM-scores. In contrast, as RNA length increases towards medium-sized and long RNAs, the number of models in this

quadrant also decreases. This trend is also noticeable in the distribution of TM-scores, which shifts towards higher values for longer RNAs. Consequently, TM-score shows stronger agreement with other evaluation metrics for longer RNAs, and models that exceed GDT-TS or INF threshold are more likely to also exceed the TM-score threshold.

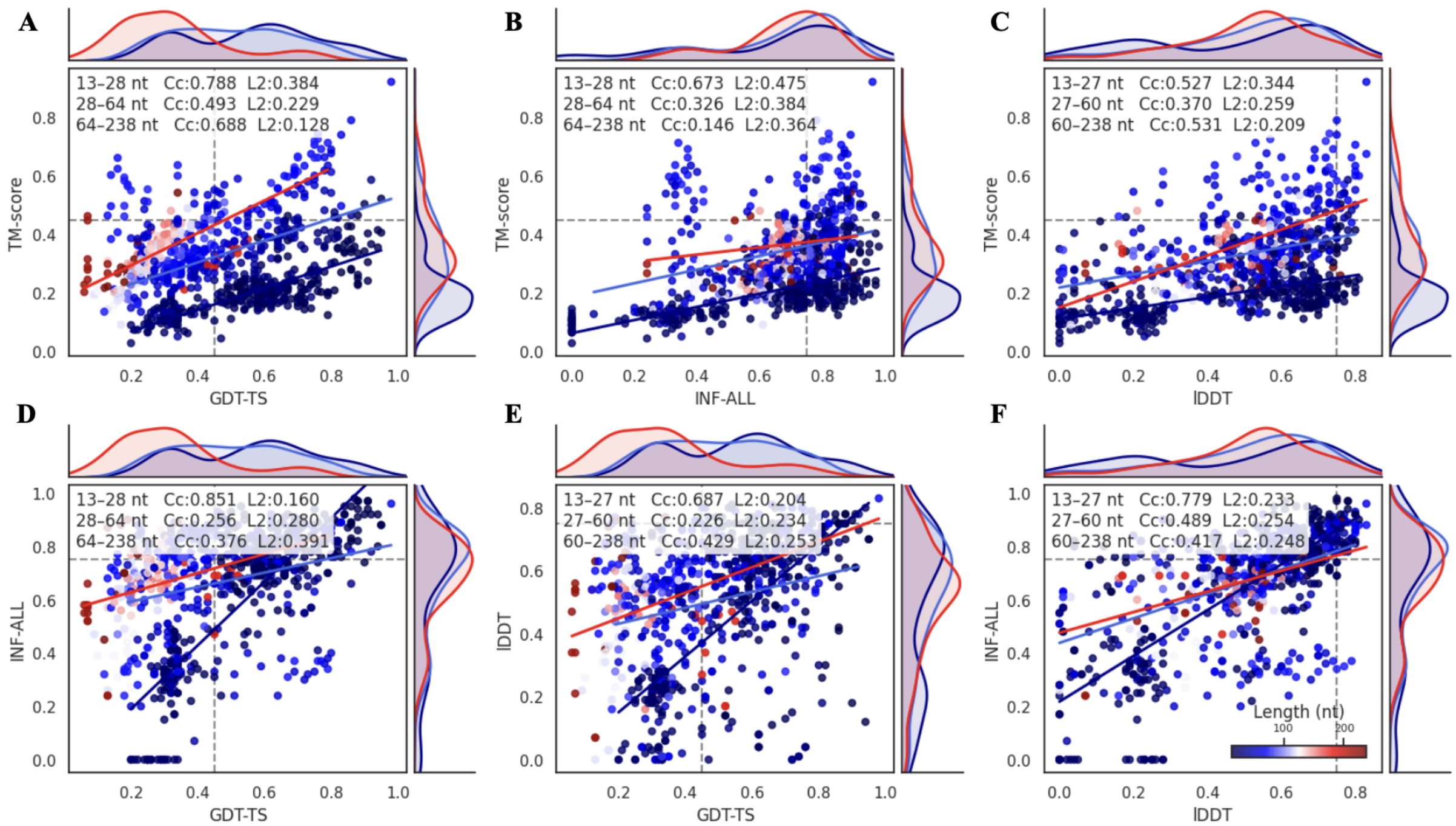

Figure S21. Pairwise comparison of evaluation metrics. (A) Correlation between TM-score and GDT-TS. (B) Correlation between TM-score and lDDT (C) Correlation between TM-score and INF (INF-ALL). (D) Correlation between INF and GDT-TS (E) Correlation between lDDT and GDT-TS. (F) Correlation between INF and lDDT. Each plot is divided into three length intervals. Cc denotes the Pearson correlation coefficient with L2 error also reported.

Correlations between other pairs of metrics are also shown in Figure S20, with the strongest agreement observed between GDT-TS and INF (Figure S20D) and lDDT and INF (Figure S20F), both of which emphasise local structural accuracy. However, the threshold for lDDT is significantly stricter than that for INF, as many models have INF scores around 0.8 and lDDT scores around 0.6. This suggests that some models may still contain suboptimal local geometries. INF is the only metric entirely length independent, as observed from the INF score overlapping distributions for each length interval. On the other hand, GDT-TS shows a bimodal distribution for the shortest RNAs. Overall, this analysis highlights the importance of evaluating RNA structure predictions using multiple complementary metrics.

Furthermore, we performed a sensitivity analysis by calculating the TM-score using different values of the -d scaling factor, ranging from 1 to 5. The corresponding scatter plots are shown in Figures S21. The weakest correlations were observed when TM-score was calculated using the default scaling factor (Figure S21A), with correlations of only 0.279 with GDT-TS, 0.37 with INF, and 0.428 with lDDT. On the other hand, the strongest correlations were observed between TM-score calculated with -d 1 and GDT-TS (0.867), TM-score calculated with -d 4 and INF (0.503), and TM-score calculated with -d 5 and lDDT (0.508). Additionally, correlations and L2 errors between TM-scores calculated with fixed -d values from 1 to 5 and the other evaluation metrics were broadly similar, with no significant differences observed among the different fixed -d settings (Figure S21). These results suggest that using a fixed scaling factor, rather than the default length-dependent scaling, yields a more consistent estimate of model accuracy and leads to stronger agreement with other evaluation metrics.

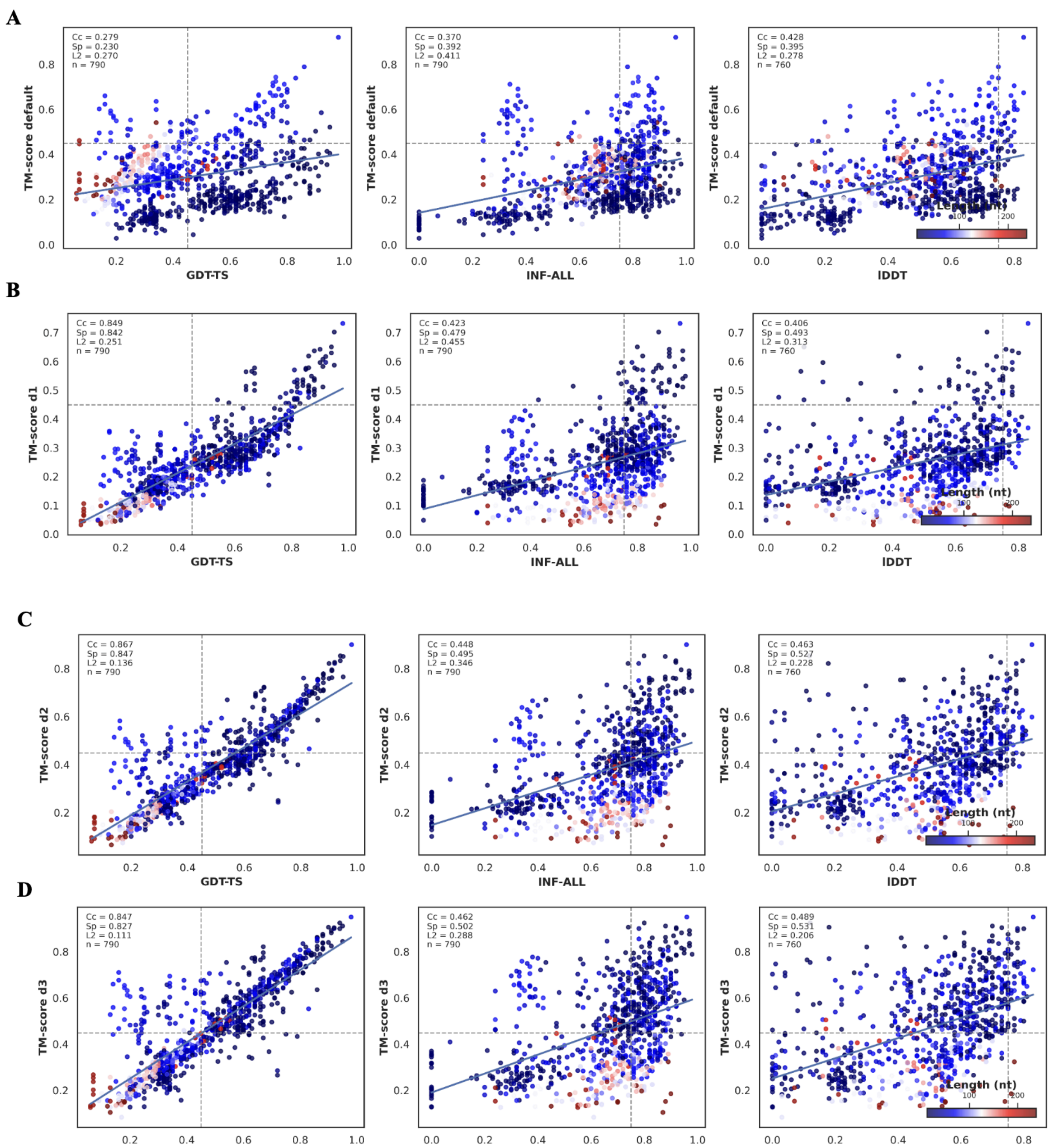

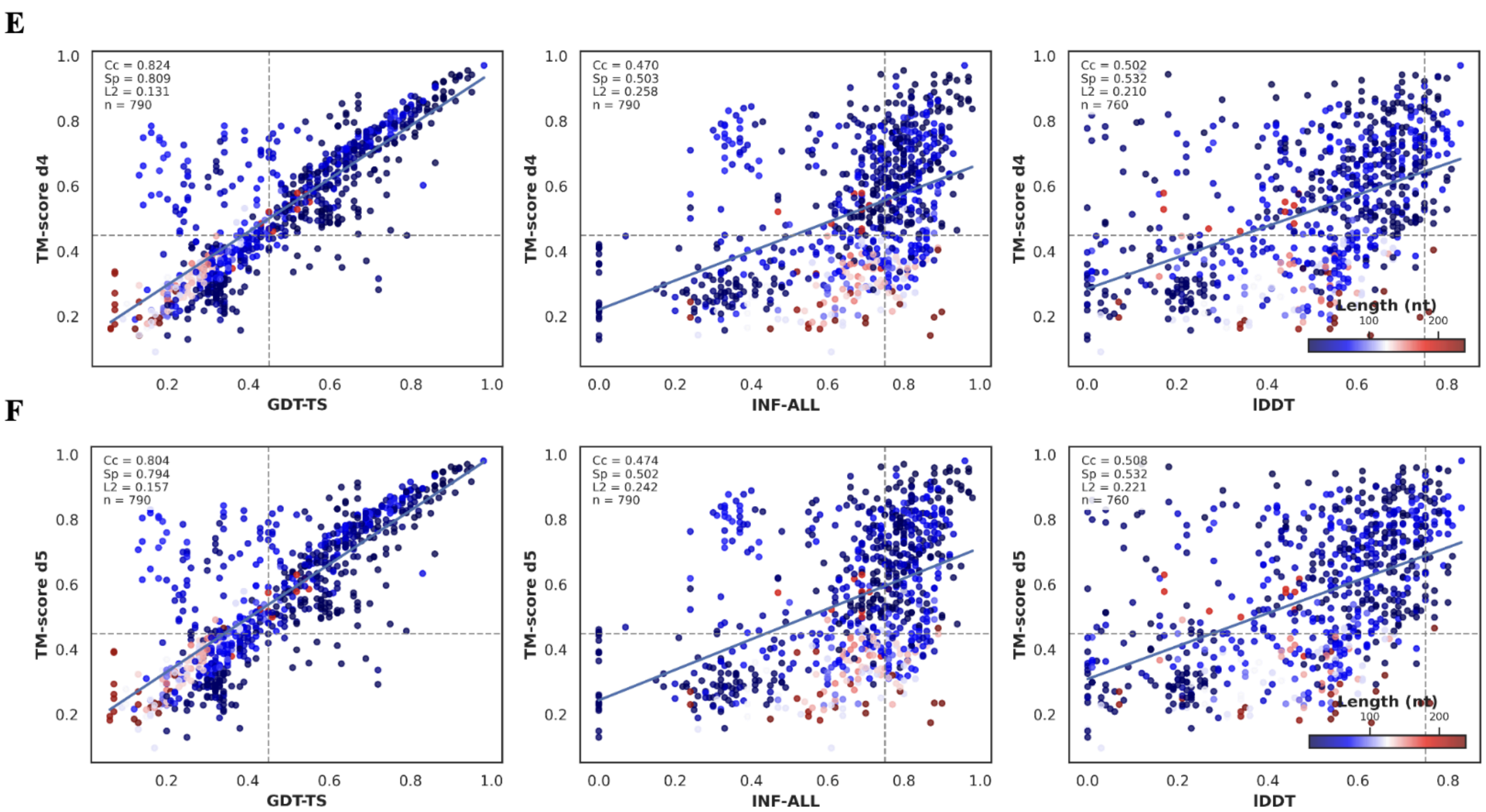

Figure S22. Pairwise comparison of evaluation metrics with different TM-score scaling factors. (A) Correlation between TM-score (default) and other metrics (GDT-TS, INF-ALL, lDDT). (B) Correlation between TM-score (-d 1) and other metrics. (C) Correlation between TM-score (-d 2) and other metrics. (D) Correlation between TM-score (-d 3) and other metrics. (E) Correlation between TM-score (-d 4) and other metrics. (F) Correlation between TM-score (-d 5) and other metrics.

In addition, we performed a Wilcoxon signed-rank test to assess whether the relative performance of the methods differed when the TM-score was calculated with different "-d" factors. Our results show that, when using -d 1, there was no significant difference between the top 3 methods (AF3, Boltz-1, NuFold). For the remaining scaling factors (-d 2 to -d 5), as well as the default TM-score calculation, no significant differences were found between AF3, Boltz-1, NuFold and DRfold (Figure S22). Therefore, although using fixed scaling factors changed the absolute TM-score values, it did not substantially alter the statistical ranking of methods in our benchmark. For this reason, we retained the default TM-score calculation for the main analysis. We note that the length dependence of TM-score becomes unreliable for short RNAs, and the results in this range must be used carefully.

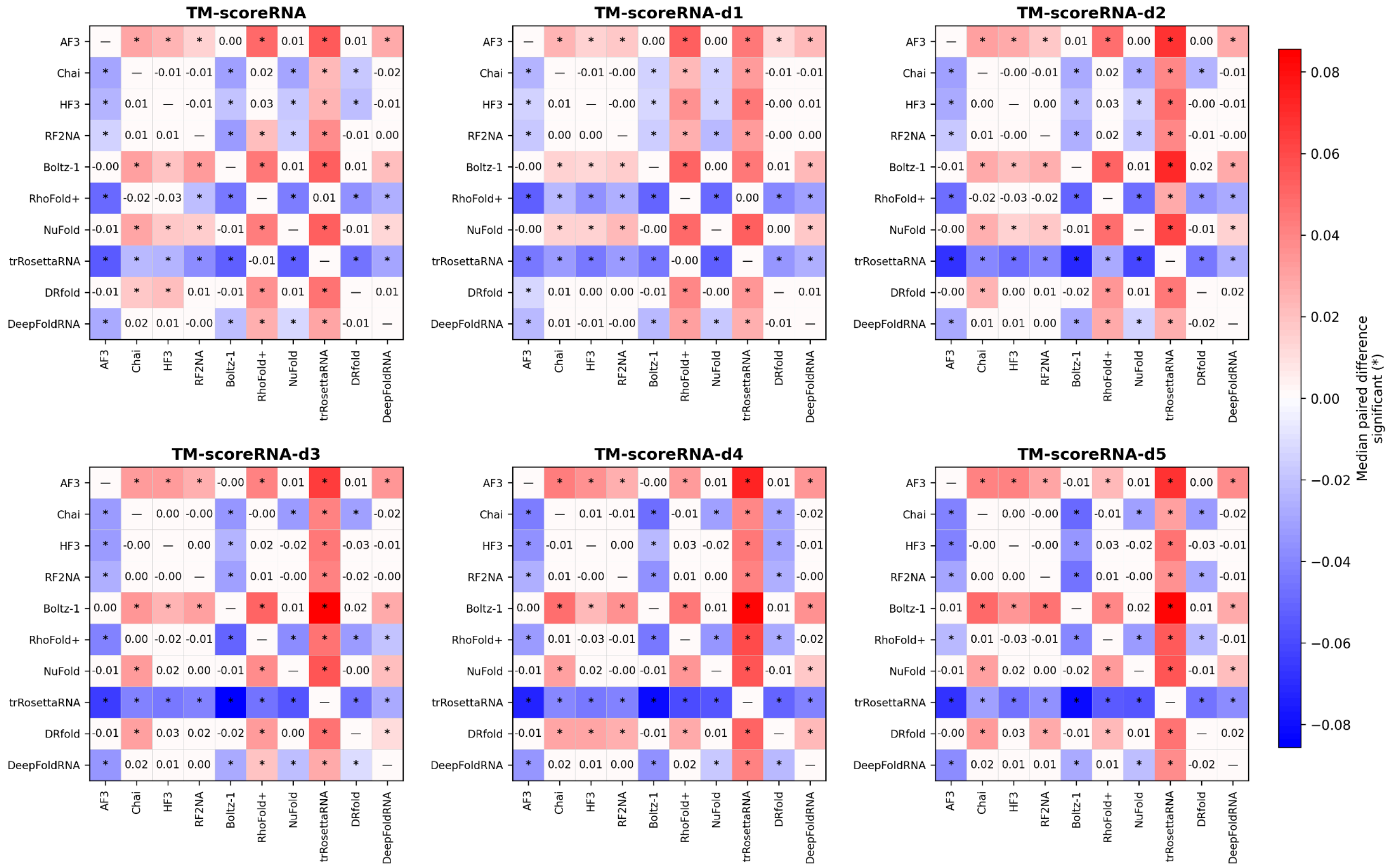

Figure S23. Wilcoxon signed-rank test for different TM-score scaling factors. In all cases, no significant difference was found between the top 3 methods (AF3, Boltz-1, NuFold).

References

Gong, Sha, Chengxin Zhang, and Yang Zhang. 2019. "RNA-Align: Quick and Accurate Alignment of RNA 3D Structures Based on Size-Independent TM-scoreRNA." *Bioinformatics (Oxford, England)* 35 (21): 4459–4461.

Nithin, Chandran, Sebastian Kmiecik, Roman Błaszczyk, Julita Nowicka, and Irina Tuszyńska. 2024. "Comparative Analysis of RNA 3D Structure Prediction Methods: Towards Enhanced Modeling of RNA-Ligand Interactions." *Nucleic Acids Research* 52 (13): 7465–7486.
